# Identification of three *Daphne* species by DNA barcoding and HPLC fingerprint analysis

**DOI:** 10.1371/journal.pone.0201711

**Published:** 2018-08-02

**Authors:** Yanpeng Li, Lu Geng, Yuanyan Liu, Mingyang Chen, Qirui Mu, Xu Zhang, Zhongyi Zhang, Guangxi Ren, Chunsheng Liu

**Affiliations:** 1 School of Chinese Pharmacy, Beijing University of Chinese Medicine, Beijing, PR China; 2 Pharmaceutical Co., Ltd., of Qinhuangdao Shanhai Pass, Qinhuangdao, Hebei Province, PR China; Tallinn University of Technology, ESTONIA

## Abstract

In order to well identify the 93 wild Cortex Daphnes samples from different species and habitats in western China and develop a standard operating procedure (SOP) for the authentication and quality of them in the future, a comprehensive and efficient identification system based on DNA barcoding and HPLC fingerprint technologies has been developed. The result showed that only 17 samples (18%) were *Daphne giraldii* Nitsche (DG), which is recorded in Chinese Pharmacopeia, while the others (82%) might have safety hazards. Additionally, the result of HPLC fingerprint analysis indicated that samples in the same species origins and wild distributions could be clustered together, which was consistent with DNA barcoding analysis. The study can provide a significant system for the authentication and quality of commercial Cortex Daphnes herbs. Undoubtedly, this study undoubtedly confirmed that the chemical compositions of Cortex Daphnes herbs were affected by both species origins and ecological environments, which is required more in-depth research.

## Introduction

Cortex Daphnes (Cirald Daphne Bark), a kind of traditional Chinese medicine, is the dried root bark and stem bark of *DG* recorded in the Chinese Pharmacopeia. However, many *Daphne* Linn plants, such as *Daphne gracilis* E. Pritz., *Daphne limprichtii* H. Winkl. and *Edgeworthia* Meisn plants, such as *Edgeworthia chrysantha* Lindl., etc., are also used as Cortex Daphnes in some parts of China [1–4]. *Daphne tangutica* Maxim (*DT*) and *Daphne retusa* Hemsl (*DR*) are just contained in some provincial medicinal material codes [5–6]. The *Daphne Linn* comprises approximately 95 species found in the alpine regions of Asia and Europe. About 44 species are found in China, and mainly distributed in southwest and northwest of China. The three *Daphne* species mostly is located in Provinces of Gansu, Shaanxi, Qinghai and Sichuan [7].

As a traditional Chinese medicine, Cortex Daphnes is pungent, bitter in taste, warm in nature, and of a little toxicity. Acting on the liver channel, the medical efficacy of Cortex Daphnes is dispelling wind and eliminating dampness, promoting blood circulation to remove blood stasis and scattered stasis pain [8]. Phytochemical studies showed that there are mainly four active classes of constituents in the dried velamen and bark of Daphnes Cortex, including coumarins (e.g. daphnetoxin), flavonoids (e.g. luteolin), lignans (e.g. syringaresinol), triterpenes (e.g. oleanolic acid), it also contains syringin, daucosterol, etc. Daphnetin is not the characteristic constituents but also the major bioactive constituents of them. Modern pharmacological studies have shown that Cortex Daphnes extract exhibits various efficacy, such as anti-inflammation, analgesia, anti-malaria, anti-tumor, antithrombus, anti-fertility. Daphnetin effectively inhibit tumor cell growth and kill the malaria parasite, and also its extract has remarkable therapeutic effect on glomerulonephritis [9–12]. In recent pharmaceutical market, the dosage forms of Cortex Daphnes are mainly injection, tablets and patch, which are used as the Extra-Strength Pain Reliever and largely used for the treatment of rheumatic arthralgia, arthralgia, traumatic injury, arthritis, rheumatoid arthritis and promoting blood circulation, and also can cure some blood vessel diseases, such as cardiovascular and cerebrovascular diseases, especially for thrombosis angitis obliterans.

Cortex Daphnes and its relative products is greatly in the world. Up to the year of 2015 in the survey areas, the wild resources storage of Cortex Daphnes was only 583.62t, the storage of *DG* was 97.64t (16.7%), the storage of *DT* was 392.66t (67.3%), the storage of *DR* was 93.32t (16%), according to the resources survey results from our research group (Beijing University of Chinese Medicine, Beijing, China). Many *Daphne* plants are used as Cortex Daphnes in folk of China and ordinary people is unable to correctly identify them well. Few *Daphne* plants have been cultivated and almost all wild materials are obtained from the agricultural markets in different areas[13–14].

The medicinal plant trade is the primary source of income for herbalists, and economic constraints may provide incentives for herbalists to substitute cheaper and more readily available species for rare ingredients and sell them under the same name [15]. The constituents contained in medicinal materials of Cortex Daphnes herbs collected from different habitats and different species origins are varied, which may directly influence the quality and efficacy of corresponding Chinese patent medicine.

Morphological classification is an important method for the identification of medicinal plants, mostly depending on their leaves, flowers and fruits. The harvest time of Cortex Daphnes herbs is in March of each year. However, there is no flower or fruit in March when villagers collect Cortex Daphnes herbs, so bark of the three *Daphne* species as Cortex Daphnes herbs circulated in the medicinal markets is more difficult to be identified because of the high similarity of their external characteristics [3]. Therefore, more scientific and accurate identification methods are required. Currently, DNA barcoding is recognized as a technology that is able to accurately and efficiently identify the species of medicinal plants. Internal transcribed spacer of ribosome gene (ITS) sequence is able to provide sufficient information for species identification [16–21].

Generally, the curative effects of traditional Chinese medicine (TCM) are the results of multiple bioactive components. Chromatographic fingerprinting provide an entire profile of almost global component of herbal medicines and is considered to be an important method for evaluating the quality of herbal medicines. It has been internationally accepted by the World Health Organization (WHO), the Food and Drug Administration of the USA (FDA), the Chinese State Food and Drug Administration (SFDA) and other authorities. HPLC fingerprinting is the most widely used method for qualitative evaluation and species identification of herbal medicines due to its convenience and efficiency [22–25].

In China, 93 wild Cortex Daphnes samples in different species were collected from different provinces covered the main wild distribution areas. Therefore, the aim of this study was to develop a valid and accurate system based on DNA barcoding and HPLC fingerprint methods, to identify and classify the species origins and habitats of the 93 Cortex Daphnes samples and control quality. Standard compound daphnetin was used as reference component for the qualitative of chromatographic peak by an HPLC- UV method. Samples are compared visually and analyzed using neighbor-joining tree (N-J tree) analysis, similarity analysis (SA), hierarchical cluster analysis (HCA) and principal component analysis (PCA). This study will be helpful in development of strategies for conservation, utilization and quality control of Cortex Daphnes and other herbs. The system could be used by the supervisory departments for the market supervision of commercial Cortex Daphnes herbs in the future, and provide a identification model for other commercial Chinese herbal medicines. More importantly, the classification results can preliminarily lay a foundation for the research that how species origins and ecological environments effect on the chemical components of Cortex Daphnes herbs.

## Results

### Main characteristics on morphology and habitat of the three *Daphne* species

According to the previous resources survey results, the plant morphology (S1 File) of *DG* is different from the other two *Daphne* species (*DT* and *DR*). The plant morphology of *DT* and *DR* is quite similar to each other. The flowers of *DG* are yellow and its leaves are pale green and membranous. However, the flowers of *DT* and *DR* are all fuchsia and the leaves of them are dark green and leathery. It is easy to separate *DG* from *DT* and *DR* by comparing the characteristics of their flowers and leaves in the flowering and fruiting stages. The leaves of *DT* are long, narrow and lanceolate. The leaves of *DR* are long ovate, and the front-end of leaves are concave down, which is able to be selected as a major characteristic to identify *DT* and *DR*. It shows that the genetic relationship between *DT* and *DR* is most similar to each other, which is consistent with records about *DR* in *flora of China* [26]. However, the identification of plants by the external form is restricted by collection time before blooming and growing environment, and the success of differentiation depending on the difference of the subtle external morphological features is restricted by professional experience.

**Fig 1:**
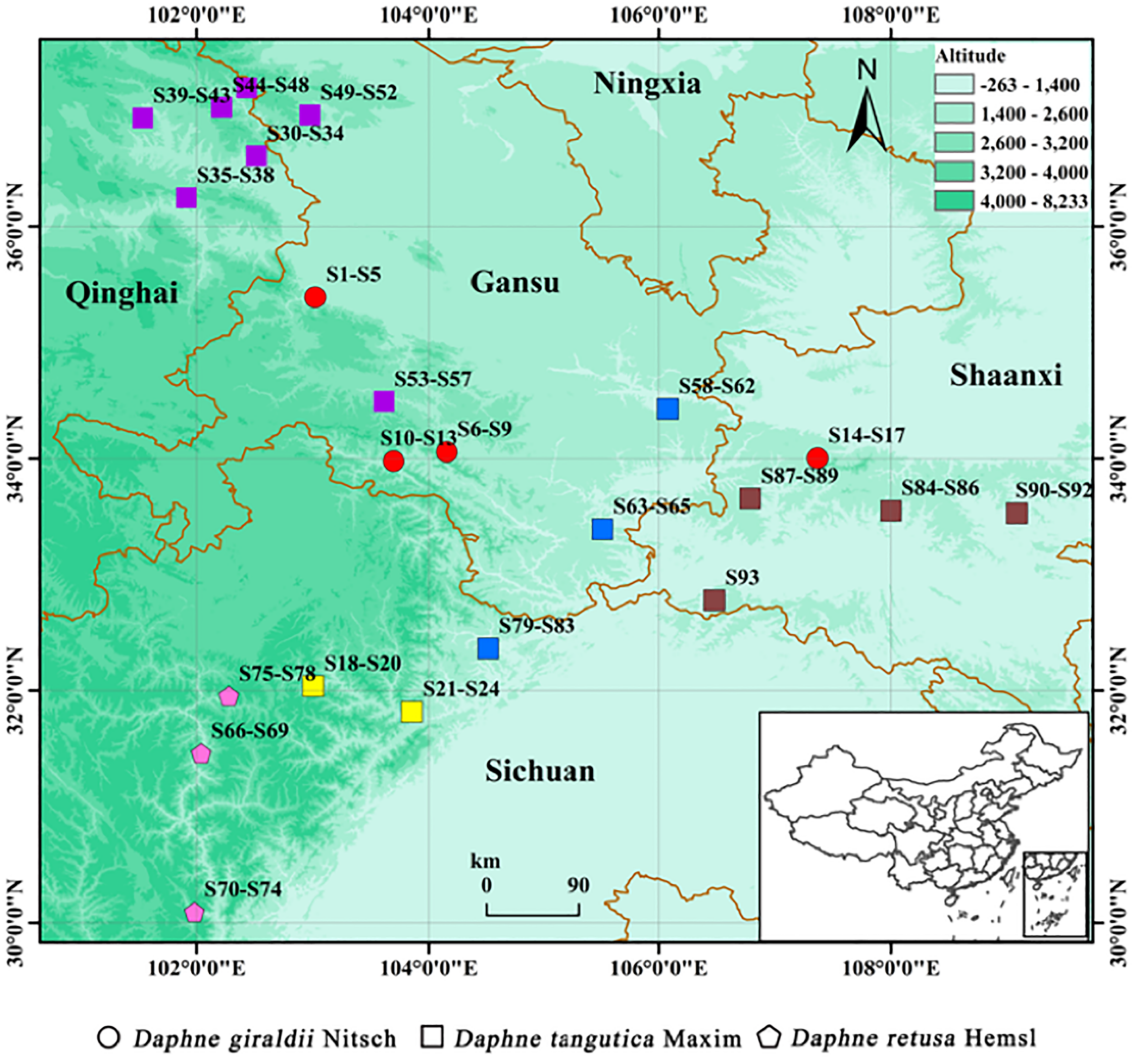
Distributions of the 93 collected Cortex Daphnes samples. This map was generated by the software ESRI ArcGIS Desktop, version: 10.3.0.4322, URL http://www.esri.com/. It was created by author L. G. (The border line is vectorized China map data.).

There are obvious differences in habitats of the three *Daphne* species. *DG* often grows under the bushes, where the community mostly consists of shrubs and small trees. The soil types are mostly humus with good permeability. The air humidity is relatively modest. Theoretically, the habitat altitude range of *DG* is 1600∼2600 m, however, we only found them where the altitude range is 2186∼2554 m in the resources survey. *DT* often grows in the sparse forest, alpine grassland and forest edge, especially on the sunny slope, with the altitude range 1000∼3800 m. The coenotype is relatively diverse. According to *Meteorological Data Center of China Meteorological Administration* [27], there are large differences in the climate type of each *DT* habitat, which is able to be divided into four groups. Group 1: the climate types of habitats including Menyuan, Ledu, Hualong, Datong and Zhuoni counties are all plateau continental climate; the climate types of Huzhu and Tianzhu counties are continental cold temperate climate and plateau continental monsoon climate. Characteristics of these climate types in group 1 are more similar. Group 2: the climate types of habitats including Heishui and Mao counties are all plateau monsoon climate. Group 3: the climate types of habitats including Foping, Liuba and Ningqiang counties are all warm temperate humid monsoon climate, the climate type of habitat Zhenan is semi-humid climate. Characteristics of these climate types are relatively mild and more similar. Group 4: the climate types of habitats including Tianshui and Pingwu are temperate monsoon climate and subtropical mountain humid monsoon climate. Kang counties is the excessive area that subtropical transition to warm temperate zone. Characteristics of climate types in group 4 are mild, abundant rainfall and more similar. *DR* often grows on alpine grass slope, with the altitude range 3000∼3900 m. The climate types of habitats including Jinchuan, Kangding and Maerkang counties are continental plateau climate, alpine plateau climate and low latitude, high altitude special geography and alpine canyon three-dimensional climate. These characteristics of climate types are a bit similar to the plateau monsoon climate. For the same *Daphne* species, habitat may be the major factor, which result in their chemical composition and gene segment exist differences. The specific habitat distributions of the 93 Cortex Daphnes samples are shown in Fig 1 (generated by the software ESRI ArcGIS Desktop, version: 10.3.0.4322, URL http://www.esri.com/).

### Genetic taxonomy of Cortex Daphnes samples

All of the collected samples were identified according to their ITS sequences. To identify the species of the 93 Cortex Daphnes samples more accurately and visually, a phylogenetic tree was constructed based on the ITS sequences (S2 File) obtained from GenBank and the samples. Likewise, the modified ITS sequences were submitted to DNAMAN software to compare similarities of the samples. Among them, 76 Cortex Daphnes samples of *DT* and *DR* were identified with the similarities higher than 99%, and the other 17 Cortex Daphnes samples of *DG* were also identified. The similarity of ITS sequences between *DG* and *DT* was 93% and the similarity between *DG* and *DR* was 92%. However, the similarity between *DT* and *DR* was 99%. The genotype of ITS sequences in *DT* at 60bp and 156bp were C and G. But, the genotype of ITS sequences in *DR* at the same locus were all A, based on this, it also able to identify them accurately.

**Fig 2:**
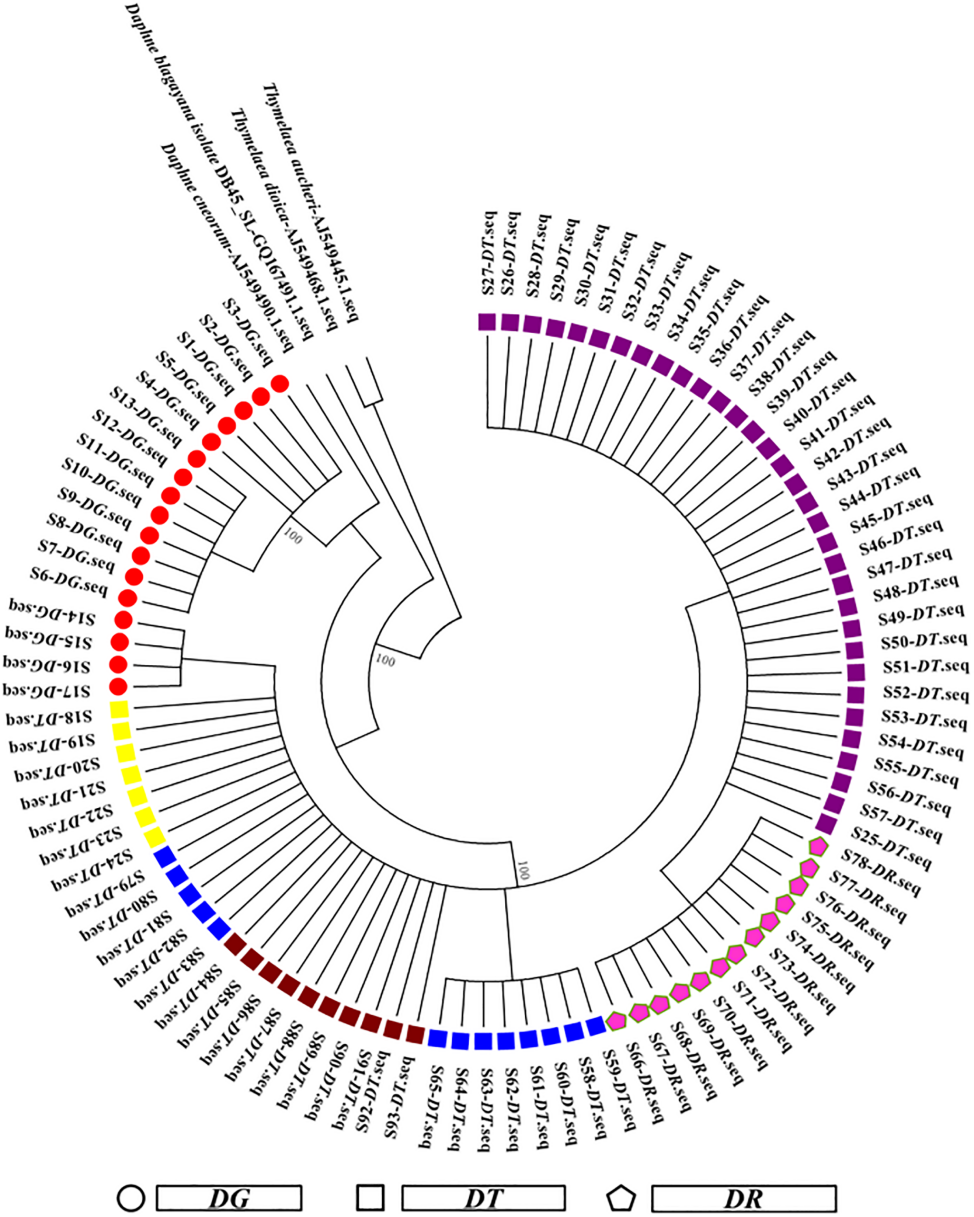
Results of NJ-tree analysis based on ITS sequences of the 93 Cortex Daphnes samples.

HCA based on ITS sequences was used to analyze the genetic data to characterize the population genetics of the Cortex Daphnes samples and determine their genetic diversity and population differentiation. *Thymelaea aucheri* (AJ549445.1), *Daphne cneorum* (AJ549490.1), *Thymelaea dioica* (AJ549468.1) and *Daphne blagayana isolate* DB45_SL (GQ167491.1) downloaded from GenBank-NCBI were selected as out-group gene sequences to obtain more accurate branching of the phylogenetic tree. The wild Cortex Daphnes populations distributed in western China were grouped separately through their ITS sequences (as shown in Fig 2).

All ITS sequences of Cortex Daphnes samples were merged with *Daphne blagayana isolate* DB45_SL (GQ167491.1), which indicated that these samples belonged to *Daphne Linn*. All samples were divided into two main characteristic clusters. The first cluster consisted of *DG*. The other cluster was made up with *DT* and *DR* which was divided into five main branches. The first branch was consisted of *DT* and contained 33 samples (S25–S48, collected from Qinghai Province; S49–S57 collected from Tianzhu and Zhuoni counties in Gansu Province, the altitude of habitats higher than 2900 m). The second branch including *DR* contained 13 samples (S66–S78, all gathered from Sichuan Province). The first branch merged with the second branch (*DR*) to form a larger branch that merged with the other branches. The third branch was consisted of *DT* and included 8 samples (S58–S65, collected from Tianshui and Kang counties in Gansu Province). The fourth branch was consisted of *DT* and included 22 samples (S84–S93, all gathered from Shaanxi Province; S79–S83, S18–S24, gathered from Pingwu, Mao and Heishui counties in Sichuan Province). According to plant morphological, the fifth branch contained 4 samples (S14–S17, gathered from Taibai county in Shaanxi Province) was identified to be *DG*. However, it was merged with *DT* and *DR* cluster. Likewise, it was also close to the *DG* cluster. That may be the species variation of *DG* caused by specific ecological environment or the coenospecies of *DG* and *DT*. The NJ tree obtained from ITS sequences is able to easily distinguish and identify the 93 Daphnes Cortex samples gathered from different species origins and habitats. The results of HCA were in good agreement with the results of the morphological taxonomy. HCA placed all *DT* samples into one of two main branches. *DT* samples with the altitude of habitats more than 2900 m were grouped in one branch, and the others with the altitude of habitats below 2900 m merged gradually became one branch. It likely showed that altitude of habitats may have important influence on the ITS sequences of Cortex Daphnes. Accordingly, it revealed that samples from the same species origins, similar geographical environments and nearby regions had similar ITS sequences and were in the same or close clusters. DNA barcoding could successfully identify the Cortex Daphnes samples.

### Method validation of HPLC fingerprint

#### Optimization of extraction and chromatographic conditions

Four different concentrations of methanol and ethanol (60, 70, 80, and 100%) extractions were compared. The best extracting way was ultrasonic and extracted for 45 min in 70% methanol. Under such condition, more chromatographic peaks and more chemical contents were detected. As a result, the best extracting condition was established as follows: the samples were extracted by ultrasonic extraction using 70% methanol as the extracted solvent and the duration was 45min.

To obtain more chromatographic peaks, the optimized gradient elution program was used in this study. To make the chromatograms with better separation and sharper peaks, the mobile phase, column temperature and detection wavelength were all optimized. The elution effect of the mobile phase constitution (methanol / water, methanol / 0.5% FA (formic acid) water, acetonitrile / water, acetonitrile / 0.5% FA water) on the chromatographic separation was compared. It showed that acetonitrile / 0.5% FA water was the best mobile phase composition with higher elution efficiency. In this study, three column temperatures (20, 25, 30°C) were selected to assess their efficiency on gaining higher resolution of the chromatographic peaks. As a result, the best column temperature was 25°C. The wavelength for the detection of compounds was selected by UV detector. Most of the chromatographic peaks could be detected at approximately 327 nm, at which the chromatograms could provide maximum absorption of daphnetin. So 327nm were chosen as detection wavelength for the HPLC fingerprint analysis.

#### Precision, repeatability and stability tests

The test of precision, repeatability and stability were performed by calculating the relative standard deviations (RSDs) of relative retention times (RRTs) and relative peak areas (RPAs) based on common peaks respectively. The results were showed in Table 1. All RSDs including RRTs and RPAs were < 3%, which indicated that the method was sufficiently accurate, stabilized and sensitive for the fingerprint analysis of the 93 Cortex Daphnes samples.

**Table 1:** Test results of precision, repeatability and stability for the ten common peaks.

Peak No.	Precision (n = 6)		Repeatability (n = 6)		Stability (n = 6)	
	RSD of RRT%	RSD of RPA%	RSD of RRT %	RSD of RPA%	RSD of RRT %	RSD of RPA%
1	0.038	1.982	0.045	2.805	0.032	0.377
2	0.039	1.405	0.028	2.027	0.034	1.484
3	0.002	0.246	0.048	2.512	0.052	0.732
4	0.000	0.433	0.044	2.387	0.061	1.895
5	0.017	0.293	0.047	2.736	0.063	2.553
7	0.039	1.970	0.055	1.580	0.058	2.123
9	0.049	1.083	0.898	1.813	0.016	0.787
11	0.077	1.572	0.044	0.944	0.021	0.849
23	0.068	1.751	0.006	2.199	0.025	0.515
29	0.078	1.124	0.097	2.352	0.031	1.280

#### Establishment of chromatographic fingerprint of Cortex Daphnes samples

**Fig 3:**
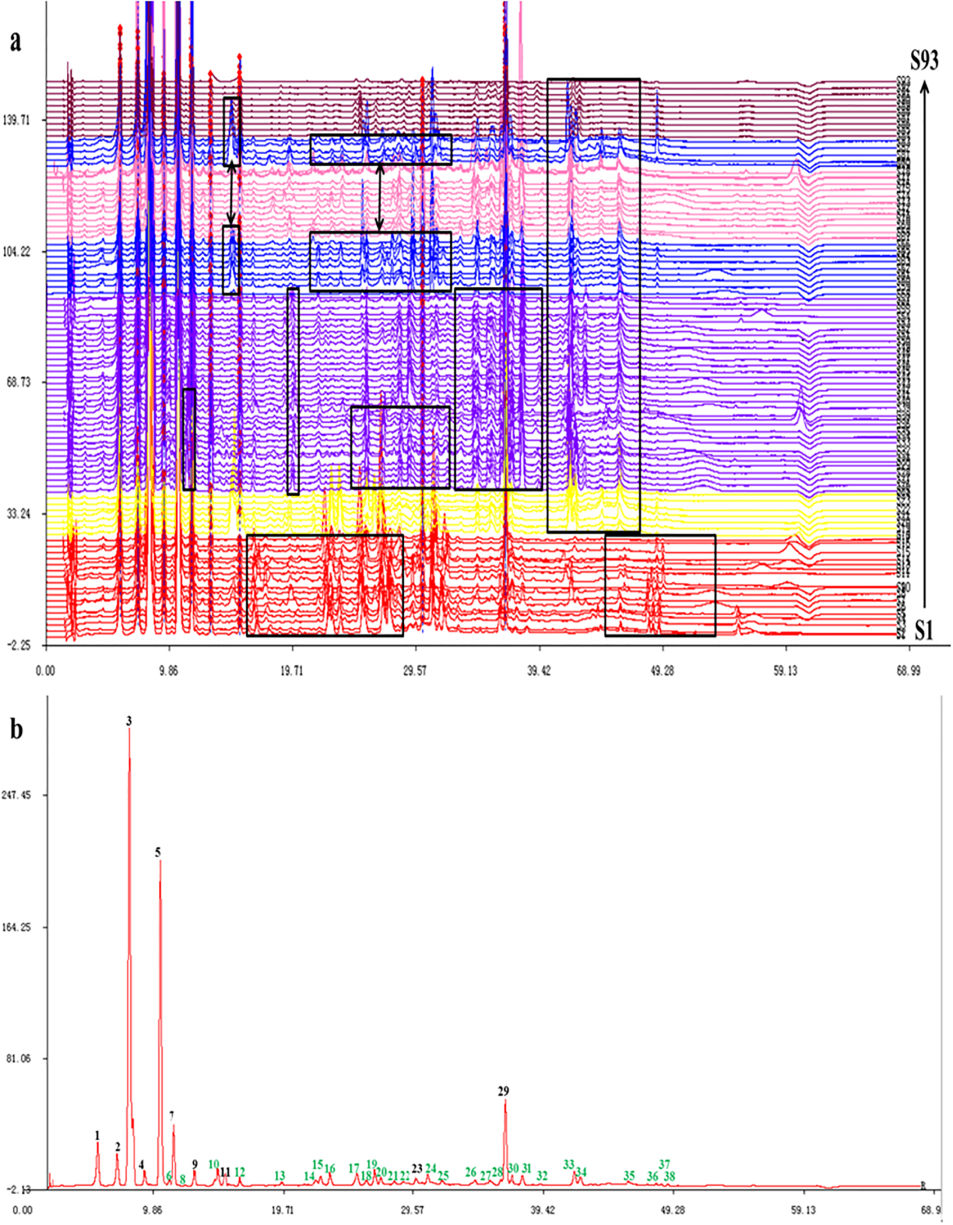
(a) HPLC fingerprints of the 93 Cortex Daphnes samples (S1-S93) and (b) reference chromatogram. 4: Syringoside 5: Daphnetin 9: 7-hydroxycoumarin.

To establish the chromatographic fingerprint, 93 Cortex Daphnes samples from different species and habitats were analyzed under the optimized chromatographic analysis conditions. Sample 1 was the reference sample, and all chromatograms (as shown in Fig 3a) through multipoint correction and free matching were matched and reference chromatogram (as shown in Fig 3b) was generated by the *computer-aided Similarity Evaluation System for Chromatographic Fingerprint of TCM* (Version 2004A). Peaks in the fingerprint with quite large area and good resolution shared by all the chromatograms of the tested samples were selected as “common characteristic peaks” to represent the characteristics of all the samples. A total of 10 common peaks shared by all samples (black peak No.) which covered more than 90% of the total area and 28 common peaks detected in portion samples (green peak No.) were determined in the reference chromatogram. One component was identified as daphnetin (peak 5) by comparing its retention time and UV spectrum with the standard compound. The other common fingerprint peaks were not identified.

### SA of HPLC fingerprint of Cortex Daphnes samples

The similarity values between 93 Cortex Daphnes samples and the reference chromatogram were calculated using the *Similarity Evaluation System for Chromatographic Fingerprint of TCM* (Version 2004A), and the results were presented in Table 2.

**Table 2:** Results of similarity evaluation between samples and reference chromatogram.

Sample	SC	Sample	SC	Sample	SC	Sample	SC	Sample	SC
S1	0.975	S21	0.945	S41	0.993	S61	0.947	S81	0.982
S2	0.973	S22	0.923	S42	0.991	S62	0.974	S82	0.961
S3	0.97	S23	0.946	S43	0.99	S63	0.934	S83	0.955
S4	0.975	S24	0.943	S44	0.994	S64	0.98	S84	0.947
S5	0.976	S25	0.98	S45	0.994	S65	0.951	S85	0.945
S6	0.991	S26	0.987	S46	0.982	S66	0.947	S86	0.925
S7	0.979	S27	0.99	S47	0.974	S67	0.978	S87	0.931
S8	0.985	S28	0.989	S48	0.992	S68	0.988	S88	0.985
S9	0.954	S29	0.981	S49	0.989	S69	0.978	S89	0.989
S10	0.983	S30	0.969	S50	0.994	S70	0.991	S90	0.981
S11	0.98	S31	0.971	S51	0.99	S71	0.956	S91	0.968
S12	0.976	S32	0.922	S52	0.995	S72	0.955	S92	0.974
S13	0.983	S33	0.915	S53	0.994	S73	0.922	S93	0.977
S14	0.982	S34	0.909	S54	0.958	S74	0.966		
S15	0.963	S35	0.98	S55	0.956	S75	0.95		
S16	0.959	S36	0.961	S56	0.992	S76	0.995		
S17	0.956	S37	0.928	S57	0.986	S77	0.974		
S18	0.98	S38	0.933	S58	0.977	S78	0.941		
S19	0.984	S39	0.954	S59	0.93	S79	0.989		
S20	0.983	S40	0.996	S60	0.978	S80	0.971		

According to the results, the similarity values were all >0.9. It was indicated that the main chemical compositions among the 93 Cortex Daphnes samples were relatively consistent. However, there were still many differences of chemical compositions among the Cortex Daphnes samples gathered from different habitats and species origins. According to the fingerprint chromatograms, some characteristic peaks especially in the range of 12–55 min only appeared in part of the samples collected from similar ecological environment or the same species origins. These special common characteristic peaks were also marked with green peak No. in the reference chromatogram. Obviously, the types and quantities of chemical components among Cortex Daphnes samples were not completely consistent. The varied chemical profiles of Cortex Daphnes samples may be attributed to the different species origins and habitats, which were the results of plants adapting to the environment.

### HCA of HPLC fingerprint of Cortex Daphnes samples

**Fig 4:**
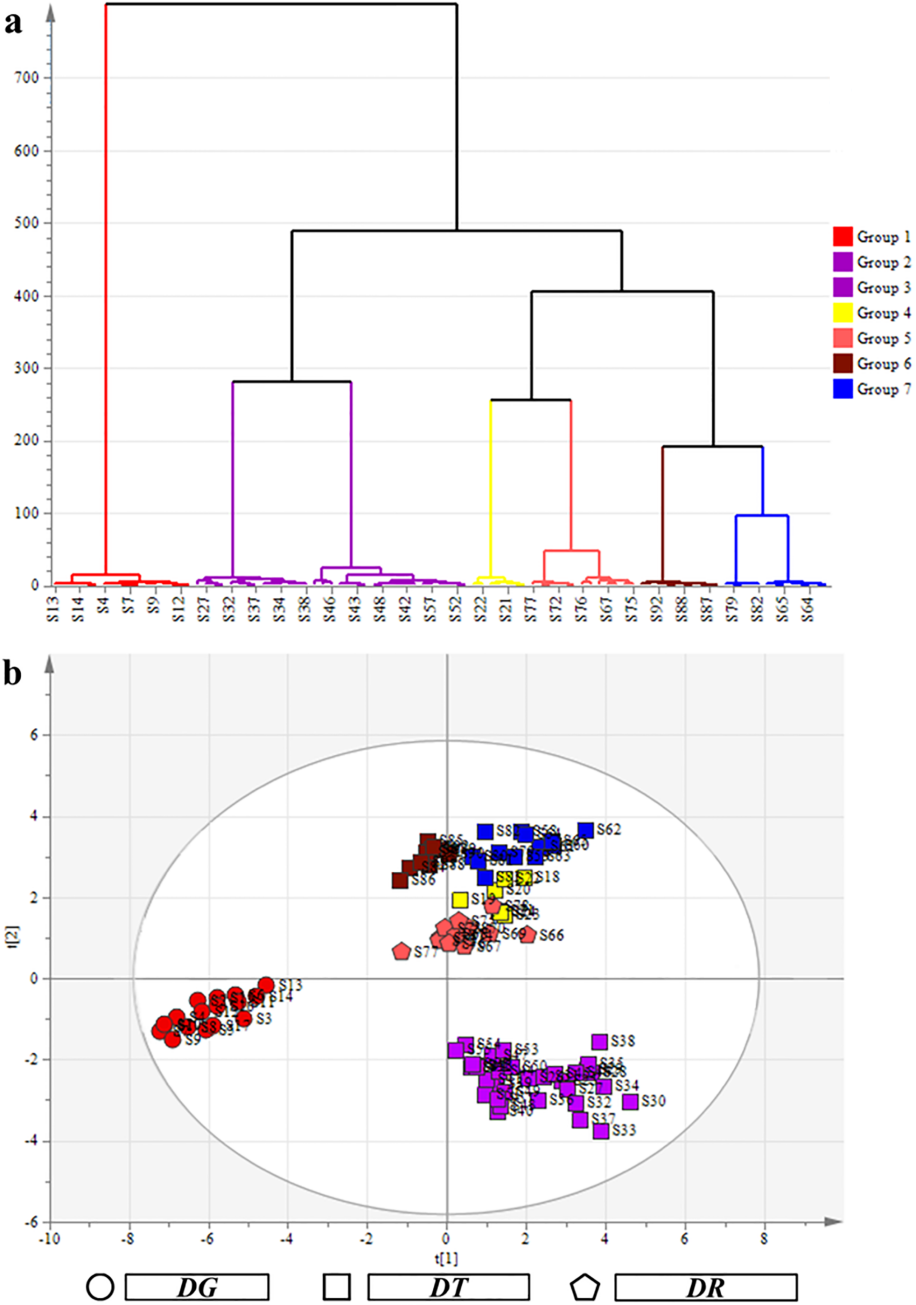
(a) Results of HCA based on HPLC fingerprint of the 93 Cortex Daphnes samples and (b) score plot of PCA of the 93 Cortex Daphnes samples.

HCA, one of the chemical pattern recognition and classification evaluation methods, is used to set the level of bottom-up decomposition for a given data set until certain conditions are fulfilled. HCA has been commonly applied for fingerprint analysis with standard normal variant transformation of the data, which led to meaningful classification of herbal samples collected from different regions [28–29]. In order to show the degree of similarity and differences among the 93 Cortex Daphnes samples more clearly, the HCA in this study was performed based on the RPAs (S3 File) of all common characteristic peaks (peaks 1–38, as shown in Fig 3b) by the professional analysis software *SIMCA 13*.*0 Demo*. The results of HCA were shown in Fig 4a.

According to the results, all samples were classified into two main clusters: S1–S17 in cluster 1, S18–93 in cluster 2 (as shown in Fig 4a). Cluster 1 including 17 samples belonged to *DG*. Additionally, cluster 2 was divided into six groups: S25–S57 in group1and group 2, S18–S24 in group 3, S66–S78 in group 4, S84–S93 in group 5, S58–S65 and S79–S83 in group 6. These five groups including 63 samples belonged to *DT*. Group 4 including 13 samples belonged to *DR* (all gathered from Sichuan Province). Group 1 and group 2 including 33 samples were collected from Qinghai and Gansu Province with the altitude of habitats more than 2900 m. Group 3 including 7 samples were gathered from two adjacent counties (Maoxian and Heishui counties in Sichuan Province). Group 5 including 10 samples were all gathered from Shaanxi province. Group 6 including 13 samples were gathered from Sichuan and Gansu Province. The Group 3 (*DT*) merged with the Group 4 (*DR*) to form a larger branch1. Maybe it’s because they were gathered from nearby areas. According to the HCA of chemical components and N-J tree of ITS sequences, the *DR* samples merged with the *DT* samples consistently which indicated that the chemical compositions and the ITS sequences between the two species were much of a muchness. The results were consistent with the recordation about *DR* and *DT* in *Flora of China* [26]. The Group 5 (*DT*) merged with the Group 6 (*DT*) to form a larger branch 2. All samples in the branch 2 were gathered from Qinling and Dabashan mountain areas where the ecological environments were more similar. The branch 1 and branch 2 merged to form a larger branch in which the altitude of the sample collection sites were below 2900 m then merged with group 1and group 2. The classification results of HCA were consistent with the classification of climate types of habitats, and agreed well with the categorized results of the genetic taxonomy and the visual comparisons of their representative chromatograms, which may provide more references for further quality control and evaluation of the commercial Cortex Daphnes herbs.

### PCA of HPLC fingerprint of Cortex Daphnes samples

PCA is a multivariate method and widely used in data analysis to summarize variation, which is implemented as a data-reduction technique to generate a visual scatter plot for the qualitative evaluation of resemblances and differences between the studied samples [22]. In order to differentiate all the Cortex Daphnes samples clearly, the PCA was carried out based on the RPAs (S3 File) of all common characteristic peaks (peaks 1–38, as shown in Fig 3b) by the professional analysis software *SIMCA 13*.*0 Demo*. The score plot was structured based on the first three principal components which accounted for more than 89.31% of the total variability, and the other principal components which had little effect on the model were discarded.

According to the results of PCA, all samples were divided into seven groups according to their different sources (as shown in Fig 4b). Group 1 contained 17 samples (S1-S17) belonging to *DG*. Group 2 and group 3 contained 33 samples (S25-S57) belonging to *DT*. Group 4 contained 13 samples (S66-S78) belonging to *DR*. Group 5 contained 7 samples (S17-S24) belonging to *DT*. Group 6 contained 10 samples (S84-S93) belonging to *DT*. Group 7 contained 13 samples (S58-S65, S79-S83) belonging to *DT*. Groups excepting group 1, group 2 and group 3 were quite close to each other. The results of PCA were in good agreement with the results of HCA based on the fingerprint. The classification results of the scatter plot adequately showed the noticeable provenance and geographical differences among the samples.

In this study, samples (S14-S17) were divided into *DG* groups according to the results of phytochemical and morphological taxonomy which was not completely consistent with results of the genetic taxonomic. This conclusion illustrated that the accurate identification of medicinal materials requires comprehensive applications of multifarious identification techniques.

## Discussion

In this study, a system based on DNA barcoding and HPLC fingerprint analysis to identify and classify the Cortex Daphnes herbs has been established. Two universal classification techniques include four different analysis methods: SA, HCA and PCA of the chemical components and the NJ-tree analysis of ITS sequences. 93 Cortex Daphnes samples, gathered from different species origins and wild areas in Western China, were identified and classified successfully. The results of genetic taxonomic were greatly consistent with the phytochemical taxonomy results. The morphological taxonomy also played an important role in the identification of Cortex Daphnes samples, such as the distinction of species *DR*. The conclusions drawn from the classification system were more objective and scientific. The classification system could efficiently identify and control the quality of the three *Daphne* species. Furthermore, these methods are convenient and suitable for practical use, further pharmacological research and development of the three species. The system is also able to provide a identification model for other commercial Chinese herbal medicines to guarantee their clinical safety.

The results of genetic and phytochemical taxonomic showed that samples belonging to the same species origins and nearby habitats could be clustered together, which demonstrated that the type and composition of the chemical components of the medicinal plants were the combined effect of both genetic materials and habitats. For the same *Daphne* species, climate types of their habitats may be the decisive factor that make their chemical compositions exist larger differences. The altitude may play an important role, which may be as a classification foundation (as shown in Fig 1). In addition to the altitude, there are many other ecological factors which can influence the type and composition of the chemical components of the medicinal plants, such as temperature, soil, light, moisture and so on. In order to guarantee the quality stability of the Cortex Daphnes herbs, to realize the artificial cultivation and protect wild plants, the effects exerted by the genetic materials and ecological factors to the active ingredients of the three *Daphne* species is required deep study in future.

## Materials and methods

### Chemicals and materials

Methanol (analytical grade) and acetonitrile (HPLC grade) were purchased from Fisher Scientific International (Fair Lawn, New Jersey, USA). Ultra-pure water was generated by an Ultrapure Water System (Shanghai Ultrapure Technology, Shanghai, China). Standard daphnetin was purchased from the National Institutes for Food and Drug Control (Beijing, China). The 93 wild Cortex Daphnes samples including 93 stem bark coupled with 93 leaves were collected from four local provinces of China: Gansu, Shaanxi, Qinghai and Sichuan. They were identified as genuine samples of *DG*, *DT* and *DR* by Professor Chunsheng Liu (Beijing University of Chinese Medicine, Beijing, China) through their leaves (as listed in Table 3 [14]). All samples were dried at 22–25°C. The 93 stem bark were comminuted to powder separately, and sieved through a 74μm (or 200 mesh) screen for HPLC analysis and the leaves were used for DNA barcoding analysis.

**Table 3:** Sources of the 93 Cortex Daphnes samples.

Sample No.	Location	Latitude	Longitude	Altitude	Origin
		N	E	m	
S1-S5	Linxia, Gansu	35.389217	103.022983	2554	*Daphne giraldii* Nitsch
S6-S9	Tanchang, Gansu	34.056550	104.156800	2186	*Daphne giraldii* Nitsch
S10-S13	Diebu, Gansu	33.977450	103.698217	2461	*Daphne giraldii* Nitsch
S14-S17	Taibai, Shaanxi	34.002633	107.365500	2518	*Daphne giraldii* Nitsch
S18-S20	Heishui, Sichuan	32.039883	103.005500	2975	*Daphne tangutica* Maxim
S21-S24	Maoxian, Sichuan	31.818083	103.855400	2000	*Daphne tangutica* Maxim
S25-S29	Menyuan, Qinghai	37.193783	102.432317	2985	*Daphne tangutica* Maxim
S30-S34	Ledu, Qinghai	36.606717	102.515783	3032	*Daphne tangutica* Maxim
S35-S38	Hualong, Qinghai	36.247400	101.912950	3002	*Daphne tangutica* Maxim
S39-S43	Datong, Qinghai	36.934333	101.536500	3063	*Daphne tangutica* Maxim
S44-S48	Huzhu, Qinghai	37.025333	102.215733	3293	*Daphne tangutica* Maxim
S49-S52	Tianzhu, Gansu	36.959967	102.977033	2834	*Daphne tangutica* Maxim
S53-S57	Zhuoni, Gansu	34.491300	103.615283	3315	*Daphne tangutica* Maxim
S58-S62	Tianshui, Gansu	34.427583	106.075817	1468	*Daphne tangutica* Maxim
S63-S65	Kangxian, Gansu	33.391550	105.514017	1524	*Daphne tangutica* Maxim
S66-S69	Jinchuan, Sichuan	31.459533	102.040417	3108	*Daphne retusa* Hemsl
S70-S74	Kangding, Sichuan	30.093317	101.981867	3900	*Daphne retusa* Hemsl
S75-S78	Maerkang, Sichuan	31.954117	102.277333	3076	*Daphne retusa* Hemsl
S79-S83	Pingwu, Sichuan	32.363217	104.514000	1675	*Daphne tangutica* Maxim
S84-S86	Foping, Shaanxi	33.550000	108.000000	1584	*Daphne tangutica* Maxim
S87-S89	Liuba, Shaanxi	33.654680	106.787400	1502	*Daphne tangutica* Maxim
S90-S92	Zhenan, Shaanxi	33.528200	109.085100	1866	*Daphne tangutica* Maxim
S93	Ningqiang, Shaanxi	32.779200	106.477300	1617	*Daphne tangutica* Maxim

### DNA barcoding analysis

#### DNA extraction

Genomic DNA from dried leaves of 93 Cortex Daphnes samples which were collected in Beijing University of Chinese Medicine were extracted according to the instructions of the plant DNA extraction kit (Tiangen, Beijing, China). All the DNA were stored at -20°C before analysis [30].

#### PCR and sequencing

A total of 30μL PCR system contains: template DNA 3μL, Mix-Taq enzyme 15μL, ITS sense primer 1.2μL, ITS antisense primer 1.2μL, ddH_2_O 9.6μL. PCR amplification was performed: 95°C for 4 min, followed by 35 cycles of 94°C for 30s, 55°C for 1 min, 72°C for 1 min, and final extension 72°C for 10min (Bio-Rad T100^™^ Thermal Cycler). 5μL of PCR products were examined via electrophoresis in a 1.0% agarose gel, and were sequenced by Shanghai sangong company. To ensure the accuracy, samples were all sequenced in two-way. All the ITS sequences obtained were cut and spliced using *DNAMAN* and *ContigExpress* software, then were submitted to GenBank-NCBI for comparison with the deposited sequences of near-source species using the tool BLAST. The modified ITS sequences were aligned and a N-J tree was constructed based on standard parameters with bootstrap testing of 1000 replicates using *ClustalX and MEGA version 5*.*0* software. The N-J tree was used to observe the natural interrelationships and differences for each of the Cortex Daphnes samples by ITS sequences [31].

### HPLC analysis

#### Samples and standard solution preparation

The dried powdered sample (0.500g) was precisely weighed and extracted with 50 mL of 70% v/v methanol by ultrasonic extraction for 45 min. The extraction solution was supplemented to the weight of pre-extracted before analysis when it cooled down to room temperature. The solution was filtered through a 0.45 μm membrane filter and stored at 4°C out of light before HPLC analysis.

The standard daphnetin was prepared for the qualitative of chromatographic peak. The concentration of standard solution was 60μg/mL in methanol and 10μL solution was injected into the HPLC for analysis according to the standard [6]. The standard solution was filtered through 0.45 μm membrane filters and stored at 4°C out of light and brought to room temperature before HPLC analysis.

#### Instrumentation and analytical conditions

HPLC analyses were performed using a Waters system (Waters e2695/2489/Emp2) with a UV detector. Chromatographic separations were achieved using gradient elution on a C_18_ reserve-phase column (4.6 mm× 150 mm, 5 μm; Agilent Technologies). The column temperature was maintained at 25°C. The absorption wavelength was 327 nm which was selected by UV detector according to max UV absorption of the reference. The mobile phase consisted of acetonitrile (A) and 0.5% FA water (B) with a linear gradient elution at a flow rate of 1.0 mL/min. The gradient elution program was as follows: 5–27.4% (A) in 0–40 min, 27.4–42%(A) in 40–45 min, 42–53% (A) in 45–58 min, 53–90% (A) in 58–68 min and 90–5% (A) in 68–78 min. The sample injection volume was 10μL.

The daphnetin as the common components of the three species which chromatographic peak was selected to be the referential chromatographic peak, the RRTs and RPAs were analyzed. Method precision was determined by injecting one Cortex Daphnes sample solution (S1) six times continuously. The repeatability was assessed through six independently prepared sample solutions (S1). The stability of the injection solution was determined periodically by injecting samples (S1) stored at 4°C ranging from 0 to 24 h (0, 3, 6, 9, 12, and 24h). The results were all expressed by RSDs of RRTs and RPAs, respectively, of all common peaks (1, 2, 3, 4, 5, 7, 9, 11, 23 and 29) from six chromatographic profiles of Cortex Daphnes sample (S1).

### Data analysis

#### SA analysis

The HPLC analyses of all the samples were carried out under the established experimental condition. The data of all chromatographic profiles was converted into “AIA” form from the software of Empower. SA was performed on the basis of the RRTs and RPAs using the professional software named *Similarity Evaluation System for Chromatographic Fingerprint of TCM* (2004A), which was recommended by the State Food and Drug Administration of China (SFDA) for calculating the similarity coefficient (SC) of the chromatographic profiles of TCM. The similarities among different chromatograms were quantified by calculating the correlation coefficient or the cosine value of the vectorial angle [32–33].

#### HCA and PCA analysis

The statistical analysis was performed using the professional analysis software *SIMCA 13*.*0 Demo* for HCA and PCA. HCA and PCA were used to show the unsupervised clustering pattern of the *Daphne* Linn species and discover the differences in samples caused by complex factors. HCA and PCA were used to discover the natural interrelationships among the chemical components for each of the Cortex Daphnes samples [29]. The RPAs of main characteristic peaks were selected as the clustering variable and the critical *P* value for all analyses in this study was set to 0.05.

All the data were pretreated including background deduction and the chromatograms alignment before SA, HCA, and PCA. The datasets generated and analyzed during the current study are available from the corresponding author on reasonable request.

## Supporting information

S1 File Photos of three Daphne species. (PDF)

S2 File ITS sequences obtained from samples and GenBank. (PDF)

S3 File Results of HCA and PCA of Cortex Daphnes samples. (PDF)
