# Supplementary material for: Identification of three Daphne species by DNA barcoding and HPLC fingerprint analysis
Source: PLoS One. 2018 Aug 2;13(8):e0201711. doi: 10.1371/journal.pone.0201711 (PMC6072044; doi:10.1371/journal.pone.0201711)

S1 File. Photos of three *Daphne* species

Daphne giraldii Nitsche (DG)

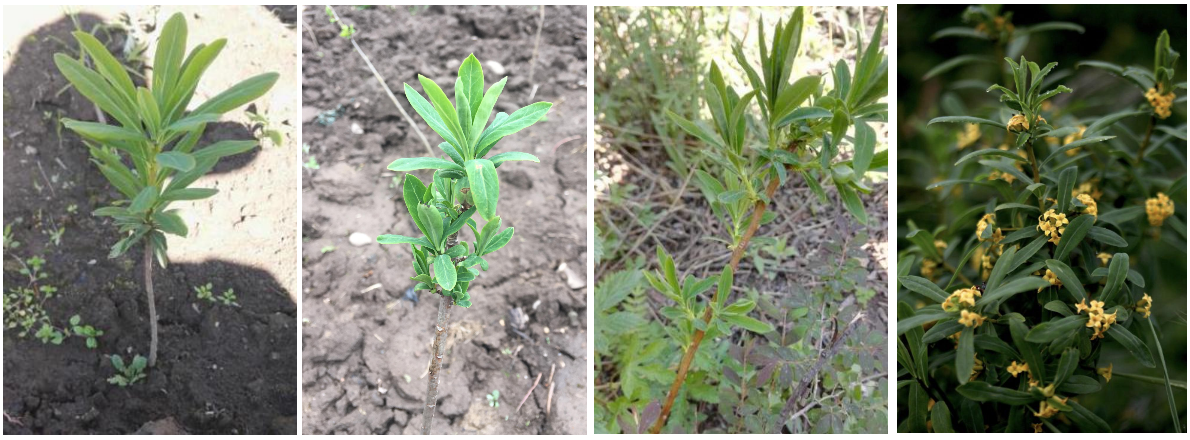

Daphne tangutica Maxim (*DT*)

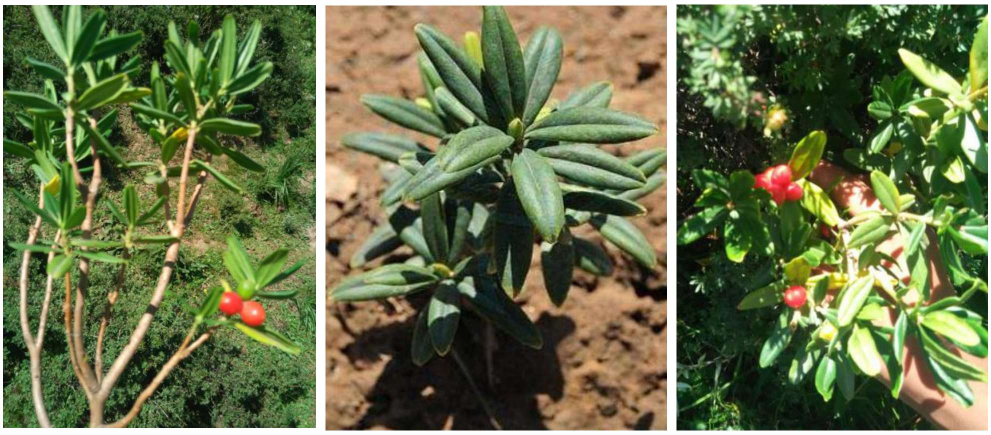

Daphne retusa Hemsl (*DR*)

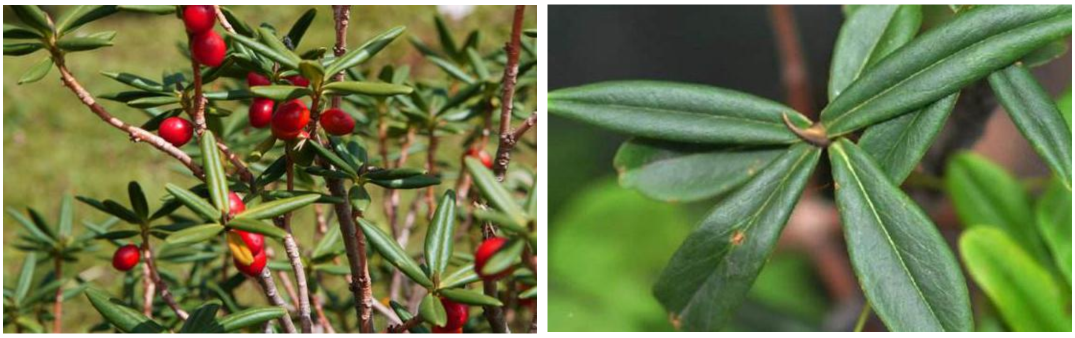

Supplement: S1 File — (PDF) [file pone.0201711.s001.pdf]
