# Supplementary material for: Identification of three Daphne species by DNA barcoding and HPLC fingerprint analysis
Source: PLoS One. 2018 Aug 2;13(8):e0201711. doi: 10.1371/journal.pone.0201711 (PMC6072044; doi:10.1371/journal.pone.0201711)
Supplement: S2 File — (PDF) [file pone.0201711.s002.pdf]

S2 File. ITS sequences obtained from samples and GenBank

S1-*DG*:

AATGCATAAAATATCTTCATCCAAAGGTTTGAAGTACAAGTGCAACCAACTCTAATGAC
ATGCCATACACGATGACTTAACGAGGGCAAGCCAAGACAAACCACCGCTCATCATGAA
ATACATGACATTAATGATTACCCTATTTCAGCCAACCGCACTACTATGAGAACGGGAGA
GCCATATAAGCCCTACACCAACAAATGTTGTAGAGGCTACAATGCGTGACACCCAAGC
AGACGTGCCCTCAGCCCGAAGGCTTGGGGCGCAACTTGCGTTCAAAGACTCGATGGT
TCACGGGATTCTGCAATTCACACCAAGTATCGCATTTCGCTACGTTCTTCATCGATGCA
AGAGCCGAGATATCCGTTGCCGAGAGTCATTCTTTATAATACATATCACGTATTACCGCA
CACAATCTTATGCATGGGGAAAACGTTTCATTTATCACAATTCCTTGGCACAATCCGCG
CCGGGGTTGGTTATCTTGGCCACATGGGCCAGTCGAGAGGAGATGGAGCCGATGACAC
CAAAGCCCCCCTCAACACATTTGCAAACATGTTCACAGGTTGTTTTGCTTTGCAGGATT
CGA

S2-*DG*:

AATGCATAAAATATCTTCATCCAAAGGTTTGAAGTACAAGTGCAACCAACTCTAATGAC
ATGCCATACACGATGACTTAACGAGGGCAAGCCAAGACAAACCACCGCTCATCATGAA
ATACATGACATTAATGATTACCCTATTTCAGCCAACCGCACTACTATGAGAACGGGAGA
GCCATATAAGCCCTACACCAACAAATGTTGTAGAGGCTACAATGCGTGACACCCAAGC
AGACGTGCCCTCAGCCCGAAGGCTTGGGGCGCAACTTGCGTTCAAAGACTCGATGGT
TCACGGGATTCTGCAATTCACACCAAGTATCGCATTTCGCTACGTTCTTCATCGATGCA
AGAGCCGAGATATCCGTTGCCGAGAGTCATTCTTTATAATACATATCACGTATTACCGCA
CACAATCTTATGCATGGGGAAAACGTTTCATTTATCACAATTCCTTGGCACAATCCGCG
CCGGGGTTGGTTATCTTGGCCACATGGGCCAGTCGAGAGGAGATGGAGCCGATGACAC
CAAAGCCCCCCTCAACACATTTGCAAACATGTTCACAGGTTGTTTTGCTTTGCAGGATT
CGA

S3-*DG*:

AATGCATAAAATATCTTCATCCAAAGGTTTGAAGTACAAGTGCAACCAACTCTAATGAC
ATGCCATACACGATGACTTAACGAGGGCAAGCCAAGACAAACCACCGCTCATCATGAA
ATACATGACATTAATGATTACCCTATTTCAGCCAACCGCACTACTATGAGAACGGGAGA
GCCATATAAGCCCTACACCAACAAATGTTGTAGAGGCTACAATGCGTGACACCCAAGC
AGACGTGCCCTCAGCCCGAAGGCTTGGGGCGCAACTTGCGTTCAAAGACTCGATGGT
TCACGGGATTCTGCAATTCACACCAAGTATCGCATTTCGCTACGTTCTTCATCGATGCA
AGAGCCGAGATATCCGTTGCCGAGAGTCATTCTTTATAATACATATCACGTATTACCGCA
CACAATCTTATGCATGGGGAAAACGTTTCATTTATCACAATTCCTTGGCACAATCCGCG
CCGGGGTTGGTTATCTTGGCCACATGGGCCAGTCGAGAGGAGATGGAGCCGATGACAC
CAAAGCCCCCCTCAACACATTTGCAAACATGTTCACAGGTTGTTTTGCTTTGCAGGATT
CGA

S4-*DG*:

AATGCATAAAATATCTTCATCCAAAGGTTTGAAGTACAAGTGCAACCAACTCTAATGAC
ATGCCATACACGATGACTTAACGAGGGCAAGCCAAGACAAACCACCGCTCATCATGAA
ATACATGACATTAATGATTACCCTATTTCAGCCAACCGCACTACTATGAGAACGGGAGA
GCCATATAAGCCCTACACCAACAAATGTTGTAGAGGCTACAATGCGTGACACCCAAGC
AGACGTGCCCTCAGCCCGAAGGCTTGGGGCGCAACTTGCGTTCAAAGACTCGATGGT
TCACGGGATTCTGCAATTCACACCAAGTATCGCATTTCGCTACGTTCTTCATCGATGCA

AGAGCCGAGATATCCGTTGCCGAGAGTCATTCTTTATAATACATATCACGTATTACCGCA
CACAATCTTATGCATGGGGAAAACGTTTCATTTATCACAATTCCTTGGCACAATCCGCG
CCGGGGTTGGTTATCTTGGCCACATGGGCCAGTCGAGAGGAGATGGAGCCGATGACAC
CAAAGCCCCCCTCAACACATTTGCAAACATGTTCACAGGTTGTTTTGCTTTGCAGGATT
CGA

S5-*DG*:

AATGCATAAAATATCTTCATCCAAAGGTTTGAAGTACAAGTGCAACCAACTCTAATGAC
ATGCCATACACGATGACTTAACGAGGGCAAGCCAAGACAAACCACCGCTCATCATGAA
ATACATGACATTAATGATTACCCTATTTCAGCCAACCGCACTACTATGAGAACGGGAGA
GCCATATAAGCCCTACACCAACAAATGTTGTAGAGGCTACAATGCGTGACACCCAAGC
AGACGTGCCCTCAGCCCGAAGGCTTGGGGCGCAACTTGCGTTCAAAGACTCGATGGT
TCACGGGATTCTGCAATTCACACCAAGTATCGCATTTCGCTACGTTCTTCATCGATGCA
AGAGCCGAGATATCCGTTGCCGAGAGTCATTCTTTATAATACATATCACGTATTACCGCA
CACAATCTTATGCATGGGGAAAACGTTTCATTTATCACAATTCCTTGGCACAATCCGCG
CCGGGGTTGGTTATCTTGGCCACATGGGCCAGTCGAGAGGAGATGGAGCCGATGACAC
CAAAGCCCCCCTCAACACATTTGCAAACATGTTCACAGGTTGTTTTGCTTTGCAGGATT
CGA

S6-*DG*:

AATGCATAAAATATCTTCATCCAAAGGTTTGAAGTACAAGTGCAACCAACTCTAATGAC
ATGCCATACACGATGACTTAACGAGGGCAAGCCAAGACAAACCACCGCTCATCATGAA
ATACATGACATTAATGATTACCCTATTTCAGCCAACCGCACTACTATGAGAACGGGAGA
GCCATATAAGCCCTACACAAACAAATGTTGTAGAGGCTACAATGCGTGACACCCAAGC
AGACGTGCCCTCAGCCCGAAGGCTTGGGGCGCAACTTGCGTTCAAAGACTCGATGGT
TCACGGGATTCTGCAATTCACACCAAGTATCGCATTTCGCTACGTTCTTCATCGATGCA
AGAGCCGAGATATCCGTTGCCGAGAGTCATTCTTTATAATACATATCACGTATTACCGCA
CACAATCTTATGCATGGGGAAAACGTTTCATTTATCACAATTCCTTGGCACAATCCGCG
CCGGGGTTGGTTATCTTGGCCACATGGGCCAGTCGAGAGGAGATGGAGCCGATCACAC
CAAAGCCCCCCTCAACACATTTGCAAACATGTTCACAGGTTGTTTTGCTTTGCAGGATT
CGA

S7-*DG*:

AATGCATAAAATATCTTCATCCAAAGGTTTGAAGTACAAGTGCAACCAACTCTAATGAC
ATGCCATACACGATGACTTAACGAGGGCAAGCCAAGACAAACCACCGCTCATCATGAA
ATACATGACATTAATGATTACCCTATTTCAGCCAACCGCACTACTATGAGAACGGGAGA
GCCATATAAGCCCTACACAAACAAATGTTGTAGAGGCTACAATGCGTGACACCCAAGC
AGACGTGCCCTCAGCCCGAAGGCTTGGGGCGCAACTTGCGTTCAAAGACTCGATGGT
TCACGGGATTCTGCAATTCACACCAAGTATCGCATTTCGCTACGTTCTTCATCGATGCA
AGAGCCGAGATATCCGTTGCCGAGAGTCATTCTTTATAATACATATCACGTATTACCGCA
CACAATCTTATGCATGGGGAAAACGTTTCATTTATCACAATTCCTTGGCACAATCCGCG
CCGGGGTTGGTTATCTTGGCCACATGGGCCAGTCGAGAGGAGATGGAGCCGATCACAC
CAAAGCCCCCCTCAACACATTTGCAAACATGTTCACAGGTTGTTTTGCTTTGCAGGATT
CGA

S8-*DG*:

AATGCATAAAATATCTTCATCCAAAGGTTTGAAGTACAAGTGCAACCAACTCTAATGAC
ATGCCATACACGATGACTTAACGAGGGCAAGCCAAGACAAACCACCGCTCATCATGAA

ATACATGACATTAATGATTACCCTATTTCAGCCAACCGCACTACTATGAGAACGGGAGA
GCCATATAAGCCCTACACAAACAAATGTTGTAGAGGCTACAATGCGTGACACCCAAGC
AGACGTGCCCTCAGCCCGAAGGCTTGGGGCGCAACTTGCGTTCAAAGACTCGATGGT
TCACGGGATTCTGCAATTCACACCAAGTATCGCATTTCGCTACGTTCTTCATCGATGCA
AGAGCCGAGATATCCGTTGCCGAGAGTCATTCTTTATAATACATATCACGTATTACCGCA
CACAATCTTATGCATGGGGAAAACGTTTCATTTATCACAATTCCTTGGCACAATCCGCG
CCGGGGTTGGTTATCTTGGCCACATGGGCCAGTCGAGAGGAGATGGAGCCGATCACAC
CAAAGCCCCCCTCAACACATTTGCAAACATGTTCACAGGTTGTTTTGCTTTGCAGGATT
CGA

S9-*DG*:

AATGCATAAAATATCTTCATCCAAAGGTTTGAAGTACAAGTGCAACCAACTCTAATGAC
ATGCCATACACGATGACTTAACGAGGGCAAGCCAAGACAAACCACCGCTCATCATGAA
ATACATGACATTAATGATTACCCTATTTCAGCCAACCGCACTACTATGAGAACGGGAGA
GCCATATAAGCCCTACACAAACAAATGTTGTAGAGGCTACAATGCGTGACACCCAAGC
AGACGTGCCCTCAGCCCGAAGGCTTGGGGCGCAACTTGCGTTCAAAGACTCGATGGT
TCACGGGATTCTGCAATTCACACCAAGTATCGCATTTCGCTACGTTCTTCATCGATGCA
AGAGCCGAGATATCCGTTGCCGAGAGTCATTCTTTATAATACATATCACGTATTACCGCA
CACAATCTTATGCATGGGGAAAACGTTTCATTTATCACAATTCCTTGGCACAATCCGCG
CCGGGGTTGGTTATCTTGGCCACATGGGCCAGTCGAGAGGAGATGGAGCCGATCACAC
CAAAGCCCCCCTCAACACATTTGCAAACATGTTCACAGGTTGTTTTGCTTTGCAGGATT
CGA

S10-*DG*:

AATGCATAAAATATCTTCATCCAAAGGTTTGAAGTACAAGTGCAACCAACTCTAATGAC
ATGCCATACACGATGACTTAACGAGGGCAAGCCAAGACAAACCACCGCTCATCATGAA
ATACATGACATTAATGATTACCCTATTTCAGCCAACCGCACTACTATGAGAACGGGAGA
GCCATATAAGCCCTACACAAACAAATGTTGTAGAGGCTACAATGCGTGACACCCAAGC
AGACGTGCCCTCAGCCCGAAGGCTTGGGGCGCAACTTGCGTTCAAAGACTCGATGGT
TCACGGGATTCTGCAATTCACACCAAGTATCGCATTTCGCTACGTTCTTCATCGATGCA
AGAGCCGAGATATCCGTTGCCGAGAGTCATTCTTTATAATACATATCACGTATTACCGCA
CACAATCTTATGCATGGGGAAAACGTTTCATTTATCACAATTCCTTGGCACAATCCGCG
CCGGGGTTGGTTATCTTGGCCACATGGGCCAGTCGAGAGGAGATGGAGCCGATCACAC
CAAAGCCCCCCTCAACACATTTGCAAACATGTTCACAGGTTGTTTTGCTTTGCAGGATT
CGA

S11-*DG*:

AATGCATAAAATATCTTCATCCAAAGGTTTGAAGTACAAGTGCAACCAACTCTAATGAC
ATGCCATACACGATGACTTAACGAGGGCAAGCCAAGACAAACCACCGCTCATCATGAA
ATACATGACATTAATGATTACCCTATTTCAGCCAACCGCACTACTATGAGAACGGGAGA
GCCATATAAGCCCTACACAAACAAATGTTGTAGAGGCTACAATGCGTGACACCCAAGC
AGACGTGCCCTCAGCCCGAAGGCTTGGGGCGCAACTTGCGTTCAAAGACTCGATGGT
TCACGGGATTCTGCAATTCACACCAAGTATCGCATTTCGCTACGTTCTTCATCGATGCA
AGAGCCGAGATATCCGTTGCCGAGAGTCATTCTTTATAATACATATCACGTATTACCGCA
CACAATCTTATGCATGGGGAAAACGTTTCATTTATCACAATTCCTTGGCACAATCCGCG
CCGGGGTTGGTTATCTTGGCCACATGGGCCAGTCGAGAGGAGATGGAGCCGATCACAC
CAAAGCCCCCCTCAACACATTTGCAAACATGTTCACAGGTTGTTTTGCTTTGCAGGATT

CGA

S12-*DG*:

AATGCATAAAATATCTTCATCCAAAGGTTTGAAGTACAAGTGCAACCAACTCTAATGAC
ATGCCATACACGATGACTTAACGAGGGCAAGCCAAGACAAACCACCGCTCATCATGAA
ATACATGACATTAATGATTACCCTATTTCAGCCAACCGCACTACTATGAGAACGGGAGA
GCCATATAAGCCCTACACAAACAAATGTTGTAGAGGCTACAATGCGTGACACCCAAGC
AGACGTGCCCTCAGCCCGAAGGCTTGGGGCGCAACTTGCGTTCAAAGACTCGATGGT
TCACGGGATTCTGCAATTCACACCAAGTATCGCATTTCGCTACGTTCTTCATCGATGCA
AGAGCCGAGATATCCGTTGCCGAGAGTCATTCTTTATAATACATATCACGTATTACCGCA
CACAATCTTATGCATGGGGAAAACGTTTCATTTATCACAATTCCTTGGCACAATCCGCG
CCGGGGTTGGTTATCTTGGCCACATGGGCCAGTCGAGAGGAGATGGAGCCGATCACAC
CAAAGCCCCCCTCAACACATTTGCAAACATGTTCACAGGTTGTTTTGCTTTGCAGGATT
CGA

S13-*DG*:

AATGCATAAAATATCTTCATCCAAAGGTTTGAAGTACAAGTGCAACCAACTCTAATGAC
ATGCCATACACGATGACTTAACGAGGGCAAGCCAAGACAAACCACCGCTCATCATGAA
ATACATGACATTAATGATTACCCTATTTCAGCCAACCGCACTACTATGAGAACGGGAGA
GCCATATAAGCCCTACACAAACAAATGTTGTAGAGGCTACAATGCGTGACACCCAAGC
AGACGTGCCCTCAGCCCGAAGGCTTGGGGCGCAACTTGCGTTCAAAGACTCGATGGT
TCACGGGATTCTGCAATTCACACCAAGTATCGCATTTCGCTACGTTCTTCATCGATGCA
AGAGCCGAGATATCCGTTGCCGAGAGTCATTCTTTATAATACATATCACGTATTACCGCA
CACAATCTTATGCATGGGGAAAACGTTTCATTTATCACAATTCCTTGGCACAATCCGCG
CCGGGGTTGGTTATCTTGGCCACATGGGCCAGTCGAGAGGAGATGGAGCCGATCACAC
CAAAGCCCCCCTCAACACATTTGCAAACATGTTCACAGGTTGTTTTGCTTTGCAGGATT
CGA

S14-*DG*:

AATGCGTACTATTATCTTCATCCAAAGGTTTGCAGTACAGATGCAACCAACTCTAATGA
CATGCCACATGATGACTTAACGAGGGCAAGCCAAGGCAAACCACCGCTCATCATGAAA
TACATGACATTAATGGTTACTCTATTTCAACCAACCGCACTACTATGAGTACGGGAGAG
CCATATCAGTCCTACACCAATTACTGTTGTAGAGCCTACAATGCGTGACACCCAGGCAG
ACGTGCCCTCGACCAAAAGGCTTGGGGCGCAACTTGCGTTCAAAGACTCGATGGTTC
ACGGGATTCTGCAATTCACACCAAGTATCGCATTTCGCTACGTTCTTCATCGATGCAAG
AGCCGAGATATCCGTTGCCGAGAGTCATTATTTATAATACATATCACGTATTACCCCACA
CAATCTTATGTTAGATAGATGGGAAAACGTTTCATTTATCACAATTCCTTGGCACAATCC
GCGCCGGGGTTGGTTATCTTGGCCACATGGGCCAGTCGAGAGGAGATGGAGCAAATGA
CACCAAGCCCCCTCAACACATTTGTAAACATGTTCACAGGTTGTTCTGCTTTGCAGGAT
TCGA

S15-*DG*:

AATGCGTACTATTATCTTCATCCAAAGGTTTGCAGTACAGATGCAACCAACTCTAATGA
CATGCCACATGATGACTTAACGAGGGCAAGCCAAGGCAAACCACCGCTCATCATGAAA
TACATGACATTAATGGTTACTCTATTTCAACCAACCGCACTACTATGAGTACGGGAGAG
CCATATCAGTCCTACACCAATTACTGTTGTAGAGCCTACAATGCGTGACACCCAGGCAG
ACGTGCCCTCGACCAAAAGGCTTGGGGCGCAACTTGCGTTCAAAGACTCGATGGTTC
ACGGGATTCTGCAATTCACACCAAGTATCGCATTTCGCTACGTTCTTCATCGATGCAAG

AGCCGAGATATCCGTTGCCGAGAGTCATTATTTATAATACATATCACGTATTACCCCACA
CAATCTTATGTTAGATAGATGGGAAAACGTTTCATTTATCACAATTCCTTGGCACAATCC
GCGCCGGGGTTGGTTATCTTGGCCACATGGGCCAGTCGAGAGGAGATGGAGCAAATGA
CACCAAGCCCCCTCAACACATTTGTAAACATGTTCACAGGTTGTTCTGCTTTGCAGGAT
TCGA

S16-*DG*:

AATGCGTACTATTATCTTCATCCAAAGGTTTGCAGTACAGATGCAACCAACTCTAATGA
CATGCCACATGATGACTTAACGAGGGCAAGCCAAGGCAAACCACCGCTCATCATGAAA
TACATGACATTAATGGTTACTCTATTTCAACCAACCGCACTACTATGAGTACGGGAGAG
CCATATCAGTCCTACACCAATTACTGTTGTAGAGCCTACAATGCGTGACACCCAGGCAG
ACGTGCCCTCGACCAAAAGGCTTGGGGCGCAACTTGCGTTCAAAGACTCGATGGTTC
ACGGGATTCTGCAATTCACACCAAGTATCGCATTTCGCTACGTTCTTCATCGATGCAAG
AGCCGAGATATCCGTTGCCGAGAGTCATTATTTATAATACATATCACGTATTACCCCACA
CAATCTTATGTTAGATAGATGGGAAAACGTTTCATTTATCACAATTCCTTGGCACAATCC
GCGCCGGGGTTGGTTATCTTGGCCACATGGGCCAGTCGAGAGGAGATGGAGCAAATGA
CACCAAGCCCCCTCAACACATTTGTAAACATGTTCACAGGTTGTTCTGCTTTGCAGGAT
TCGA

S17-*DG*:

AATGCGTACTATTATCTTCATCCAAAGGTTTGCAGTACAGATGCAACCAACTCTAATGA
CATGCCACATGATGACTTAACGAGGGCAAGCCAAGGCAAACCACCGCTCATCATGAAA
TACATGACATTAATGGTTACTCTATTTCAACCAACCGCACTACTATGAGTACGGGAGAG
CCATATCAGTCCTACACCAATTACTGTTGTAGAGCCTACAATGCGTGACACCCAGGCAG
ACGTGCCCTCGACCAAAAGGCTTGGGGCGCAACTTGCGTTCAAAGACTCGATGGTTC
ACGGGATTCTGCAATTCACACCAAGTATCGCATTTCGCTACGTTCTTCATCGATGCAAG
AGCCGAGATATCCGTTGCCGAGAGTCATTATTTATAATACATATCACGTATTACCCCACA
CAATCTTATGTTAGATAGATGGGAAAACGTTTCATTTATCACAATTCCTTGGCACAATCC
GCGCCGGGGTTGGTTATCTTGGCCACATGGGCCAGTCGAGAGGAGATGGAGCAAATGA
CACCAAGCCCCCTCAACACATTTGTAAACATGTTCACAGGTTGTTCTGCTTTGCAGGAT
TCGA

S18-*DT*:

AATGCGTACTATTATCTTCATCCAAAGGTTTGCAGTACAGATGCAACCAACTCTAATGA
CATGCCACATGATGACTTAACGAGGGCAAGCCAAGGCAAACCACCGCTCATCATGAAA
TACATGACATTAATGGTTACTCTATTTCAACCAACCGCACTACTATGAGTACGGGAGAG
CCATATCAGTCCTACACCAATTACTGTTGTAGAGCCTACAATGCGTGACACCCAGGCAG
ACGTGCCCTCGACCAAAAGGCTTGGGGCGCAACTTGCGTTCAAAGACTCGATGGTTC
ACGGGATTCTGCAATTCACACCAAGTATCGCATTTCGCTACGTTCTTCATCGATGCAAG
AGCCGAGATATCCGTTGCCGAGAGTCATTCTTTATAATACATATCACGTATTACCCCACA
CAATCTTATGTTAGATAGATGGGAAAACGTTTCATTTATCACAATTCCTTGGCACAATCC
GCGCCGGGGTTGGTTATCTTGGCCACATGGGCCAGTCGAGAGGAGATGAAGCAAATGA
CACCAAGCCCCCTCAACACATTTGTAAACATGTTCACAGGTTGTTCTGCTTTGCAGGAT
TCGA

S19-*DT*:

AATGCGTACTATTATCTTCATCCAAAGGTTTGCAGTACAGATGCAACCAACTCTAATGA
CATGCCACATGATGACTTAACGAGGGCAAGCCAAGGCAAACCACCGCTCATCATGAAA

TACATGACATTAATGGTTACTCTATTTCAACCAACCGCACTACTATGAGTACGGGAGAG
CCATATCAGTCCTACACCAATTACTGTTGTAGAGCCTACAATGCGTGACACCCAGGCAG
ACGTGCCCTCGACCAAAAGGCTTGGGGCGCAACTTGCGTTCAAAGACTCGATGGTTC
ACGGGATTCTGCAATTCACACCAAGTATCGCATTTCGCTACGTTCTTCATCGATGCAAG
AGCCGAGATATCCGTTGCCGAGAGTCATTCTTTATAATACATATCACGTATTACCCCACA
CAATCTTATGTTAGATAGATGGGAAAACGTTTCATTTATCACAATTCCTTGGCACAATCC
GCGCCGGGGTTGGTTATCTTGGCCACATGGGCCAGTCGAGAGGAGATGAAGCAAATGA
CACCAAGCCCCCTCAACACATTTGTAAACATGTTCACAGGTTGTTCTGCTTTGCAGGAT
TCGA

S20-*DT*:

AATGCGTACTATTATCTTCATCCAAAGGTTTGCAGTACAGATGCAACCAACTCTAATGA
CATGCCACATGATGACTTAACGAGGGCAAGCCAAGGCAAACCACCGCTCATCATGAAA
TACATGACATTAATGGTTACTCTATTTCAACCAACCGCACTACTATGAGTACGGGAGAG
CCATATCAGTCCTACACCAATTACTGTTGTAGAGCCTACAATGCGTGACACCCAGGCAG
ACGTGCCCTCGACCAAAAGGCTTGGGGCGCAACTTGCGTTCAAAGACTCGATGGTTC
ACGGGATTCTGCAATTCACACCAAGTATCGCATTTCGCTACGTTCTTCATCGATGCAAG
AGCCGAGATATCCGTTGCCGAGAGTCATTCTTTATAATACATATCACGTATTACCCCACA
CAATCTTATGTTAGATAGATGGGAAAACGTTTCATTTATCACAATTCCTTGGCACAATCC
GCGCCGGGGTTGGTTATCTTGGCCACATGGGCCAGTCGAGAGGAGATGAAGCAAATGA
CACCAAGCCCCCTCAACACATTTGTAAACATGTTCACAGGTTGTTCTGCTTTGCAGGAT
TCGA

S21-*DT*:

AATGCGTACTATTATCTTCATCCAAAGGTTTGCAGTACAGATGCAACCAACTCTAATGA
CATGCCACATGATGACTTAACGAGGGCAAGCCAAGGCAAACCACCGCTCATCATGAAA
TACATGACATTAATGGTTACTCTATTTCAACCAACCGCACTACTATGAGTACGGGAGAG
CCATATCAGTCCTACACCAATTACTGTTGTAGAGCCTACAATGCGTGACACCCAGGCAG
ACGTGCCCTCGACCAAAAGGCTTGGGGCGCAACTTGCGTTCAAAGACTCGATGGTTC
ACGGGATTCTGCAATTCACACCAAGTATCGCATTTCGCTACGTTCTTCATCGATGCAAG
AGCCGAGATATCCGTTGCCGAGAGTCATTCTTTATAATACATATCACGTATTACCCCACA
CAATCTTATGTTAGATAGATGGGAAAACGTTTCATTTATCACAATTCCTTGGCACAATCC
GCGCCGGGGTTGGTTATCTTGGCCACATGGGCCAGTCGAGAGGAGATGAAGCAAATGA
CACCAAGCCCCCTCAACACATTTGTAAACATGTTCACAGGTTGTTCTGCTTTGCAGGAT
TCGA

S22-*DT*:

AATGCGTACTATTATCTTCATCCAAAGGTTTGCAGTACAGATGCAACCAACTCTAATGA
CATGCCACATGATGACTTAACGAGGGCAAGCCAAGGCAAACCACCGCTCATCATGAAA
TACATGACATTAATGGTTACTCTATTTCAACCAACCGCACTACTATGAGTACGGGAGAG
CCATATCAGTCCTACACCAATTACTGTTGTAGAGCCTACAATGCGTGACACCCAGGCAG
ACGTGCCCTCGACCAAAAGGCTTGGGGCGCAACTTGCGTTCAAAGACTCGATGGTTC
ACGGGATTCTGCAATTCACACCAAGTATCGCATTTCGCTACGTTCTTCATCGATGCAAG
AGCCGAGATATCCGTTGCCGAGAGTCATTCTTTATAATACATATCACGTATTACCCCACA
CAATCTTATGTTAGATAGATGGGAAAACGTTTCATTTATCACAATTCCTTGGCACAATCC
GCGCCGGGGTTGGTTATCTTGGCCACATGGGCCAGTCGAGAGGAGATGAAGCAAATGA
CACCAAGCCCCCTCAACACATTTGTAAACATGTTCACAGGTTGTTCTGCTTTGCAGGAT

TCGA

S23-*DT*:

AATGCGTACTATTATCTTCATCCAAAGGTTTGCAGTACAGATGCAACCAACTCTAATGA
CATGCCACATGATGACTTAACGAGGGCAAGCCAAGGCAAACCACCGCTCATCATGAAA
TACATGACATTAATGGTTACTCTATTTCAACCAACCGCACTACTATGAGTACGGGAGAG
CCATATCAGTCCTACACCAATTACTGTTGTAGAGCCTACAATGCGTGACACCCAGGCAG
ACGTGCCCTCGACCAAAAGGCTTGGGGCGCAACTTGCGTTCAAAGACTCGATGGTTC
ACGGGATTCTGCAATTCACACCAAGTATCGCATTTCGCTACGTTCTTCATCGATGCAAG
AGCCGAGATATCCGTTGCCGAGAGTCATTCTTTATAATACATATCACGTATTACCCCACA
CAATCTTATGTTAGATAGATGGGAAAACGTTTCATTTATCACAATTCCTTGGCACAATCC
GCGCCGGGGTTGGTTATCTTGGCCACATGGGCCAGTCGAGAGGAGATGAAGCAAATGA
CACCAAGCCCCCTCAACACATTTGTAAACATGTTCACAGGTTGTTCTGCTTTGCAGGAT
TCGA

S24-*DT*:

AATGCGTACTATTATCTTCATCCAAAGGTTTGCAGTACAGATGCAACCAACTCTAATGA
CATGCCACATGATGACTTAACGAGGGCAAGCCAAGGCAAACCACCGCTCATCATGAAA
TACATGACATTAATGGTTACTCTATTTCAACCAACCGCACTACTATGAGTACGGGAGAG
CCATATCAGTCCTACACCAATTACTGTTGTAGAGCCTACAATGCGTGACACCCAGGCAG
ACGTGCCCTCGACCAAAAGGCTTGGGGCGCAACTTGCGTTCAAAGACTCGATGGTTC
ACGGGATTCTGCAATTCACACCAAGTATCGCATTTCGCTACGTTCTTCATCGATGCAAG
AGCCGAGATATCCGTTGCCGAGAGTCATTCTTTATAATACATATCACGTATTACCCCACA
CAATCTTATGTTAGATAGATGGGAAAACGTTTCATTTATCACAATTCCTTGGCACAATCC
GCGCCGGGGTTGGTTATCTTGGCCACATGGGCCAGTCGAGAGGAGATGAAGCAAATGA
CACCAAGCCCCCTCAACACATTTGTAAACATGTTCACAGGTTGTTCTGCTTTGCAGGAT
TCGA

S25-*DT*:

AATGCGTACTATTATCTTCATCCAAAGGTTTGCAGTACAGATGCAACCAACTCCAATGA
CATGCCACATGATGACTTAACGAGGGCAAGCCAAGGCAAACCACCGCTCATCATGAAA
TACATGACATTAATGGTTACTCTATTTCAACCAACCGCACTACTATGAGTACGGGAGAG
CCATATCAGTCCTACACCAATTACTGTTGTAGAGCCTACAATGCGTGACACCCAGGCAG
ACGTGCCCTCGACCAAAAGGCTTGGGGCGCAACTTGCGTTCAAAGACTCGATGGTTC
ACGGGATTCTGCAATTCACACCAAGTATCGCATTTCGCTACGTTCTTCATCGATGCAAG
AGCCGAGATATCCGTTGCCGAGAGTCATTCTTTATAATACATATCACGTATTACCCCACA
CAATCTTATGTTAGATAGATGGGAAAACGTTTCATTTATCACAATTCCTTGGCACAATCC
GTGCCGGGGTTGGTTATCTTGGCCACATGGGCCAGTCGAGAGGAGATGGAGCAAATGA
CACCAAGCCCCCTCAACACATTTGTAAACATGTTCACAGGTTGTTCTGCTTTGCAGGAT
TCGA

S26-*DT*:

AATGCGTACTATTATCTTCATCCAAAGGTTTGCAGTACAGATGCAACCAACTCTAATGA
CATGCCACATGATGACTTAACGAGGGCAAGCCAAGGCAAACCACCGCTCATCATGAAA
TACATGACATTAATGGTTACTCTATTTCAACCAACCGCACTACTATGAGTACGGGAGAG
CCATATCAGTCCTACACCAATTACTGTTGTAGAGCCTACAATGCGTGACACCCAGGCAG
ACGTGCCCTCGACCAAAAGGCTTGGGGCGCAACTTGCGTTCAAAGACTCGATGGTTC
ACGGGATTCTGCAATTCACACCAAGTATCGCATTTCGCTACGTTCTTCATCGATGCAAG

AGCCGAGATATCCGTTGCCGAGAGTCATTCTTTATAATACATATCACGTATTACCCCACA
CAATCTTATGTTAGATAGATGGGAAAACGTTTCATTTATCACAATTCCTTGGCACAATCC
GTGCCGGGGTTGGTTATCTTGGCCACATGGGCCAGTCGAGAGGAGATGGAGCAAATGA
CACCAAGCCCCCTCAACACATTTGTAAACATGTTCACAGGTTGTTCTGCTTTGCAGGAT
TCGA

S27-*DT*:

AATGCGTACTATTATCTTCATCCAAAGGTTTGCAGTACAGATGCAACCAACTCCAATGA
CATGCCACATGATGACTTAACGAGGGCAAGCCAAGGCAAACCACCGCTCATCATGAAA
TACATGACATTAATGGTTACTCTATTTCAACCAACCGCACTACTATGAGTACGGGAGAG
CCATATCAGTCCTACACCAATTACTGTTGTAGAGCCTACAATGCGTGACACCCAGGCAG
ACGTGCCCTCGACCAAAAGGCTTGGGGCGCAACTTGCGTTCAAAGACTCGATGGTTC
ACGGGATTCTGCAATTCACACCAAGTATCGCATTTCGCTACGTTCTTCATCGATGCAAG
AGCCGAGATATCCGTTGCCGAGAGTCATTCTTTATAATACATATCACGTATTACCCCACA
CAATCTTATGTTAGATAGATGGGAAAACGTTTCATTTATCACAATTCCTTGGCACAATCC
GTGCCGGGGTTGGTTATCTTGGCCACATGGGCCAGTCGAGAGGAGATGGAGCAAATGA
CACCAAGCCCCCTCAACACATTTGTAAACATGTTCACAGGTTGTTCTGCTTTGCAGGAT
TCGA

S28-*DT*:

AATGCGTACTATTATCTTCATCCAAAGGTTTGCAGTACAGATGCAACCAACTCTAATGA
CATGCCACATGATGACTTAACGAGGGCAAGCCAAGGCAAACCACCGCTCATCATGAAA
TACATGACATTAATGGTTACTCTATTTCAACCAACCGCACTACTATGAGTACGGGAGAG
CCATATCAGTCCTACACCAATTACTGTTGTAGAGCCTACAATGCGTGACACCCAGGCAG
ACGTGCCCTCGACCAAAAGGCTTGGGGCGCAACTTGCGTTCAAAGACTCGATGGTTC
ACGGGATTCTGCAATTCACACCAAGTATCGCATTTCGCTACGTTCTTCATCGATGCAAG
AGCCGAGATATCCGTTGCCGAGAGTCATTCTTTATAATACATATCACGTATTACCCCACA
CAATCTTATGTTAGATAGATGGGAAAACGTTTCATTTATCACAATTCCTTGGCACAATCC
GTGCCGGGGTTGGTTATCTTGGCCACATGGGCCAGTCGAGAGGAGATGGAGCAAATGA
CACCAAGCCCCCTCAACACATTTGTAAACATGTTCACAGGTTGTTCTGCTTTGCAGGAT
TCGA

S29-*DT*:

AATGCGTACTATTATCTTCATCCAAAGGTTTGCAGTACAGATGCAACCAACTCTAATGA
CATGCCACATGATGACTTAACGAGGGCAAGCCAAGGCAAACCACCGCTCATCATGAAA
TACATGACATTAATGGTTACTCTATTTCAACCAACCGCACTACTATGAGTACGGGAGAG
CCATATCAGTCCTACACCAATTACTGTTGTAGAGCCTACAATGCGTGACACCCAGGCAG
ACGTGCCCTCGACCAAAAGGCTTGGGGCGCAACTTGCGTTCAAAGACTCGATGGTTC
ACGGGATTCTGCAATTCACACCAAGTATCGCATTTCGCTACGTTCTTCATCGATGCAAG
AGCCGAGATATCCGTTGCCGAGAGTCATTCTTTATAATACATATCACGTATTACCCCACA
CAATCTTATGTTAGATAGATGGGAAAACGTTTCATTTATCACAATTCCTTGGCACAATCC
GTGCCGGGGTTGGTTATCTTGGCCACATGGGCCAGTCGAGAGGAGATGGAGCAAATGA
CACCAAGCCCCCTCAACACATTTGTAAACATGTTCACAGGTTGTTCTGCTTTGCAGGAT
TCGA

S30-*DT*:

AATGCGTACTATTATCTTCATCCAAAGGTTTGCAGTACAGATGCAACCAACTCCAATGA
CATGCCACATGATGACTTAACGAGGGCAAGCCAAGGCAAACCACCGCTCATCATGAAA

TACATGACATTAATGGTTACTCTATTTCAACCAACCGCACTACTATGAGTACGGGAGAG
CCATATCAGTCCTACACCAATTACTGTTGTAGAGCCTACAATGCGTGACACCCAGGCAG
ACGTGCCCTCGACCAAAAGGCTTGGGGCGCAACTTGCGTTCAAAGACTCGATGGTTC
ACGGGATTCTGCAATTCACACCAAGTATCGCATTTCGCTACGTTCTTCATCGATGCAAG
AGCCGAGATATCCGTTGCCGAGAGTCATTCTTTATAATACATATCACGTATTACCCCACA
CAATCTTATGTTAGATAGATGGGAAAACGTTTCATTTATCACAATTCCTTGGCACAATCC
GTGCCGGGGTTGGTTATCTTGGCCACATGGGCCAGTCGAGAGGAGATGGAGCAAATGA
CACCAAGCCCCCTCAACACATTTGTAAACATGTTCACAGGTTGTTCTGCTTTGCAGGAT
TCGA

S31-*DT*:

AATGCGTACTATTATCTTCATCCAAAGGTTTGCAGTACAGATGCAACCAACTCTAATGA
CATGCCACATGATGACTTAACGAGGGCAAGCCAAGGCAAACCACCGCTCATCATGAAA
TACATGACATTAATGGTTACTCTATTTCAACCAACCGCACTACTATGAGTACGGGAGAG
CCATATCAGTCCTACACCAATTACTGTTGTAGAGCCTACAATGCGTGACACCCAGGCAG
ACGTGCCCTCGACCAAAAGGCTTGGGGCGCAACTTGCGTTCAAAGACTCGATGGTTC
ACGGGATTCTGCAATTCACACCAAGTATCGCATTTCGCTACGTTCTTCATCGATGCAAG
AGCCGAGATATCCGTTGCCGAGAGTCATTCTTTATAATACATATCACGTATTACCCCACA
CAATCTTATGTTAGATAGATGGGAAAACGTTTCATTTATCACAATTCCTTGGCACAATCC
GTGCCGGGGTTGGTTATCTTGGCCACATGGGCCAGTCGAGAGGAGATGGAGCAAATGA
CACCAAGCCCCCTCAACACATTTGTAAACATGTTCACAGGTTGTTCTGCTTTGCAGGAT
TCGA

S32-*DT*:

AATGCGTACTATTATCTTCATCCAAAGGTTTGCAGTACAGATGCAACCAACTCTAATGA
CATGCCACATGATGACTTAACGAGGGCAAGCCAAGGCAAACCACCGCTCATCATGAAA
TACATGACATTAATGGTTACTCTATTTCAACCAACCGCACTACTATGAGTACGGGAGAG
CCATATCAGTCCTACACCAATTACTGTTGTAGAGCCTACAATGCGTGACACCCAGGCAG
ACGTGCCCTCGACCAAAAGGCTTGGGGCGCAACTTGCGTTCAAAGACTCGATGGTTC
ACGGGATTCTGCAATTCACACCAAGTATCGCATTTCGCTACGTTCTTCATCGATGCAAG
AGCCGAGATATCCGTTGCCGAGAGTCATTCTTTATAATACATATCACGTATTACCCCACA
CAATCTTATGTTAGATAGATGGGAAAACGTTTCATTTATCACAATTCCTTGGCACAATCC
GTGCCGGGGTTGGTTATCTTGGCCACATGGGCCAGTCGAGAGGAGATGGAGCAAATGA
CACCAAGCCCCCTCAACACATTTGTAAACATGTTCACAGGTTGTTCTGCTTTGCAGGAT
TCGA

S33-*DT*:

AATGCGTACTATTATCTTCATCCAAAGGTTTGCAGTACAGATGCAACCAACTCTAATGA
CATGCCACATGATGACTTAACGAGGGCAAGCCAAGGCAAACCACCGCTCATCATGAAA
TACATGACATTAATGGTTACTCTATTTCAACCAACCGCACTACTATGAGTACGGGAGAG
CCATATCAGTCCTACACCAATTACTGTTGTAGAGCCTACAATGCGTGACACCCAGGCAG
ACGTGCCCTCGACCAAAAGGCTTGGGGCGCAACTTGCGTTCAAAGACTCGATGGTTC
ACGGGATTCTGCAATTCACACCAAGTATCGCATTTCGCTACGTTCTTCATCGATGCAAG
AGCCGAGATATCCGTTGCCGAGAGTCATTCTTTATAATACATATCACGTATTACCCCACA
CAATCTTATGTTAGATAGATGGGAAAACGTTTCATTTATCACAATTCCTTGGCACAATCC
GTGCCGGGGTTGGTTATCTTGGCCACATGGGCCAGTCGAGAGGAGATGGAGCAAATGA
CACCAAGCCCCCTCAACACATTTGTAAACATGTTCACAGGTTGTTCTGCTTTGCAGGAT

TCGA
S34-*DT*:
AATGCGTACTATTATCTTCATCCAAAGGTTTGCAGTACAGATGCAACCAACTCTAATGA
CATGCCACATGATGACTTAACGAGGGCAAGCCAAGGCAAACCACCGCTCATCATGAAA
TACATGACATTAATGGTTACTCTATTTCAACCAACCGCACTACTATGAGTACGGGAGAG
CCATATCAGTCCTACACCAATTACTGTTGTAGAGCCTACAATGCGTGACACCCAGGCAG
ACGTGCCCTCGACCAAAAGGCTTGGGGCGCAACTTGCGTTCAAAGACTCGATGGTTC
ACGGGATTCTGCAATTCACACCAAGTATCGCATTTCGCTACGTTCTTCATCGATGCAAG
AGCCGAGATATCCGTTGCCGAGAGTCATTCTTTATAATACATATCACGTATTACCCCACA
CAATCTTATGTTAGATAGATGGGAAAACGTTTCATTTATCACAATTCCTTGGCACAATCC
GTGCCGGGGTTGGTTATCTTGGCCACATGGGCCAGTCGAGAGGAGATGGAGCAAATGA
CACCAAGCCCCCTCAACACATTTGTAAACATGTTCACAGGTTGTTCTGCTTTGCAGGAT
TCGA
S35-*DT*:
AATGCGTACTATTATCTTCATCCAAAGGTTTGCAGTACAGATGCAACCAACTCTAATGA
CATGCCACATGATGACTTAACGAGGGCAAGCCAAGGCAAACCACCGCTCATCATGAAA
TACATGACATTAATGGTTACTCTATTTCAACCAACCGCACTACTATGAGTACGGGAGAG
CCATATCAGTCCTACACCAATTACTGTTGTAGAGCCTACAATGCGTGACACCCAGGCAG
ACGTGCCCTCGACCAAAAGGCTTGGGGCGCAACTTGCGTTCAAAGACTCGATGGTTC
ACGGGATTCTGCAATTCACACCAAGTATCGCATTTCGCTACGTTCTTCATCGATGCAAG
AGCCGAGATATCCGTTGCCGAGAGTCATTCTTTATAATACATATCACGTATTACCCCACA
CAATCTTATGTTAGATAGATGGGAAAACGTTTCATTTATCACAATTCCTTGGCACAATCC
GTGCCGGGGTTGGTTATCTTGGCCACATGGGCCAGTCGAGAGGAGATGGAGCAAATGA
CACCAAGCCCCCTCAACACATTTGTAAACATGTTCACAGGTTGTTCTGCTTTGCAGGAT
TCGA
S36-*DT*:
AATGCGTACTATTATCTTCATCCAAAGGTTTGCAGTACAGATGCAACCAACTCTAATGA
CATGCCACATGATGACTTAACGAGGGCAAGCCAAGGCAAACCACCGCTCATCATGAAA
TACATGACATTAATGGTTACTCTATTTCAACCAACCGCACTACTATGAGTACGGGAGAG
CCATATCAGTCCTACACCAATTACTGTTGTAGAGCCTACAATGCGTGACACCCAGGCAG
ACGTGCCCTCGACCAAAAGGCTTGGGGCGCAACTTGCGTTCAAAGACTCGATGGTTC
ACGGGATTCTGCAATTCACACCAAGTATCGCATTTCGCTACGTTCTTCATCGATGCAAG
AGCCGAGATATCCGTTGCCGAGAGTCATTCTTTATAATACATATCACGTATTACCCCACA
CAATCTTATGTTAGATAGATGGGAAAACGTTTCATTTATCACAATTCCTTGGCACAATCC
GTGCCGGGGTTGGTTATCTTGGCCACATGGGCCAGTCGAGAGGAGATGGAGCAAATGA
CACCAAGCCCCCTCAACACATTTGTAAACATGTTCACAGGTTGTTCTGCTTTGCAGGAT
TCGA
S37-*DT*:
AATGCGTACTATTATCTTCATCCAAAGGTTTGCAGTACAGATGCAACCAACTCTAATGA
CATGCCACATGATGACTTAACGAGGGCAAGCCAAGGCAAACCACCGCTCATCATGAAA
TACATGACATTAATGGTTACTCTATTTCAACCAACCGCACTACTATGAGTACGGGAGAG
CCATATCAGTCCTACACCAATTACTGTTGTAGAGCCTACAATGCGTGACACCCAGGCAG
ACGTGCCCTCGACCAAAAGGCTTGGGGCGCAACTTGCGTTCAAAGACTCGATGGTTC
ACGGGATTCTGCAATTCACACCAAGTATCGCATTTCGCTACGTTCTTCATCGATGCAAG

AGCCGAGATATCCGTTGCCGAGAGTCATTCTTTATAATACATATCACGTATTACCCCACA
CAATCTTATGTTAGATAGATGGGAAAACGTTTCATTTATCACAATTCCTTGGCACAATCC
GTGCCGGGGTTGGTTATCTTGGCCACATGGGCCAGTCGAGAGGAGATGGAGCAAATGA
CACCAAGCCCCCTCAACACATTTGTAAACATGTTCACAGGTTGTTCTGCTTTGCAGGAT
TCGA

S38-*DT*:

AATGCGTACTATTATCTTCATCCAAAGGTTTGCAGTACAGATGCAACCAACTCTAATGA
CATGCCACATGATGACTTAACGAGGGCAAGCCAAGGCAAACCACCGCTCATCATGAAA
TACATGACATTAATGGTTACTCTATTTCAACCAACCGCACTACTATGAGTACGGGAGAG
CCATATCAGTCCTACACCAATTACTGTTGTAGAGCCTACAATGCGTGACACCCAGGCAG
ACGTGCCCTCGACCAAAAGGCTTGGGGCGCAACTTGCGTTCAAAGACTCGATGGTTC
ACGGGATTCTGCAATTCACACCAAGTATCGCATTTCGCTACGTTCTTCATCGATGCAAG
AGCCGAGATATCCGTTGCCGAGAGTCATTCTTTATAATACATATCACGTATTACCCCACA
CAATCTTATGTTAGATAGATGGGAAAACGTTTCATTTATCACAATTCCTTGGCACAATCC
GTGCCGGGGTTGGTTATCTTGGCCACATGGGCCAGTCGAGAGGAGATGGAGCAAATGA
CACCAAGCCCCCTCAACACATTTGTAAACATGTTCACAGGTTGTTCTGCTTTGCAGGAT
TCGA

S39-*DT*:

AATGCGTACTATTATCTTCATCCAAAGGTTTGCAGTACAGATGCAACCAACTCTAATGA
CATGCCACATGATGACTTAACGAGGGCAAGCCAAGGCAAACCACCGCTCATCATGAAA
TACATGACATTAATGGTTACTCTATTTCAACCAACCGCACTACTATGAGTACGGGAGAG
CCATATCAGTCCTACACCAATTACTGTTGTAGAGCCTACAATGCGTGACACCCAGGCAG
ACGTGCCCTCGACCAAAAGGCTTGGGGCGCAACTTGCGTTCAAAGACTCGATGGTTC
ACGGGATTCTGCAATTCACACCAAGTATCGCATTTCGCTACGTTCTTCATCGATGCAAG
AGCCGAGATATCCGTTGCCGAGAGTCATTCTTTATAATACATATCACGCATTACCCCACA
CAATCTTATGTTAGATAGATGGGAAAACGTTTCATTTATCACAATTCCTTGGCACAATCC
GTGCCGGGGTTGGTTATCTTGGCCACATGGGCCAGTCGAGAGGAGATGGAGCAAATGA
CACCAAGCCCCCTCAACACATTTGTAAACATGTTCACAGGTTGTTCTGCTTTGCAGGAT
TCGA

S40-*DT*:

AATGCGTACTATTATCTTCATCCAAAGGTTTGCAGTACAGATGCAACCAACTCTAATGA
CATGCCACATGATGACTTAACGAGGGCAAGCCAAGGCAAACCACCGCTCATCATGAAA
TACATGACATTAATGGTTACTCTATTTCAACCAACCGCACTACTATGAGTACGGGAGAG
CCATATCAGTCCTACACCAATTACTGTTGTAGAGCCTACAATGCGTGACACCCAGGCAG
ACGTGCCCTCGACCAAAAGGCTTGGGGCGCAACTTGCGTTCAAAGACTCGATGGTTC
ACGGGATTCTGCAATTCACACCAAGTATCGCATTTCGCTACGTTCTTCATCGATGCAAG
AGCCGAGATATCCGTTGCCGAGAGTCATTCTTTATAATACATATCACGCATTACCCCACA
CAATCTTATGTTAGATAGATGGGAAAACGTTTCATTTATCACAATTCCTTGGCACAATCC
GTGCCGGGGTTGGTTATCTTGGCCACATGGGCCAGTCGAGAGGAGATGGAGCAAATGA
CACCAAGCCCCCTCAACACATTTGTAAACATGTTCACAGGTTGTTCTGCTTTGCAGGAT
TCGA

S41-*DT*:

AATGCGTACTATTATCTTCATCCAAAGGTTTGCAGTACAGATGCAACCAACTCTAATGA
CATGCCACATGATGACTTAACGAGGGCAAGCCAAGGCAAACCACCGCTCATCATGAAA

TACATGACATTAATGGTTACTCTATTTCAACCAACCGCACTACTATGAGTACGGGAGAG
CCATATCAGTCCTACACCAATTACTGTTGTAGAGCCTACAATGCGTGACACCCAGGCAG
ACGTGCCCTCGACCAAAAGGCTTGGGGCGCAACTTGCGTTCAAAGACTCGATGGTTC
ACGGGATTCTGCAATTCACACCAAGTATCGCATTTCGCTACGTTCTTCATCGATGCAAG
AGCCGAGATATCCGTTGCCGAGAGTCATTCTTTATAATACATATCACGTATTACCCCACA
CAATCTTATGTTAGATAGATGGGAAAACGTTTCATTTATCACAATTCCTTGGCACAATCC
GTGCCGGGGTTGGTTATCTTGGCCACATGGGCCAGTCGAGAGGAGATGGAGCAAATGA
CACCAAGCCCCCTCAACACATTTGTAAACATGTTCACAGGTTGTTCTGCTTTGCAGGAT
TCGA

S42-*DT*:

AATGCGTACTATTATCTTCATCCAAAGGTTTGCAGTACAGATGCAACCAACTCTAATGA
CATGCCACATGATGACTTAACGAGGGCAAGCCAAGGCAAACCACCGCTCATCATGAAA
TACATGACATTAATGGTTACTCTATTTCAACCAACCGCACTACTATGAGTACGGGAGAG
CCATATCAGTCCTACACCAATTACTGTTGTAGAGCCTACAATGCGTGACACCCAGGCAG
ACGTGCCCTCGACCAAAAGGCTTGGGGCGCAACTTGCGTTCAAAGACTCGATGGTTC
ACGGGATTCTGCAATTCACACCAAGTATCGCATTTCGCTACGTTCTTCATCGATGCAAG
AGCCGAGATATCCGTTGCCGAGAGTCATTCTTTATAATACATATCACGTATTACCCCACA
CAATCTTATGTTAGATAGATGGGAAAACGTTTCATTTATCACAATTCCTTGGCACAATCC
GTGCCGGGGTTGGTTATCTTGGCCACATGGGCCAGTCGAGAGGAGATGGAGCAAATGA
CACCAAGCCCCCTCAACACATTTGTAAACATGTTCACAGGTTGTTCTGCTTTGCAGGAT
TCGA

S43-*DT*:

AATGCGTACTATTATCTTCATCCAAAGGTTTGCAGTACAGATGCAACCAACTCNAATGA
CATGCCACATGATGACTTAACGAGGGCAAGCCAAGGCAAACCACCGCTCATCATGAAA
TACATGACATTAATGGTTACTCTATTTCAACCAACCGCACTACTATGAGTACGGGAGAG
CCATATCAGTCCTACACCAATTACTGTTGTAGAGCCTACAATGCGTGACACCCAGGCAG
ACGTGCCCTCGACCAAAAGGCTTGGGGCGCAACTTGCGTTCAAAGACTCGATGGTTC
ACGGGATTCTGCAATTCACACCAAGTATCGCATTTCGCTACGTTCTTCATCGATGCAAG
AGCCGAGATATCCGTTGCCGAGAGTCATTCTTTATAATACATATCACGNATTACCCCACA
CAATCTTATGTTAGATAGATGGGAAAACGTTTCATTTATCACAATTCCTTGGCACAATCC
GTGCCGGGGTTGGTTATCTTGGCCACATGGGCCAGTCGAGAGGAGATGGAGCAAATGA
CACCAAGCCCCCTCAACACATTTGTAAACATGTTCACAGGTTGTTCTGCTTTGCAGGAT
TCGA

S44-*DT*:

AATGCGTACTATTATCTTCATCCAAAGGTTTGCAGTACAGATGCAACCAACTCCAATGA
CATGCCACATGATGACTTAACGAGGGCAAGCCAAGGCAAACCACCGCTCATCATGAAA
TACATGACATTAATGGTTACTCTATTTCAACCAACCGCACTACTATGAGTACGGGAGAG
CCATATCAGTCCTACACCAATTACTGTTGTAGAGCCTACAATGCGTGACACCCAGGCAG
ACGTGCCCTCGACCAAAAGGCTTGGGGCGCAACTTGCGTTCAAAGACTCGATGGTTC
ACGGGATTCTGCAATTCACACCAAGTATCGCATTTCGCTACGTTCTTCATCGATGCAAG
AGCCGAGATATCCGTTGCCGAGAGTCATTCTTTATAATACATATCACGTATTACCCCACA
CAATCTTATGTTAGATAGATGGGAAAACGTTTCATTTATCACAATTCCTTGGCACAATCC
GTGCCGGGGTTGGTTATCTTGGCCACATGGGCCAGTCGAGAGGAGATGGAGCAAATGA
CACCAAGCCCCCTCAACACATTTGTAAACATGTTCACAGGTTGTTCTGCTTTGCAGGAT

TCGA
S45-*DT*:
AATGCGTACTATTATCTTCATCCAAAGGTTTGCAGTACAGATGCAACCAACTCTAATGA
CATGCCACATGATGACTTAACGAGGGCAAGCCAAGGCAAACCACCGCTCATCATGAAA
TACATGACATTAATGGTTACTCTATTTCAACCAACCGCACTACTATGAGTACGGGAGAG
CCATATCAGTCCTACACCAATTACTGTTGTAGAGCCTACAATGCGTGACACCCAGGCAG
ACGTGCCCTCGACCAAAAGGCTTGGGGCGCAACTTGCGTTCAAAGACTCGATGGTTC
ACGGGATTCTGCAATTCACACCAAGTATCGCATTTCGCTACGTTCTTCATCGATGCAAG
AGCCGAGATATCCGTTGCCGAGAGTCATTCTTTATAATACATATCACGTATTACCCCACA
CAATCTTATGTTAGATAGATGGGAAAACGTTTCATTTATCACAATTCCTTGGCACAATCC
GTGCCGGGGTTGGTTATCTTGGCCACATGGGCCAGTCGAGAGGAGATGGAGCAAATGA
CACCAAGCCCCCTCAACACATTTGTAAACATGTTCACAGGTTGTTCTGCTTTGCAGGAT
TCGA
S46-*DT*:
AATGCGTACTATTATCTTCATCCAAAGGTTTGCAGTACAGATGCAACCAACTCCAATGA
CATGCCACATGATGACTTAACGAGGGCAAGCCAAGGCAAACCACCGCTCATCATGAAA
TACATGACATTAATGGTTACTCTATTTCAACCAACCGCACTACTATGAGTACGGGAGAG
CCATATCAGTCCTACACCAATTACTGTTGTAGAGCCTACAATGCGTGACACCCAGGCAG
ACGTGCCCTCGACCAAAAGGCTTGGGGCGCAACTTGCGTTCAAAGACTCGATGGTTC
ACGGGATTCTGCAATTCACACCAAGTATCGCATTTCGCTACGTTCTTCATCGATGCAAG
AGCCGAGATATCCGTTGCCGAGAGTCATTCTTTATAATACATATCACGTATTACCCCACA
CAATCTTATGTTAGATAGATGGGAAAACGTTTCATTTATCACAATTCCTTGGCACAATCC
GTGCCGGGGTTGGTTATCTTGGCCACATGGGCCAGTCGAGAGGAGATGGAGCAAATGA
CACCAAGCCCCCTCAACACATTTGTAAACATGTTCACAGGTTGTTCTGCTTTGCAGGAT
TCGA
S47-*DT*:
AATGCGTACTATTATCTTCATCCAAAGGTTTGCAGTACAGATGCAACCAACTCCAATGA
CATGCCACATGATGACTTAACGAGGGCAAGCCAAGGCAAACCACCGCTCATCATGAAA
TACATGACATTAATGGTTACTCTATTTCAACCAACCGCACTACTATGAGTACGGGAGAG
CCATATCAGTCCTACACCAATTACTGTTGTAGAGCCTACAATGCGTGACACCCAGGCAG
ACGTGCCCTCGACCAAAAGGCTTGGGGCGCAACTTGCGTTCAAAGACTCGATGGTTC
ACGGGATTCTGCAATTCACACCAAGTATCGCATTTCGCTACGTTCTTCATCGATGCAAG
AGCCGAGATATCCGTTGCCGAGAGTCATTCTTTATAATACATATCACGTATTACCCCACA
CAATCTTATGTTAGATAGATGGGAAAACGTTTCATTTATCACAATTCCTTGGCACAATCC
GTGCCGGGGTTGGTTATCTTGGCCACATGGGCCAGTCGAGAGGAGATGGAGCAAATGA
CACCAAGCCCCCTCAACACATTTGTAAACATGTTCACAGGTTGTTCTGCTTTGCAGGAT
TCGA
S48-*DT*:
AATGCGTACTATTATCTTCATCCAAAGGTTTGCAGTACAGATGCAACCAACTCTAATGA
CATGCCACATGATGACTTAACGAGGGCAAGCCAAGGCAAACCACCGCTCATCATGAAA
TACATGACATTAATGGTTACTCTATTTCAACCAACCGCACTACTATGAGTACGGGAGAG
CCATATCAGTCCTACACCAATTACTGTTGTAGAGCCTACAATGCGTGACACCCAGGCAG
ACGTGCCCTCGACCAAAAGGCTTGGGGCGCAACTTGCGTTCAAAGACTCGATGGTTC
ACGGGATTCTGCAATTCACACCAAGTATCGCATTTCGCTACGTTCTTCATCGATGCAAG

AGCCGAGATATCCGTTGCCGAGAGTCATTCTTTATAATACATATCACGTATTACCCCACA
CAATCTTATGTTAGATAGATGGGAAAACGTTTCATTTATCACAATTCCTTGGCACAATCC
GTGCCGGGGTTGGTTATCTTGGCCACATGGGCCAGTCGAGAGGAGATGGAGCAAATGA
CACCAAGCCCCCTCAACACATTTGTAAACATGTTCACAGGTTGTTCTGCTTTGCAGGAT
TCGA

S49-*DT*:

AATGCGTACTATTATCTTCATCCAAAGGTTTGCAGTACAGATGCAACCAACTCCAATGA
CATGCCACATGATGACTTAACGAGGGCAAGCCAAGGCAAACCACCGCTCATCATGAAA
TACATGACATTAATGGTTACTCTATTTCAACCAACCGCACTACTATGAGTACGGGAGAG
CCATATCAGTCCTACACCAATTACTGTTGTAGAGCCTACAATGCGTGACACCCAGGCAG
ACGTGCCCTCGACCAAAAGGCTTGGGGCGCAACTTGCGTTCAAAGACTCGATGGTTC
ACGGGATTCTGCAATTCACACCAAGTATCGCATTTCGCTACGTTCTTCATCGATGCAAG
AGCCGAGATATCCGTTGCCGAGAGTCATTCTTTATAATACATATCACGTATTACCCCACA
CAATCTTATGTTAGATAGATGGGAAAACGTTTCATTTATCACAATTCCTTGGCACAATCC
GTGCCGGGGTTGGTTATCTTGGCCACATGGGCCAGTCGAGAGGAGATGGAGCAAATGA
CACCAAGCCCCCTCAACACATTTGTAAACATGTTCACAGGTTGTTCTGCTTTGCAGGAT
TCGA

S50-*DT*:

AATGCGTACTATTATCTTCATCCAAAGGTTTGCAGTACAGATGCAACCAACTCCAATGA
CATGCCACATGATGACTTAACGAGGGCAAGCCAAGGCAAACCACCGCTCATCATGAAA
TACATGACATTAATGGTTACTCTATTTCAACCAACCGCACTACTATGAGTACGGGAGAG
CCATATCAGTCCTACACCAATTACTGTTGTAGAGCCTACAATGCGTGACACCCAGGCAG
ACGTGCCCTCGACCAAAAGGCTTGGGGCGCAACTTGCGTTCAAAGACTCGATGGTTC
ACGGGATTCTGCAATTCACACCAAGTATCGCATTTCGCTACGTTCTTCATCGATGCAAG
AGCCGAGATATCCGTTGCCGAGAGTCATTCTTTATAATACATATCACGTATTACCCCACA
CAATCTTATGTTAGATAGATGGGAAAACGTTTCATTTATCACAATTCCTTGGCACAATCC
GTGCCGGGGTTGGTTATCTTGGCCACATGGGCCAGTCGAGAGGAGATGGAGCAAATGA
CACCAAGCCCCCTCAACACATTTGTAAACATGTTCACAGGTTGTTCTGCTTTGCAGGAT
TCGA

S51-*DT*:

AATGCGTACTATTATCTTCATCCAAAGGTTTGCAGTACAGATGCAACCAACTCCAATGA
CATGCCACATGATGACTTAACGAGGGCAAGCCAAGGCAAACCACCGCTCATCATGAAA
TACATGACATTAATGGTTACTCTATTTCAACCAACCGCACTACTATGAGTACGGGAGAG
CCATATCAGTCCTACACCAATTACTGTTGTAGAGCCTACAATGCGTGACACCCAGGCAG
ACGTGCCCTCGACCAAAAGGCTTGGGGCGCAACTTGCGTTCAAAGACTCGATGGTTC
ACGGGATTCTGCAATTCACACCAAGTATCGCATTTCGCTACGTTCTTCATCGATGCAAG
AGCCGAGATATCCGTTGCCGAGAGTCATTCTTTATAATACATATCACGTATTACCCCACA
CAATCTTATGTTAGATAGATGGGAAAACGTTTCATTTATCACAATTCCTTGGCACAATCC
GTGCCGGGGTTGGTTATCTTGGCCACATGGGCCAGTCGAGAGGAGATGGAGCAAATGA
CACCAAGCCCCCTCAACACATTTGTAAACATGTTCACAGGTTGTTCTGCTTTGCAGGAT
TCGA

S52-*DT*:

AATGCGTACTATTATCTTCATCCAAAGGTTTGCAGTACAGATGCAACCAACTCCAATGA
CATGCCACATGATGACTTAACGAGGGCAAGCCAAGGCAAACCACCGCTCATCATGAAA

TACATGACATTAATGGTTACTCTATTTCAACCAACCGCACTACTATGAGTACGGGAGAG
CCATATCAGTCCTACACCAATTACTGTTGTAGAGCCTACAATGCGTGACACCCAGGCAG
ACGTGCCCTCGACCAAAAGGCTTGGGGCGCAACTTGCGTTCAAAGACTCGATGGTTC
ACGGGATTCTGCAATTCACACCAAGTATCGCATTTCGCTACGTTCTTCATCGATGCAAG
AGCCGAGATATCCGTTGCCGAGAGTCATTCTTTATAATACATATCACGTATTACCCCACA
CAATCTTATGTTAGATAGATGGGAAAACGTTTCATTTATCACAATTCCTTGGCACAATCC
GTGCCGGGGTTGGTTATCTTGGCCACATGGGCCAGTCGAGAGGAGATGGAGCAAATGA
CACCAAGCCCCCTCAACACATTTGTAAACATGTTCACAGGTTGTTCTGCTTTGCAGGAT
TCGA

S53-*DT*:

AATGCGTACTATTATCTTCATCCAAAGGTTTGCAGTACAGATGCAACCAACTCTAATGA
CATGCCACATGATGACTTAACGAGGGCAAGCCAAGGCAAACCACCGCTCATCATGAAA
TACATGACATTAATGGTTACTCTATTTCAACCAACCGCACTACTATGAGTACGGGAGAG
CCATATCAGTCCTACACCAATTACTGTTGTAGAGCCTACAATGCGTGACACCCAGGCAG
ACGTGCCCTCGACCAAAAGGCTTGGGGCGCAACTTGCGTTCAAAGACTCGATGGTTC
ACGGGATTCTGCAATTCACACCAAGTATCGCATTTCGCTACGTTCTTCATCGATGCAAG
AGCCGAGATATCCGTTGCCGAGAGTCATTCTTTATAATACATATCACGTATTACCCCACA
CAATCTTATGTTAGATAGATGGGAAAACGTTTCATTTATCACAATTCCTTGGCACAATCC
GTGCCGGGGTTGGTTATCTTGGCCACATGGGCCAGTCGAGAGGAGATGGAGCAAATGA
CACCAAGCCCCCTCAACACATTTGTAAACATGTTCACAGGTTGTTCTGCTTTGCAGGAT
TCGA

S54-*DT*:

AATGCGTACTATTATCTTCATCCAAAGGTTTGCAGTACAGATGCAACCAACTCTAATGA
CATGCCACATGATGACTTAACGAGGGCAAGCCAAGGCAAACCACCGCTCATCATGAAA
TACATGACATTAATGGTTACTCTATTTCAACCAACCGCACTACTATGAGTACGGGAGAG
CCATATCAGTCCTACACCAATTACTGTTGTAGAGCCTACAATGCGTGACACCCAGGCAG
ACGTGCCCTCGACCAAAAGGCTTGGGGCGCAACTTGCGTTCAAAGACTCGATGGTTC
ACGGGATTCTGCAATTCACACCAAGTATCGCATTTCGCTACGTTCTTCATCGATGCAAG
AGCCGAGATATCCGTTGCCGAGAGTCATTCTTTATAATACATATCACGTATTACCCCACA
CAATCTTATGTTAGATAGATGGGAAAACGTTTCATTTATCACAATTCCTTGGCACAATCC
GTGCCGGGGTTGGTTATCTTGGCCACATGGGCCAGTCGAGAGGAGATGGAGCAAATGA
CACCAAGCCCCCTCAACACATTTGTAAACATGTTCACAGGTTGTTCTGCTTTGCAGGAT
TCGA

S55-*DT*:

AATGCGTACTATTATCTTCATCCAAAGGTTTGCAGTACAGATGCAACCAACTCTAATGA
CATGCCACATGATGACTTAACGAGGGCAAGCCAAGGCAAACCACCGCTCATCATGAAA
TACATGACATTAATGGTTACTCTATTTCAACCAACCGCACTACTATGAGTACGGGAGAG
CCATATCAGTCCTACACCAATTACTGTTGTAGAGCCTACAATGCGTGACACCCAGGCAG
ACGTGCCCTCGACCAAAAGGCTTGGGGCGCAACTTGCGTTCAAAGACTCGATGGTTC
ACGGGATTCTGCAATTCACACCAAGTATCGCATTTCGCTACGTTCTTCATCGATGCAAG
AGCCGAGATATCCGTTGCCGAGAGTCATTCTTTATAATACATATCACGTATTACCCCACA
CAATCTTATGTTAGATAGATGGGAAAACGTTTCATTTATCACAATTCCTTGGCACAATCC
GTGCCGGGGTTGGTTATCTTGGCCACATGGGCCAGTCGAGAGGAGATGGAGCAAATGA
CACCAAGCCCCCTCAACACATTTGTAAACATGTTCACAGGTTGTTCTGCTTTGCAGGAT

TCGA

S56-*DT*:

AATGCGTACTATTATCTTCATCCAAAGGTTTGCAGTACAGATGCAACCAACTCTAATGA
CATGCCACATGATGACTTAACGAGGGCAAGCCAAGGCAAACCACCGCTCATCATGAAA
TACATGACATTAATGGTTACTCTATTTCAACCAACCGCACTACTATGAGTACGGGAGAG
CCATATCAGTCCTACACCAATTACTGTTGTAGAGCCTACAATGCGTGACACCCAGGCAG
ACGTGCCCTCGACCAAAAGGCTTGGGGCGCAACTTGCGTTCAAAGACTCGATGGTTC
ACGGGATTCTGCAATTCACACCAAGTATCGCATTTCGCTACGTTCTTCATCGATGCAAG
AGCCGAGATATCCGTTGCCGAGAGTCATTCTTTATAATACATATCACGTATTACCCCACA
CAATCTTATGTTAGATAGATGGGAAAACGTTTCATTTATCACAATTCCTTGGCACAATCC
GTGCCGGGGTTGGTTATCTTGGCCACATGGGCCAGTCGAGAGGAGATGGAGCAAATGA
CACCAAGCCCCCTCAACACATTTGTAAACATGTTCACAGGTTGTTCTGCTTTGCAGGAT
TCGA

S57-*DT*:

AATGCGTACTATTATCTTCATCCAAAGGTTTGCAGTACAGATGCAACCAACTCTAATGA
CATGCCACATGATGACTTAACGAGGGCAAGCCAAGGCAAACCACCGCTCATCATGAAA
TACATGACATTAATGGTTACTCTATTTCAACCAACCGCACTACTATGAGTACGGGAGAG
CCATATCAGTCCTACACCAATTACTGTTGTAGAGCCTACAATGCGTGACACCCAGGCAG
ACGTGCCCTCGACCAAAAGGCTTGGGGCGCAACTTGCGTTCAAAGACTCGATGGTTC
ACGGGATTCTGCAATTCACACCAAGTATCGCATTTCGCTACGTTCTTCATCGATGCAAG
AGCCGAGATATCCGTTGCCGAGAGTCATTCTTTATAATACATATCACGTATTACCCCACA
CAATCTTATGTTAGATAGATGGGAAAACGTTTCATTTATCACAATTCCTTGGCACAATCC
GTGCCGGGGTTGGTTATCTTGGCCACATGGGCCAGTCGAGAGGAGATGGAGCAAATGA
CACCAAGCCCCCTCAACACATTTGTAAACATGTTCACAGGTTGTTCTGCTTTGCAGGAT
TCGA

S58-*DT*:

AATGCGTACTATTATCTTCATCCAAAGGTTTGCAGTACAGATGCAACCAACTCTAATGA
CATGCCACATGATGACTTAACGAGGGCAAGCCAAGGCAAACCACCGCTCATCATGAAA
TACATGACATTAATGGTTACTCTATTTCAACCAACCGCACTACTATGAGTACGGGAGAG
CCATATCAGTCCTACACCAATTACTGTTGTAGAGCCTACAATGCGTGACACCCAGGCAG
ACGTGCCCTCGACCAAAAGGCTTGGGGCGCAACTTGCGTTCAAAGACTCGATGGTTC
ACGGGATTCTGCAATTCACACCAAGTATCGCATTTCGCTACGTTCTTCATCGATGCAAG
AGCCGAGATATCCGTTGCCGAGAGTCATTCTTTATAATACATATCACGTATTACCCCACA
CAATCTCATGTTAGATAGATGGGAAAACGTTTCATTTATCACAATTCCTTGGCACAATCC
GCGCCGGGGTTGGTTATCTTGGCCACATGGGCCAGTCGAGAGGAGATGAAGCAAATGA
CACCAAGCCCCCTCAACACATTTGTAAACATGTTCACAGGTTGTTCTGCTTTGCAGGAT
TCGA

S59-*DT*:

AATGCGTACTATTATCTTCATCCAAAGGTTTGCAGTACAGATGCAACCAACTCTAATGA
CATGCCACATGATGACTTAACGAGGGCAAGCCAAGGCAAACCACCGCTCATCATGAAA
TACATGACATTAATGGTTACTCTATTTCAACCAACCGCACTACTATGAGTACGGGAGAG
CCATATCAGTCCTACACCAATTACTGTTGTAGAGCCTACAATGCGTGACACCCAGGCAG
ACGTGCCCTCGACCAAAAGGCTTGGGGCGCAACTTGCGTTCAAAGACTCGATGGTTC
ACGGGATTCTGCAATTCACACCAAGTATCGCATTTCGCTACGTTCTTCATCGATGCAAG

AGCCGAGATATCCGTTGCCGAGAGTCATTCTTTATAATACATATCACGTATTACCCCACA
CAATCTCATGTTAGATAGATGGGAAAACGTTTCATTTATCACAATTCCTTGGCACAATCC
GCGCCGGGGTTGGTTATCTTGGCCACATGGGCCAGTCGAGAGGAGATGAAGCAAATGA
CACCAAGCCCCCTCAACACATTTGTAAACATGTTCACAGGTTGTTCTGCTTTGCAGGAT
TCGA

S60-*DT*:

AATGCGTACTATTATCTTCATCCAAAGGTTTGCAGTACAGATGCAACCAACTCTAATGA
CATGCCACATGATGACTTAACGAGGGCAAGCCAAGGCAAACCACCGCTCATCATGAAA
TACATGACATTAATGGTTACTCTATTTCAACCAACCGCACTACTATGAGTACGGGAGAG
CCATATCAGTCCTACACCAATTACTGTTGTAGAGCCTACAATGCGTGACACCCAGGCAG
ACGTGCCCTCGACCAAAAGGCTTGGGGCGCAACTTGCGTTCAAAGACTCGATGGTTC
ACGGGATTCTGCAATTCACACCAAGTATCGCATTTCGCTACGTTCTTCATCGATGCAAG
AGCCGAGATATCCGTTGCCGAGAGTCATTCTTTATAATACATATCACGTATTACCCCACA
CAATCTCATGTTAGATAGATGGGAAAACGTTTCATTTATCACAATTCCTTGGCACAATCC
GCGCCGGGGTTGGTTATCTTGGCCACATGGGCCAGTCGAGAGGAGATGAAGCAAATGA
CACCAAGCCCCCTCAACACATTTGTAAACATGTTCACAGGTTGTTCTGCTTTGCAGGAT
TCGA

S61-*DT*:

AATGCGTACTATTATCTTCATCCAAAGGTTTGCAGTACAGATGCAACCAACTCTAATGA
CATGCCACATGATGACTTAACGAGGGCAAGCCAAGGCAAACCACCGCTCATCATGAAA
TACATGACATTAATGGTTACTCTATTTCAACCAACCGCACTACTATGAGTACGGGAGAG
CCATATCAGTCCTACACCAATTACTGTTGTAGAGCCTACAATGCGTGACACCCAGGCAG
ACGTGCCCTCGACCAAAAGGCTTGGGGCGCAACTTGCGTTCAAAGACTCGATGGTTC
ACGGGATTCTGCAATTCACACCAAGTATCGCATTTCGCTACGTTCTTCATCGATGCAAG
AGCCGAGATATCCGTTGCCGAGAGTCATTCTTTATAATACATATCACGTATTACCCCACA
CAATCTCATGTTAGATAGATGGGAAAACGTTTCATTTATCACAATTCCTTGGCACAATCC
GCGCCGGGGTTGGTTATCTTGGCCACATGGGCCAGTCGAGAGGAGATGAAGCAAATGA
CACCAAGCCCCCTCAACACATTTGTAAACATGTTCACAGGTTGTTCTGCTTTGCAGGAT
TCGA

S62-*DT*:

AATGCGTACTATTATCTTCATCCAAAGGTTTGCAGTACAGATGCAACCAACTCTAATGA
CATGCCACATGATGACTTAACGAGGGCAAGCCAAGGCAAACCACCGCTCATCATGAAA
TACATGACATTAATGGTTACTCTATTTCAACCAACCGCACTACTATGAGTACGGGAGAG
CCATATCAGTCCTACACCAATTACTGTTGTAGAGCCTACAATGCGTGACACCCAGGCAG
ACGTGCCCTCGACCAAAAGGCTTGGGGCGCAACTTGCGTTCAAAGACTCGATGGTTC
ACGGGATTCTGCAATTCACACCAAGTATCGCATTTCGCTACGTTCTTCATCGATGCAAG
AGCCGAGATATCCGTTGCCGAGAGTCATTCTTTATAATACATATCACGTATTACCCCACA
CAATCTCATGTTAGATAGATGGGAAAACGTTTCATTTATCACAATTCCTTGGCACAATCC
GCGCCGGGGTTGGTTATCTTGGCCACATGGGCCAGTCGAGAGGAGATGAAGCAAATGA
CACCAAGCCCCCTCAACACATTTGTAAACATGTTCACAGGTTGTTCTGCTTTGCAGGAT
TCGA

S63-*DT*:

AATGCGTACTATTATCTTCATCCAAAGGTTTGCAGTACAGATGCAACCAACTCTAATGA
CATGCCACATGATGACTTAACGAGGGCAAGCCAAGGCAAACCACCGCTCATCATGAAA

TACATGACATTAATGGTTACTCTATTTCAACCAACCGCACTACTATGAGTACGGGAGAG
CCATATCAGTCCTACACCAATTACTGTTGTAGAGCCTACAATGCGTGACACCCAGGCAG
ACGTGCCCTCGACCAAAAGGCTTGGGGCGCAACTTGCGTTCAAAGACTCGATGGTTC
ACGGGATTCTGCAATTCACACCAAGTATCGCATTTCGCTACGTTCTTCATCGATGCAAG
AGCCGAGATATCCGTTGCCGAGAGTCATTCTTTATAATACATATCACGTATTACCCCACA
CAATCTCATGTTAGATAGATGGGAAAACGTTTCATTTATCACAATTCCTTGGCACAATCC
GCGCCGGGGTTGGTTATCTTGGCCACATGGGCCAGTCGAGAGGAGATGAAGCAAATGA
CACCAAGCCCCCTCAACACATTTGTAAACATGTTCACAGGTTGTTCTGCTTTGCAGGAT
TCGA

S64-*DT*:

AATGCGTACTATTATCTTCATCCAAAGGTTTGCAGTACAGATGCAACCAACTCTAATGA
CATGCCACATGATGACTTAACGAGGGCAAGCCAAGGCAAACCACCGCTCATCATGAAA
TACATGACATTAATGGTTACTCTATTTCAACCAACCGCACTACTATGAGTACGGGAGAG
CCATATCAGTCCTACACCAATTACTGTTGTAGAGCCTACAATGCGTGACACCCAGGCAG
ACGTGCCCTCGACCAAAAGGCTTGGGGCGCAACTTGCGTTCAAAGACTCGATGGTTC
ACGGGATTCTGCAATTCACACCAAGTATCGCATTTCGCTACGTTCTTCATCGATGCAAG
AGCCGAGATATCCGTTGCCGAGAGTCATTCTTTATAATACATATCACGTATTACCCCACA
CAATCTCATGTTAGATAGATGGGAAAACGTTTCATTTATCACAATTCCTTGGCACAATCC
GCGCCGGGGTTGGTTATCTTGGCCACATGGGCCAGTCGAGAGGAGATGAAGCAAATGA
CACCAAGCCCCCTCAACACATTTGTAAACATGTTCACAGGTTGTTCTGCTTTGCAGGAT
TCGA

S65-*DT*:

AATGCGTACTATTATCTTCATCCAAAGGTTTGCAGTACAGATGCAACCAACTCTAATGA
CATGCCACATGATGACTTAACGAGGGCAAGCCAAGGCAAACCACCGCTCATCATGAAA
TACATGACATTAATGGTTACTCTATTTCAACCAACCGCACTACTATGAGTACGGGAGAG
CCATATCAGTCCTACACCAATTACTGTTGTAGAGCCTACAATGCGTGACACCCAGGCAG
ACGTGCCCTCGACCAAAAGGCTTGGGGCGCAACTTGCGTTCAAAGACTCGATGGTTC
ACGGGATTCTGCAATTCACACCAAGTATCGCATTTCGCTACGTTCTTCATCGATGCAAG
AGCCGAGATATCCGTTGCCGAGAGTCATTCTTTATAATACATATCACGTATTACCCCACA
CAATCTCATGTTAGATAGATGGGAAAACGTTTCATTTATCACAATTCCTTGGCACAATCC
GCGCCGGGGTTGGTTATCTTGGCCACATGGGCCAGTCGAGAGGAGATGAAGCAAATGA
CACCAAGCCCCCTCAACACATTTGTAAACATGTTCACAGGTTGTTCTGCTTTGCAGGAT
TCGA

S66-*DR*:

AATGCGTACTATTATCTTCATCCAAAGGTTTGCAGTACAGATGCAACCAACTCTAATGA
AATGCCACATGATGACTTAACGAGGGCAAGCCAAGGCAAACCACCGCTCATCATGAAA
TACATGACATTAATGGTTACTCTATTTCAACCAACCACACTACTATGAGTACGGGAGAG
CCATATCAGTCCTACACCAATTACTGTTGTAGAGCCTACAATGCGTGACACCCAGGCAG
ACGTGCCCTCGACCAAAAGGCTTGGGGCGCAACTTGCGTTCAAAGACTCGATGGTTC
ACGGGATTCTGCAATTCACACCAAGTATCGCATTTCGCTACGTTCTTCATCGATGCAAG
AGCCGAGATATCCGTTGCCGAGAGTCATTCTTTATAATACATATCACGTATTACCCCACA
CAATCTTATGTTAGATAGATGGGAAAACGTTTCATTTATCACAATTCCTTGGCACAATCC
GTGCCGGGGTTGGTTATCTTGGCCACATGGGCCAGTCGAGAGGAGATGGAGCAAATGA
CACCAAGCCCCCTCAACACATTTGTAAACATGTTCACAGGTTGTTCTGCTTTGCAGGAT

TCGA

S67-*DR*:

AATGCGTACTATTATCTTCATCCAAAGGTTTGCAGTACAGATGCAACCAACTCTAATGA
AATGCCACATGATGACTTAACGAGGGCAAGCCAAGGCAAACCACCGCTCATCATGAAA
TACATGACATTAATGGTTACTCTATTTCAACCAACCACACTACTATGAGTACGGGAGAG
CCATATCAGTCCTACACCAATTACTGTTGTAGAGCCTACAATGCGTGACACCCAGGCAG
ACGTGCCCTCGACCAAAAGGCTTGGGGCGCAACTTGCGTTCAAAGACTCGATGGTTC
ACGGGATTCTGCAATTCACACCAAGTATCGCATTTCGCTACGTTCTTCATCGATGCAAG
AGCCGAGATATCCGTTGCCGAGAGTCATTCTTTATAATACATATCACGTATTACCCCACA
CAATCTTATGTTAGATAGATGGGAAAACGTTTCATTTATCACAATTCCTTGGCACAATCC
GTGCCGGGGTTGGTTATCTTGGCCACATGGGCCAGTCGAGAGGAGATGGAGCAAATGA
CACCAAGCCCCCTCAACACATTTGTAAACATGTTCACAGGTTGTTCTGCTTTGCAGGAT
TCGA

S68-*DR*:

AATGCGTACTATTATCTTCATCCAAAGGTTTGCAGTACAGATGCAACCAACTCTAATGA
AATGCCACATGATGACTTAACGAGGGCAAGCCAAGGCAAACCACCGCTCATCATGAAA
TACATGACATTAATGGTTACTCTATTTCAACCAACCACACTACTATGAGTACGGGAGAG
CCATATCAGTCCTACACCAATTACTGTTGTAGAGCCTACAATGCGTGACACCCAGGCAG
ACGTGCCCTCGACCAAAAGGCTTGGGGCGCAACTTGCGTTCAAAGACTCGATGGTTC
ACGGGATTCTGCAATTCACACCAAGTATCGCATTTCGCTACGTTCTTCATCGATGCAAG
AGCCGAGATATCCGTTGCCGAGAGTCATTCTTTATAATACATATCACGTATTACCCCACA
CAATCTTATGTTAGATAGATGGGAAAACGTTTCATTTATCACAATTCCTTGGCACAATCC
GTGCCGGGGTTGGTTATCTTGGCCACATGGGCCAGTCGAGAGGAGATGGAGCAAATGA
CACCAAGCCCCCTCAACACATTTGTAAACATGTTCACAGGTTGTTCTGCTTTGCAGGAT
TCGA

S69-*DR*:

AATGCGTACTATTATCTTCATCCAAAGGTTTGCAGTACAGATGCAACCAACTCTAATGA
AATGCCACATGATGACTTAACGAGGGCAAGCCAAGGCAAACCACCGCTCATCATGAAA
TACATGACATTAATGGTTACTCTATTTCAACCAACCACACTACTATGAGTACGGGAGAG
CCATATCAGTCCTACACCAATTACTGTTGTAGAGCCTACAATGCGTGACACCCAGGCAG
ACGTGCCCTCGACCAAAAGGCTTGGGGCGCAACTTGCGTTCAAAGACTCGATGGTTC
ACGGGATTCTGCAATTCACACCAAGTATCGCATTTCGCTACGTTCTTCATCGATGCAAG
AGCCGAGATATCCGTTGCCGAGAGTCATTCTTTATAATACATATCACGTATTACCCCACA
CAATCTTATGTTAGATAGATGGGAAAACGTTTCATTTATCACAATTCCTTGGCACAATCC
GTGCCGGGGTTGGTTATCTTGGCCACATGGGCCAGTCGAGAGGAGATGGAGCAAATGA
CACCAAGCCCCCTCAACACATTTGTAAACATGTTCACAGGTTGTTCTGCTTTGCAGGAT
TCGA

S70-*DR*:

AATGCGTACTATTATCTTCATCCAAAGGTTTGCAGTACAGATGCAACCAACTCTAATGA
AATGCCACATGATGACTTAACGAGGGCAAGCCAAGGCAAACCACCGCTCATCATGAAA
TACATGACATTAATGGTTACTCTATTTCAACCAACCNCACTACTATGAGTACGGGAGAG
CCATATCAGTCCTACACCAATTACTGTTGTAGAGCCTACAATGCGTGACACCCAGGCAG
ACGTGCCCTCGACCAAAAGGCTTGGGGCGCAACTTGCGTTCAAAGACTCGATGGTTC
ACGGGATTCTGCAATTCACACCAAGTATCGCATTTCGCTACGTTCTTCATCGATGCAAG

AGCCGAGATATCCGTTGCCGAGAGTCATTCTTTATAATACATATCACGTATTACCCCACA
CAATCTTATGTTAGATAGATGGGAAAACGTTTCATTTATCACAATTCCTTGGCACAATCC
GTGCCGGGGTTGGTTATCTTGGCCACATGGGCCAGTCGAGAGGAGATGGAGCAAATGA
CACCAAGCCCCCTCAACACATTTGTAAACATGTTCACAGGTTGTTCTGCTTTGCAGGAT
TCGA

S71-*DR*:

AATGCGTACTATTATCTTCATCCAAAGGTTTGCAGTACAGATGCAACCAACTCTAATGA
AATGCCACATGATGACTTAACGAGGGCAAGCCAAGGCAAACCACCGCTCATCATGAAA
TACATGACATTAATGGTTACTCTATTTCAACCAACCNCACTACTATGAGTACGGGAGAG
CCATATCAGTCCTACACCAATTACTGTTGTAGAGCCTACAATGCGTGACACCCAGGCAG
ACGTGCCCTCGACCAAAAGGCTTGGGGCGCAACTTGCGTTCAAAGACTCGATGGTTC
ACGGGATTCTGCAATTCACACCAAGTATCGCATTTCGCTACGTTCTTCATCGATGCAAG
AGCCGAGATATCCGTTGCCGAGAGTCATTCTTTATAATACATATCACGTATTACCCCACA
CAATCTTATGTTAGATAGATGGGAAAACGTTTCATTTATCACAATTCCTTGGCACAATCC
GTGCCGGGGTTGGTTATCTTGGCCACATGGGCCAGTCGAGAGGAGATGGAGCAAATGA
CACCAAGCCCCCTCAACACATTTGTAAACATGTTCACAGGTTGTTCTGCTTTGCAGGAT
TCGA

S72-*DR*:

AATGCGTACTATTATCTTCATCCAAAGGTTTGCAGTACAGATGCAACCAACTCTAATGA
AATGCCACATGATGACTTAACGAGGGCAAGCCAAGGCAAACCACCGCTCATCATGAAA
TACATGACATTAATGGTTACTCTATTTCAACCAACCNCACTACTATGAGTACGGGAGAG
CCATATCAGTCCTACACCAATTACTGTTGTAGAGCCTACAATGCGTGACACCCAGGCAG
ACGTGCCCTCGACCAAAAGGCTTGGGGCGCAACTTGCGTTCAAAGACTCGATGGTTC
ACGGGATTCTGCAATTCACACCAAGTATCGCATTTCGCTACGTTCTTCATCGATGCAAG
AGCCGAGATATCCGTTGCCGAGAGTCATTCTTTATAATACATATCACGTATTACCCCACA
CAATCTTATGTTAGATAGATGGGAAAACGTTTCATTTATCACAATTCCTTGGCACAATCC
GTGCCGGGGTTGGTTATCTTGGCCACATGGGCCAGTCGAGAGGAGATGGAGCAAATGA
CACCAAGCCCCCTCAACACATTTGTAAACATGTTCACAGGTTGTTCTGCTTTGCAGGAT
TCGA

S73-*DR*:

AATGCGTACTATTATCTTCATCCAAAGGTTTGCAGTACAGATGCAACCAACTCTAATGA
AATGCCACATGATGACTTAACGAGGGCAAGCCAAGGCAAACCACCGCTCATCATGAAA
TACATGACATTAATGGTTACTCTATTTCAACCAACCCACACTACTATGAGTACGGGAGAG
CCATATCAGTCCTACACCAATTACTGTTGTAGAGCCTACAATGCGTGACACCCAGGCAG
ACGTGCCCTCGACCAAAAGGCTTGGGGCGCAACTTGCGTTCAAAGACTCGATGGTTC
ACGGGATTCTGCAATTCACACCAAGTATCGCATTTCGCTACGTTCTTCATCGATGCAAG
AGCCGAGATATCCGTTGCCGAGAGTCATTCTTTATAATACATATCACGTATTACCCCACA
CAATCTTATGTTAGATAGATGGGAAAACGTTTCATTTATCACAATTCCTTGGCACAATCC
GTGCCGGGGTTGGTTATCTTGGCCACATGGGCCAGTCGAGAGGAGATGGAGCAAATGA
CACCAAGCCCCCTCAACACATTTGTAAACATGTTCACAGGTTGTTCTGCTTTGCAGGAT
TCGA

S74-*DR*:

AATGCGTACTATTATCTTCATCCAAAGGTTTGCAGTACAGATGCAACCAACTCTAATGA
AATGCCACATGATGACTTAACGAGGGCAAGCCAAGGCAAACCACCGCTCATCATGAAA

TACATGACATTAATGGTTACTCTATTTCAACCAACCACACTACTATGAGTACGGGAGAG
CCATATCAGTCCTACACCAATTACTGTTGTAGAGCCTACAATGCGTGACACCCAGGCAG
ACGTGCCCTCGACCAAAAGGCTTGGGGCGCAACTTGCGTTCAAAGACTCGATGGTTC
ACGGGATTCTGCAATTCACACCAAGTATCGCATTTCGCTACGTTCTTCATCGATGCAAG
AGCCGAGATATCCGTTGCCGAGAGTCATTCTTTATAATACATATCACGTATTACCCCACA
CAATCTTATGTTAGATAGATGGGAAAACGTTTCATTTATCACAATTCCTTGGCACAATCC
GTGCCGGGGTTGGTTATCTTGGCCACATGGGCCAGTCGAGAGGAGATGGAGCAAATGA
CACCAAGCCCCCTCAACACATTTGTAAACATGTTCACAGGTTGTTCTGCTTTGCAGGAT
TCGA

S75-*DR*:

AATGCGTACTATTATCTTCATCCAAAGGTTTGCAGTACAGATGCAACCAACTCTAATGA
AATGCCACATGATGACTTAACGAGGGCAAGCCAAGGCAAACCACCGCTCATCATGAAA
TACATGACATTAATGGTTACTCTATTTCAACCAACCNCACTACTATGAGTACGGGAGAG
CCATATCAGTCCTACACCAATTACTGTTGTAGAGCCTACAATGCGTGACACCCAGGCAG
ACGTGCCCTCGACCAAAAGGCTTGGGGCGCAACTTGCGTTCAAAGACTCGATGGTTC
ACGGGATTCTGCAATTCACACCAAGTATCGCATTTCGCTACGTTCTTCATCGATGCAAG
AGCCGAGATATCCGTTGCCGAGAGTCATTCTTTATAATACATATCACGTATTACCCCACA
CAATCTTATGTTAGATAGATGGGAAAACGTTTCATTTATCACAATTCCTTGGCACAATCC
GTGCCGGGGTTGGTTATCTTGGCCACATGGGCCAGTCGAGAGGAGATGGAGCAAATGA
CACCAAGCCCCCTCAACACATTTGTAAACATGTTCACAGGTTGTTCTGCTTTGCAGGAT
TCGA

S76-*DR*:

AATGCGTACTATTATCTTCATCCAAAGGTTTGCAGTACAGATGCAACCAACTCTAATGA
AATGCCACATGATGACTTAACGAGGGCAAGCCAAGGCAAACCACCGCTCATCATGAAA
TACATGACATTAATGGTTACTCTATTTCAACCAACCACACTACTATGAGTACGGGAGAG
CCATATCAGTCCTACACCAATTACTGTTGTAGAGCCTACAATGCGTGACACCCAGGCAG
ACGTGCCCTCGACCAAAAGGCTTGGGGCGCAACTTGCGTTCAAAGACTCGATGGTTC
ACGGGATTCTGCAATTCACACCAAGTATCGCATTTCGCTACGTTCTTCATCGATGCAAG
AGCCGAGATATCCGTTGCCGAGAGTCATTCTTTATAATACATATCACGTATTACCCCACA
CAATCTTATGTTAGATAGATGGGAAAACGTTTCATTTATCACAATTCCTTGGCACAATCC
GTGCCGGGGTTGGTTATCTTGGCCACATGGGCCAGTCGAGAGGAGATGGAGCAAATGA
CACCAAGCCCCCTCAACACATTTGTAAACATGTTCACAGGTTGTTCTGCTTTGCAGGAT
TCGA

S77-*DR*:

AATGCGTACTATTATCTTCATCCAAAGGTTTGCAGTACAGATGCAACCAACTCTAATGA
AATGCCACATGATGACTTAACGAGGGCAAGCCAAGGCAAACCACCGCTCATCATGAAA
TACATGACATTAATGGTTACTCTATTTCAACCAACCNCACTACTATGAGTACGGGAGAG
CCATATCAGTCCTACACCAATTACTGTTGTAGAGCCTACAATGCGTGACACCCAGGCAG
ACGTGCCCTCGACCAAAAGGCTTGGGGCGCAACTTGCGTTCAAAGACTCGATGGTTC
ACGGGATTCTGCAATTCACACCAAGTATCGCATTTCGCTACGTTCTTCATCGATGCAAG
AGCCGAGATATCCGTTGCCGAGAGTCATTCTTTATAATACATATCACGTATTACCCCACA
CAATCTTATGTTAGATAGATGGGAAAACGTTTCATTTATCACAATTCCTTGGCACAATCC
GTGCCGGGGTTGGTTATCTTGGCCACATGGGCCAGTCGAGAGGAGATGGAGCAAATGA
CACCAAGCCCCCTCAACACATTTGTAAACATGTTCACAGGTTGTTCTGCTTTGCAGGAT

TCGA
S78-*DR*:
AATGCGTACTATTATCTTCATCCAAAGGTTTGCAGTACAGATGCAACCAACTCTAATGA
AATGCCACATGATGACTTAACGAGGGCAAGCCAAGGCAAACCACCGCTCATCATGAAA
TACATGACATTAATGGTTACTCTATTTCAACCAACCNCACTACTATGAGTACGGGAGAG
CCATATCAGTCCTACACCAATTACTGTTGTAGAGCCTACAATGCGTGACACCCAGGCAG
ACGTGCCCTCGACCAAAAGGCTTGGGGCGCAACTTGCGTTCAAAGACTCGATGGTTC
ACGGGATTCTGCAATTCACACCAAGTATCGCATTTCGCTACGTTCTTCATCGATGCAAG
AGCCGAGATATCCGTTGCCGAGAGTCATTCTTTATAATACATATCACGTATTACCCCACA
CAATCTTATGTTAGATAGATGGGAAAACGTTTCATTTATCACAATTCCTTGGCACAATCC
GTGCCGGGGTTGGTTATCTTGGCCACATGGGCCAGTCGAGAGGAGATGGAGCAAATGA
CACCAAGCCCCCTCAACACATTTGTAAACATGTTCACAGGTTGTTCTGCTTTGCAGGAT
TCGA
S79-*DT*:
AATGCGTACTATTATCTTCATCCAAAGGTTTGCAGTACAGATGCAACCAACTCTAATGA
CATGCCACATGATGACTTAACGAGGGCAAGCCAAGGCAAACCACCGCTCATCATGAAA
TACATGACATTAATGGTTACTCTATTTCAACCAACCGCACTACTATGAGTACGGGAGAG
CCATATCAGTCCTACACCAATTACTGTTGTAGAGCCTACAATGCGTGACACCCAGGCAG
ACGTGCCCTCGACCAAAAGGCTTGGGGCGCAACTTGCGTTCAAAGACTCGATGGTTC
ACGGGATTCTGCAATTCACACCAAGTATCGCATTTCGCTACGTTCTTCATCGATGCAAG
AGCCGAGATATCCGTTGCCGAGAGTCATTCTTTATAATACATATCACGTATTACCCCACA
CAATCTTATGTTAGATAGATGGGAAAACGTTTCATTTATCACAATTCCTTGGCACAATCC
GCGCCGGGGTTGGTTATCTTGGCCACATGGGCCAGTCGAGAGGAGATGAAGCAAATGA
CACCAAGCCCCCTCAACACATTTGTAAACATGTTCACAGGTTGTTCTGCTTTGCAGGAT
TCGA
S80-*DT*:
AATGCGTACTATTATCTTCATCCAAAGGTTTGCAGTACAGATGCAACCAACTCTAATGA
CATGCCACATGATGACTTAACGAGGGCAAGCCAAGGCAAACCACCGCTCATCATGAAA
TACATGACATTAATGGTTACTCTATTTCAACCAACCGCACTACTATGAGTACGGGAGAG
CCATATCAGTCCTACACCAATTACTGTTGTAGAGCCTACAATGCGTGACACCCAGGCAG
ACGTGCCCTCGACCAAAAGGCTTGGGGCGCAACTTGCGTTCAAAGACTCGATGGTTC
ACGGGATTCTGCAATTCACACCAAGTATCGCATTTCGCTACGTTCTTCATCGATGCAAG
AGCCGAGATATCCGTTGCCGAGAGTCATTCTTTATAATACATATCACGTATTACCCCACA
CAATCTTATGTTAGATAGATGGGAAAACGTTTCATTTATCACAATTCCTTGGCACAATCC
GCGCCGGGGTTGGTTATCTTGGCCACATGGGCCAGTCGAGAGGAGATGAAGCAAATGA
CACCAAGCCCCCTCAACACATTTGTAAACATGTTCACAGGTTGTTCTGCTTTGCAGGAT
TCGA
S81-*DT*:
AATGCGTACTATTATCTTCATCCAAAGGTTTGCAGTACAGATGCAACCAACTCTAATGA
CATGCCACATGATGACTTAACGAGGGCAAGCCAAGGCAAACCACCGCTCATCATGAAA
TACATGACATTAATGGTTACTCTATTTCAACCAACCGCACTACTATGAGTACGGGAGAG
CCATATCAGTCCTACACCAATTACTGTTGTAGAGCCTACAATGCGTGACACCCAGGCAG
ACGTGCCCTCGACCAAAAGGCTTGGGGCGCAACTTGCGTTCAAAGACTCGATGGTTC
ACGGGATTCTGCAATTCACACCAAGTATCGCATTTCGCTACGTTCTTCATCGATGCAAG

AGCCGAGATATCCGTTGCCGAGAGTCATTCTTTATAATACATATCACGTATTACCCCACA
CAATCTTATGTTAGATAGATGGGAAAACGTTTCATTTATCACAATTCCTTGGCACAATCC
GCGCCGGGGTTGGTTATCTTGGCCACATGGGCCAGTCGAGAGGAGATGAAGCAAATGA
CACCAAGCCCCCTCAACACATTTGTAAACATGTTCACAGGTTGTTCTGCTTTGCAGGAT
TCGA

S82-*DT*:

AATGCGTACTATTATCTTCATCCAAAGGTTTGCAGTACAGATGCAACCAACTCTAATGA
CATGCCACATGATGACTTAACGAGGGCAAGCCAAGGCAAACCACCGCTCATCATGAAA
TACATGACATTAATGGTTACTCTATTTCAACCAACCGCACTACTATGAGTACGGGAGAG
CCATATCAGTCCTACACCAATTACTGTTGTAGAGCCTACAATGCGTGACACCCAGGCAG
ACGTGCCCTCGACCAAAAGGCTTGGGGCGCAACTTGCGTTCAAAGACTCGATGGTTC
ACGGGATTCTGCAATTCACACCAAGTATCGCATTTCGCTACGTTCTTCATCGATGCAAG
AGCCGAGATATCCGTTGCCGAGAGTCATTCTTTATAATACATATCACGTATTACCCCACA
CAATCTTATGTTAGATAGATGGGAAAACGTTTCATTTATCACAATTCCTTGGCACAATCC
GCGCCGGGGTTGGTTATCTTGGCCACATGGGCCAGTCGAGAGGAGATGAAGCAAATGA
CACCAAGCCCCCTCAACACATTTGTAAACATGTTCACAGGTTGTTCTGCTTTGCAGGAT
TCGA

S83-*DT*:

AATGCGTACTATTATCTTCATCCAAAGGTTTGCAGTACAGATGCAACCAACTCTAATGA
CATGCCACATGATGACTTAACGAGGGCAAGCCAAGGCAAACCACCGCTCATCATGAAA
TACATGACATTAATGGTTACTCTATTTCAACCAACCGCACTACTATGAGTACGGGAGAG
CCATATCAGTCCTACACCAATTACTGTTGTAGAGCCTACAATGCGTGACACCCAGGCAG
ACGTGCCCTCGACCAAAAGGCTTGGGGCGCAACTTGCGTTCAAAGACTCGATGGTTC
ACGGGATTCTGCAATTCACACCAAGTATCGCATTTCGCTACGTTCTTCATCGATGCAAG
AGCCGAGATATCCGTTGCCGAGAGTCATTCTTTATAATACATATCACGTATTACCCCACA
CAATCTTATGTTAGATAGATGGGAAAACGTTTCATTTATCACAATTCCTTGGCACAATCC
GCGCCGGGGTTGGTTATCTTGGCCACATGGGCCAGTCGAGAGGAGATGAAGCAAATGA
CACCAAGCCCCCTCAACACATTTGTAAACATGTTCACAGGTTGTTCTGCTTTGCAGGAT
TCGA

S84-*DT*:

AATGCGTACTATTATCTTCATCCAAAGGTTTGCAGTACAGATGCAACCAACTCTAATGA
CATGCCACATGATGACTTAACGAGGGCAAGCCAAGGCAAACCACCGCTCATCATGAAA
TACATGACATTAATGGTTACTCTATTTCAACCAACCGCACTACTATGAGTACGGGAGAG
CCATATCAGTCCTACACCAATTACTGTTGTAGAGCCTACAATGCGTGACACCCAGGCAG
ACGTGCCCTCGACCAAAAGGCTTGGGGCGCAACTTGCGTTCAAAGACTCGATGGTTC
ACGGGATTCTGCAATTCACACCAAGTATCGCATTTCGCTACGTTCTTCATCGATGCAAG
AGCCGAGATATCCGTTGCCGAGAGTCATTCTTTATAATACATATCACGTATTACCCCACA
CAATCTTATGTTAGATAGATGGGAAAACGTTTCATTTATCACAATTCCTTGGCACAATCC
GCGCCGGGGTTGGTTATCTTGGCCACATGGGCCAGTCGAGAGGAGATGNAGCAAATGA
CACCAAGCCCCCTCAACACATTTGTAAACATGTTCACAGGTTGTTCTGCTTTGCAGGAT
TCGA

S85-*DT*:

AATGCGTACTATTATCTTCATCCAAAGGTTTGCAGTACAGATGCAACCAACTCTAATGA
CATGCCACATGATGACTTAACGAGGGCAAGCCAAGGCAAACCACCGCTCATCATGAAA

TACATGACATTAATGGTTACTCTATTTCAACCAACCGCACTACTATGAGTACGGGAGAG
CCATATCAGTCCTACACCAATTACTGTTGTAGAGCCTACAATGCGTGACACCCAGGCAG
ACGTGCCCTCGACCAAAAGGCTTGGGGCGCAACTTGCGTTCAAAGACTCGATGGTTC
ACGGGATTCTGCAATTCACACCAAGTATCGCATTTCGCTACGTTCTTCATCGATGCAAG
AGCCGAGATATCCGTTGCCGAGAGTCATTCTTTATAATACATATCACGTATTACCCCACA
CAATCTTATGTTAGATAGATGGGAAAACGTTTCATTTATCACAATTCCTTGGCACAATCC
GCGCCGGGGTTGGTTATCTTGGCCACATGGGCCAGTCGAGAGGAGATGGAGCAAATGA
CACCAAGCCCCCTCAACACATTTGTAAACATGTTCACAGGTTGTTCTGCTTTGCAGGAT
TCGA

S86-*DT*:

AATGCGTACTATTATCTTCATCCAAAGGTTTGCAGTACAGATGCAACCAACTCTAATGA
CATGCCACATGATGACTTAACGAGGGCAAGCCAAGGCAAACCACCGCTCATCATGAAA
TACATGACATTAATGGTTACTCTATTTCAACCAACCGCACTACTATGAGTACGGGAGAG
CCATATCAGTCCTACACCAATTACTGTTGTAGAGCCTACAATGCGTGACACCCAGGCAG
ACGTGCCCTCGACCAAAAGGCTTGGGGCGCAACTTGCGTTCAAAGACTCGATGGTTC
ACGGGATTCTGCAATTCACACCAAGTATCGCATTTCGCTACGTTCTTCATCGATGCAAG
AGCCGAGATATCCGTTGCCGAGAGTCATTCTTTATAATACATATCACGTATTACCCCACA
CAATCTTATGTTAGATAGATGGGAAAACGTTTCATTTATCACAATTCCTTGGCACAATCC
GCGCCGGGGTTGGTTATCTTGGCCACATGGGCCAGTCGAGAGGAGATGGAGCAAATGA
CACCAAGCCCCCTCAACACATTTGTAAACATGTTCACAGGTTGTTCTGCTTTGCAGGAT
TCGA

S87-*DT*:

AATGCGTACTATTATCTTCATCCAAAGGTTTGCAGTACAGATGCAACCAACTCTAATGA
CATGCCACATGATGACTTAACGAGGGCAAGCCAAGGCAAACCACCGCTCATCATGAAA
TACATGACATTAATGGTTACTCTATTTCAACCAACCGCACTACTATGAGTACGGGAGAG
CCATATCAGTCCTACACCAATTACTGTTGTAGAGCCTACAATGCGTGACACCCAGGCAG
ACGTGCCCTCGACCAAAAGGCTTGGGGCGCAACTTGCGTTCAAAGACTCGATGGTTC
ACGGGATTCTGCAATTCACACCAAGTATCGCATTTCGCTACGTTCTTCATCGATGCAAG
AGCCGAGATATCCGTTGCCGAGAGTCATTCTTTATAATACATATCACGTATTACCCCACA
CAATCTTATGTTAGATAGATGGGAAAACGTTTCATTTATCACAATTCCTTGGCACAATCC
GCGCCGGGGTTGGTTATCTTGGCCACATGGGCCAGTCGAGAGGAGATGAAGCAAATGA
CACCAAGCCCCCTCAACACATTTGTAAACATGTTCACAAGTTGTTCTGCTTTGCAGGAT
TCGA

S88-*DT*:

AATGCGTACTATTATCTTCATCCAAAGGTTTGCAGTACAGATGCAACCAACTCTAATGA
CATGCCACATGATGACTTAACGAGGGCAAGCCAAGGCAAACCACCGCTCATCATGAAA
TACATGACATTAATGGTTACTCTATTTCAACCAACCGCACTACTATGAGTACGGGAGAG
CCATATCAGTCCTACACCAATTACTGTTGTAGAGCCTACAATGCGTGACACCCAGGCAG
ACGTGCCCTCGACCAAAAGGCTTGGGGCGCAACTTGCGTTCAAAGACTCGATGGTTC
ACGGGATTCTGCAATTCACACCAAGTATCGCATTTCGCTACGTTCTTCATCGATGCAAG
AGCCGAGATATCCGTTGCCGAGAGTCATTCTTTATAATACATATCACGTATTACCCCACA
CAATCTTATGTTAGATAGATGGGAAAACGTTTCATTTATCACAATTCCTTGGCACAATCC
GCGCCGGGGTTGGTTATCTTGGCCACATGGGCCAGTCGAGAGGAGATGAAGCAAATGA
CACCAAGCCCCCTCAACACATTTGTAAACATGTTCACAAGTTGTTCTGCTTTGCAGGAT

TCGA
S89-*DT*:
AATGCGTACTATTATCTTCATCCAAAGGTTTGCAGTACAGATGCAACCAACTCTAATGA
CATGCCACATGATGACTTAACGAGGGCAAGCCAAGGCAAACCACCGCTCATCATGAAA
TACATGACATTAATGGTTACTCTATTTCAACCAACCGCACTACTATGAGTACGGGAGAG
CCATATCAGTCCTACACCAATTACTGTTGTAGAGCCTACAATGCGTGACACCCAGGCAG
ACGTGCCCTCGACCAAAAGGCTTGGGGCGCAACTTGCGTTCAAAGACTCGATGGTTC
ACGGGATTCTGCAATTCACACCAAGTATCGCATTTCGCTACGTTCTTCATCGATGCAAG
AGCCGAGATATCCGTTGCCGAGAGTCATTCTTTATAATACATATCACGTATTACCCCACA
CAATCTTATGTTAGATAGATGGGAAAACGTTTCATTTATCACAATTCCTTGGCACAATCC
GCGCCGGGGTTGGTTATCTTGGCCACATGGGCCAGTCGAGAGGAGATGAAGCAAATGA
CACCAAGCCCCCTCAACACATTTGTAAACATGTTCACAAGTTGTTCTGCTTTGCAGGAT
TCGA
S90-*DT*:
AATGCGTACTATTATCTTCATCCAAAGGTTTGCAGTACAGATGCAACCAACTCTAATGA
CATGCCACATGATGACTTAACGAGGGCAAGCCAAGGCAAACCACCGCTCATCATGAAA
TACATGACATTAATGGTTACTCTATTTCAACCAACCGCACTACTATGAGTACGGGAGAG
CCATATCAGTCCTACACCAATTACTGTTGTAGAGCCTACAATGCGTGACACCCAGGCAG
ACGTGCCCTCGACCAAAAGGCTTGGGGCGCAACTTGCGTTCAAAGACTCGATGGTTC
ACGGGATTCTGCAATTCACACCAAGTATCGCATTTCGCTACGTTCTTCATCGATGCAAG
AGCCGAGATATCCGTTGCCGAGAGTCATTCTTTATAATACATATCACGTATTACCCCACA
CAATCTTATGTTAGATAGATGGGAAAACGTTTCATTTATCACAATTCCTTGGCACAATCC
GCGCCGGGGTTGGTTATCTTGGCCACATGGGCCAGTCGAGAGGAGATGGAGCAAATGA
CACCAAGCCCCCTCAACACATTTGTAAACATGTTCACAGGTTGTTCTGCTTTGCAGGAT
TCGA
S91-*DT*:
AATGCGTACTATTATCTTCATCCAAAGGTTTGCAGTACAGATGCAACCAACTCTAATGA
CATGCCACATGATGACTTAACGAGGGCAAGCCAAGGCAAACCACCGCTCATCATGAAA
TACATGACATTAATGGTTACTCTATTTCAACCAACCGCACTACTATGAGTACGGGAGAG
CCATATCAGTCCTACACCAATTACTGTTGTAGAGCCTACAATGCGTGACACCCAGGCAG
ACGTGCCCTCGACCAAAAGGCTTGGGGCGCAACTTGCGTTCAAAGACTCGATGGTTC
ACGGGATTCTGCAATTCACACCAAGTATCGCATTTCGCTACGTTCTTCATCGATGCAAG
AGCCGAGATATCCGTTGCCGAGAGTCATTCTTTATAATACATATCACGTATTACCCCACA
CAATCTTATGTTAGATAGATGGGAAAACGTTTCATTTATCACAATTCCTTGGCACAATCC
GCGCCGGGGTTGGTTATCTTGGCCACATGGGCCAGTCGAGAGGAGATGGAGCAAATGA
CACCAAGCCCCCTCAACACATTTGTAAACATGTTCACAGGTTGTTCTGCTTTGCAGGAT
TCGA
S92-*DT*:
AATGCGTACTATTATCTTCATCCAAAGGTTTGCAGTACAGATGCAACCAACTCTAATGA
CATGCCACATGATGACTTAACGAGGGCAAGCCAAGGCAAACCACCGCTCATCATGAAA
TACATGACATTAATGGTTACTCTATTTCAACCAACCGCACTACTATGAGTACGGGAGAG
CCATATCAGTCCTACACCAATTACTGTTGTAGAGCCTACAATGCGTGACACCCAGGCAG
ACGTGCCCTCGACCAAAAGGCTTGGGGCGCAACTTGCGTTCAAAGACTCGATGGTTC
ACGGGATTCTGCAATTCACACCAAGTATCGCATTTCGCTACGTTCTTCATCGATGCAAG

AGCCGAGATATCCGTTGCCGAGAGTCATTCTTTATAATACATATCACGTATTACCCCACA
CAATCTTATGTTAGATAGATGGGAAAACGTTTCATTTATCACAATTCCTTGGCACAATCC
GCGCCGGGGTTGGTTATCTTGGCCACATGGGCCAGTCGAGAGGAGATGGAGCAAATGA
CACCAAGCCCCCTCAACACATTTGTAAACATGTTCACAGGTTGTTCTGCTTTGCAGGAT
TCGA

S93-*DT*:

AATGCGTACTATTATCTTCATCCAAAGGTTTGCAGTACAGATGCAACCAACTCTAATGA
CATGCCACATGATGACTTAACGAGGGCAAGCCAAGGCAAACCACCGCTCATCATGAAA
TACATGACATTAATGGTTACTCTATTTCAACCAACCGCACTACTATGAGTACGGGAGAG
CCATATCAGTCCTACACCAATTACTGTTGTAGAGCCTACAATGCGTGACACCCAGGCAG
ACGTGCCCTCGACCAAAAGGCTTGGGGCGCAACTTGCGTTCAAAGACTCGATGGTTC
ACGGGATTCTGCAATTCACACCAAGTATCGCATTTCGCTACGTTCTTCATCGATGCAAG
AGCCGAGATATCCGTTGCCGAGAGTCATTCTTTATAATACATATCACGTATTACCCCACA
CAATCTTATGTTAGATAGATGGGAAAACGTTTCATTTATCACAATTCCTTGGCACAATCC
GCGCCGGGGTTGGTTATCTTGGCCACATGGGCCAGTCGAGAGGAGATGNAGCAAATGA
CACCAAGCCCCCTCAACACATTTGTAAACATGTTCACAGGTTGTTCTGCTTTGCAGGAT
TCGA

Thymelaea aucheri-AJ549445.1

GATGCGTGCTATCTTCATCCAAAAGTCTGAAGTAGAGAAGCAACCAACTCTAGTGATG
TGCTGCACACGATGACTTAACGAGGGCAAGCCAAGACAAACCACCGCTCATCGTGGC
ATACACCACATTAAGGTTACTCTATTTGGGCCAACCGCACTACTGTGAGTACGGGAGAG
CCAATATCAGCCCACACAACTATTGTAAAGGCTACGATGCGTGACACCCAGGCAGACG
TGCCCTCGGCCCGAAGGCTTGGGGCGCAACTTGCGTTCAAAGACTCGATGGTTCACGG
GATTCTGCAATTCACACCAAGTATCGCATTTCGCTACGTTCTTCATCGATGCGAGAGCC
GAGATATCCGTTGCCGAGAGTCGTTCTGTGTAATACATATCACGCATTACCCCACACAAT
CTTGTGTTAAGATGTATGGGGGTAAAAACGTTTCATTTGTCACAATTCCTTGGCGCATTC
CACGCCGGGGTTGGTTATCTTGGCCACATGGGCCAGTCAAGTGAAAATGGAGCCAATG
GCTACATTCCCCCTTAACACATTTACTAACATGTTCACGGGTTGTTCTGCTTTGCAGGAT
TCGA

Thymelaea dioica-AJ549468.1

GATGCGCACTGTCTTCGTTCGAAAGTCTGAAGTACAAAAGCAACCAACTTTAGTGATG
TGCTGCACACGATGACTTAACGAGGGCAAGCCAAGACAAACCACCGCTCATCGTGGC
ATACACCACATTAGGGTTACTCTATTTGGGCCAACCGTACTACTATGAGTATGGGAGAG
CCAATATCAGCACCACACCAACAGCTGTTGTATGGGCTACGATGCGTGACACCCAGGC
AGACGTGCCCTCGGCCCGAAGGCTTGGGGCGCAACTTGCGTTCAAAGACTCGATGGT
TCACGGGATTCTGCAATTCACACCAAGTATCGCATTTCGCTACGTTCTTCATCGATGCG
AGAGCCGAGATATCCGTTGCCGAGAGTCGTTCTTTATAATACATATCACGCATTACCCCA
CACAATCCTGTGTTGGATGCATGAGGAAAATGCTTTATTTATCACAATTCCTTGGCGCAA
TCCGCGCCGGGGTTGGTTATCTTGGCCACATGGGCCAGTCAAGAAGAAATGGAGCCAA
AGGCTGCATTCCCCCTCAACACATTTGCTAACATGTTCACAGGTTGTTCTGCTTTGCAG
GATTCGA

Daphne blagayana isolate DB45_SL-GQ167491.1

AGCGGGTATTCCCGCCTGACCTGGGGTCGCAATGCGTACTATCTTCAACCAAAGGTTTG
AAGTACAATGCAACCAACTCTAATGACATGCTATACACGATGACTTAACGAGGGCAAG

CGAAGACAAACCACCGCTCATCATGAAATACATGACATTAAAGGTTACTCAATTTCAAC
CAACCGCACTACTATGAGGACGGGAGAGCCATTCTCAGCCCTACACAGACTAATCTTGT
AGAGGCTACAATGCGTGACACCCAGGCAGACGTGCCCTCGGCCCGAAGGCTTGGGGC
GCAACTTGCGTTCAAAGACTCGATGGTTCACGGGATTCTGCAATTCACACCAAGTATC
GCATTTCGCTACGTTCTTCATCGATGCGAGAGCCGAGATATCCGTTGCCGAGAGTCATT
CTTTATAATACATATCACGCATTACCCCATACAATCTAATGTTAGATGCATGGGGAAATCG
TTTCATTTTTCAAAATTCCTTGGCACAATCCGCGCCGGGGTTGGTTATCTTGGCCACATG
AGCCAGTTGAGAGGAGATGGAGCCAATGACACCAAGCCCCCTCAACACATTTGCAAA

Daphne cneorum-AJ549490.1

AATGCGTAATATTATCTTCATCCAAAGGTTTGAAGTACAAATGCAACCAACACCAATGA
CATGCTATACACGATGACTTAACGAAGGCAAGCCAAGACAAACCACCGCTCATCATGA
AATACATGACATTAATGGTTACCCTATTTCAGCCAACCGCACTACTATGAGGACGGGAG
AGCCATATCAGCCCTACACCCACAAATTTTGTAGAGGCTACAATGCGTGACACCCAGG
CAGACGTGCCCTCAACCCGAAGGCTTGGGGCGCAACTTGCGTTCAAAGACTCGATGG
TTCACGGGATTCTGCAATTCACACCAAGTATCGCATTTCGCTACGTTCTTCATCGATGCA
AGAGCCGAGATATCCGTTGCCGAGAGTCATTCTTTATAATATACATATCACGCATTACCA
CACACAATCTTATGTTAGATGGCATGGGGAAAACGTTTCATTTATCACAATTCCTTGGCA
CAATCCGTGCCGGGGTTGGTTATCTTGGCCACATGGGCCAGTCAAGAGGAGATGGAGC
CAATGACGCCAAAGCCCCCCTCAACACATTTGAAAACATGTTCACAAGTTGTTTTGCT
TTGCAGGATTCGA
