# Supplementary material for: Identification of three Daphne species by DNA barcoding and HPLC fingerprint analysis
Source: PLoS One. 2018 Aug 2;13(8):e0201711. doi: 10.1371/journal.pone.0201711 (PMC6072044; doi:10.1371/journal.pone.0201711)
Supplement: S3 File — (PDF) [file pone.0201711.s003.pdf]

S3 File. Results of HCA and PCA of Cortex Daphnes samples

	1	2	3	4	5	6	7	8
S1	0.187716	0.053503	1.345998	0.052341	1	0	0.151783	0.019595
S2	0.18442	0.072252	1.269893	0.050212	1	0	0.162563	0.016417
S3	0.180246	0.04221	1.071071	0.048657	1	0	0.155054	0.022287
S4	0.172507	0.045406	1.155301	0.052668	1	0	0.156586	0.000001
S5	0.000001	0.071355	1.322275	0.048961	1	0	0.157468	0.022353
S6	0.170837	0.07811	1.890428	0.058392	1	0	0.163757	0.011997
S7	0.179226	0.039599	1.901266	0.064138	1	0	0.182314	0.020951
S8	0.193165	0.069402	1.665088	0.059303	1	0	0.168761	0.010694
S9	0.16021	0.050492	0.000001	0.04105	1	0	0.204238	0.011541
S10	0.19755	0.069567	1.500762	0.053873	1	0	0.213719	0.010783
S11	0.117702	0.055881	2.45476	0.048888	1	0	0.161133	0.01438
S12	0.108642	0.078742	2.218569	0.057771	1	0	0.171871	0.011232
S13	0.123813	0.05434	1.518283	0.053802	1	0	0.200263	0.010945
S14	0.154104	0.062137	2.361631	0.053054	1	0	0.18299	0.014777
S15	0.141147	0.062379	0.947921	0.036815	1	0	0.163338	0.000001
S16	0.152735	0.037749	1.895825	0.035512	1	0	0.157567	0.015889
S17	0.167651	0.028709	1.064802	0.035605	1	0	0.185502	0.024482
S18	0.191315	0.071748	1.33116	0.047022	1	0	0.329292	0.011184
S19	0.169589	0.064303	1.1913	0.044783	1	0	0.176287	0.020804
S20	0.216587	0.058955	1.347842	0.0391	1	0	0.206953	0.016456
S21	0.238464	0.059855	1.252369	0.076207	1	0	0.284149	0.027341
S22	0.190263	0.054242	1.274198	0.0694	1	0	0.354975	0.023501
S23	0.210515	0.069296	1.278222	0.052746	1	0	0.316624	0.029121
S24	0.210515	0.068296	1.268222	0.042746	1	0	0.416624	0.026121
S25	0.192162	0.203598	1.748244	0.05352	1	0.02015	0.133465	0.074919
S26	0.215557	0.193479	1.777034	0.073197	1	0.019266	0.301609	0.000001
S27	0.00001	0.215608	1.715421	0.064787	1	0.019886	0.209079	0.080522
S28	0.201266	0.184301	1.674433	0.070845	1	0.020604	0.145575	0.071243
S29	0.262932	0.000001	1.606454	0.052799	1	0.02401	0.480926	0.065901
S30	0.20232	0.208942	1.753198	0.028995	1	0.021163	0.499495	0.064569
S31	0.208308	0.195492	1.175852	0.080326	1	0.020193	0.509971	0.065187
S32	0.000001	0.190232	1.604611	0.044301	1	0	0.608253	0.081081
S33	0.21648	0.207284	0.000011	0.034953	1	0.022245	0.493381	0.054157
S34	0.226202	0.184564	1.74822	0.036209	1	0.023826	0.000001	0.065788
S35	0.222062	0.178812	1.667506	0.039399	1	0.023552	0.217449	0.072021
S36	0.221398	0.213564	1.815235	0.062685	1	0	0.395669	0.050394
S37	0.000001	0.213729	1.704676	0.000001	1	0.021288	0.572874	0.069577
S38	0.236565	0.229977	1.806167	0.047013	1	0.021438	0.474856	0.06586
S39	0.166504	0.114069	1.316991	0.108989	1	0	0.000001	0.01757
S40	0.120405	0.104518	0.000001	0.101269	1	0	0.162491	0.015937
S41	0.000001	0.098463	1.090637	0.10011	1	0	0.193164	0.014153
S42	0.10286	0.105799	1.052149	0.099288	1	0	0.180306	0.015525
S43	0.130113	0.000001	1.367849	0.110691	1	0	0.121228	0.022765
S44	0.214128	0.141038	1.311627	0.132197	1	0	0.184009	0.015547
S45	0.196633	0.173378	1.978535	0.000001	1	0	0.201759	0.018873
S46	0.149467	0.142974	1.455467	0.109967	1	0	0.164284	0.016651
S47	0.082856	0.1444	1.150117	0.076323	1	0	0.157917	0.00893
S48	0.000001	0.122151	1.083127	0.041083	1	0	0.102147	0.000001
S49	0.102512	0.14082	1.188597	0.111804	1	0	0.175523	0.013757

S50	0.096002	0.138576	1.1821	0.125137	1	0	0.337085	0.023547
S51	0.125506	0.148798	1.248169	0.109085	1	0	0.206832	0.011858
S52	0.149247	0.177933	1.513163	0.128582	1	0	0.130946	0.009354
S53	0.152429	0.155931	1.422934	0.084192	1	0	0.253435	0.014188
S54	0.166034	0.147521	1.404775	0.092917	1	0	0.000001	0.016038
S55	0.158518	0.141643	0.000001	0.126819	1	0	0.188334	0.013793
S56	0.153834	0.132741	1.131413	0.101996	1	0	0.286008	0.019999
S57	0.156186	0.156876	1.402784	0.098543	1	0	0.239138	0.016646
S58	0.254862	0.166854	2.77247	0.059756	1	0	0.262456	0.023608
S59	0.249321	0.182135	2.999996	0.063008	1	0	0.29822	0.022227
S60	0.34243	0.179188	2.962896	0.054366	1	0	0.399717	0.028117
S61	0.210691	0.216179	2.986938	0.041784	1	0	0.295812	0.026074
S62	0.423252	0.223322	2.703597	0.052173	1	0	0.305782	0.02732
S63	0.244347	0.175718	2.902419	0.06569	1	0	0.314882	0.02763
S64	0.211908	0.166816	2.846941	0.061288	1	0	0.257381	0.026838
S65	0.34243	0.179188	2.962896	0.054366	1	0	0.269972	0.028117
S66	0.12073	0.086576	1.13205	0.174249	1	0	0.104997	0.013884
S67	0.083552	0.070177	1.08451	0.087693	1	0	0.108681	0.012323
S68	0.07355	0.077269	0.932348	0.082415	1	0	0.117593	0.012871
S69	0.170267	0.073063	1.371039	0.051673	1	0	0.147451	0.014375
S70	0.055225	0.257434	0.807893	0.120794	1	0	0.101812	0.025435
S71	0.037322	0.232717	0.803859	0.180487	1	0	0.093927	0.012485
S72	0.024995	0.188101	0.61394	0.108681	1	0	0.161345	0.012966
S73	0.058659	0.262382	0.882955	0.167702	1	0	0.095001	0.013368
S74	0.052786	0.244603	0.759842	0.116456	1	0	0.116962	0.017587
S75	0.16809	0.088518	1.470186	0.057509	1	0	0.138139	0.015724
S76	0.128968	0.078438	1.364155	0.045709	1	0	0.122216	0.014212
S77	0.068021	0.135546	0.70981	0.072218	1	0	0.089998	0.020981
S78	0.149382	0.139345	1.664049	0.124075	1	0	0.17549	0.014262
S79	0.055225	0.257434	0.807893	0.120794	1	0	0.101812	0.025435
S80	0.037322	0.232717	0.803859	0.180487	1	0	0.093927	0.012485
S81	0.024995	0.188101	0.61394	0.108681	1	0	0.161345	0.012966
S82	0.058659	0.262382	0.882955	0.167702	1	0	0.095001	0.013368
S83	0.052786	0.244603	0.759842	0.116456	1	0	0.116962	0.017587
S84	0.320915	0.300913	1.694436	0.066231	1	0	0.0902	0.019209
S85	0.300915	0.330091	1.769444	0.066231	1	0	0.0942	0.020209
S86	0.310915	0.303009	1.739444	0.000001	1	0	0.0932	0.020921
S87	0.321111	0.31815	1.781515	0.069569	1	0	0.112	0.017004
S88	0.341111	0.29815	1.845152	0.068826	1	0	0.11	0.022004
S89	0.331111	0.32815	1.815152	0.069763	1	0	0.099998	0.021004
S90	0.343109	0.362918	2.19233	0.059273	1	0	0.079993	0.036561
S91	0.32611	0.303494	1.852057	0.000001	1	0	0.085014	0.024926
S92	0.32711	0.343494	1.952057	0.055116	1	0	0.084501	0.034926
S93	0.32	0.222857	1.97	0.051429	1	0	0.085714	0.021429

9	10	11	12	13	14	15	16	17
0	0	0.008604	0.019838	0	0	0	0.009479	0
0	0	0.008946	0	0	0	0	0.008603	0
0	0	0.009425	0.021796	0	0	0	0.008789	0
0	0	0.00813	0.021159	0	0	0	0.008158	0
0	0	0.000001	0.021201	0	0	0	0	0
0	0	0.009497	0.014854	0	0	0	0.00957	0
0	0	0.009142	0.023649	0	0	0	0.006942	0
0	0	0.009837	0.025892	0	0	0	0.008679	0
0	0	0.008548	0.023029	0	0	0	0.005284	0
0	0	0.008165	0.022115	0	0	0	0.007379	0
0	0	0.008393	0.023369	0	0	0	0	0
0	0	0.00844	0.021295	0	0	0	0.009128	0
0	0	0.006793	0	0	0	0	0.007155	0
0	0	0.006327	0.01158	0	0	0	0.007093	0
0	0	0.007837	0	0	0	0	0.008433	0
0	0	0.00247	0.014151	0	0	0	0.009569	0
0	0	0.002323	0.022024	0	0	0	0.009091	0
0.157939	0	0.038129	0.007384	0	0	0.10664	0	0.065702
0.135025	0	0.015715	0.006033	0	0	0.077002	0	0.054893
0.153122	0	0.017941	0.008509	0	0	0.089708	0	0.053013
0.15936	0	0.0302	0.005028	0	0	0.051809	0	0.029091
0.190452	0	0.039695	0.004993	0	0	0.0739	0	0.041327
0.14115	0	0.031915	0.006431	0	0	0.041854	0	0.042888
0.15115	0	0.032192	0.007431	0	0	0.041854	0	0.042888
0	0	0.02062	0.00979	0	0.074572	0	0	0
0	0	0.000001	0.010294	0	0.063585	0	0	0
0	0	0.047624	0.007055	0	0.06581	0	0	0
0	0	0.024988	0.007109	0	0.06648	0	0	0
0	0	0.031435	0	0	0.078395	0	0	0
0	0	0.084609	0.008086	0	0.053599	0	0	0
0	0	0.045011	0.009176	0	0.069443	0	0	0
0	0	0.05106	0.010338	0	0.059443	0	0	0
0	0	0.000001	0.01149	0	0.048742	0	0	0
0	0	0.063145	0.009757	0	0.061591	0	0	0
0	0	0.062755	0.007807	0	0.060645	0	0	0
0	0	0.056551	0.010311	0	0.070532	0	0	0
0	0	0.000001	0.008465	0	0.053599	0	0	0
0	0	0.068131	0.010449	0	0.06443	0	0	0
0	0	0.010903	0.006523	0	0.074572	0	0	0
0	0	0.010264	0.004477	0	0.063585	0	0	0
0	0	0.009799	0.004645	0	0.06581	0	0	0
0	0	0.010632	0.004136	0	0.06648	0	0	0
0	0	0.008702	0	0	0.078395	0	0	0
0	0	0.013091	0.006231	0	0.053599	0	0	0
0	0	0.015615	0.008565	0	0.009443	0	0	0
0	0	0.011626	0.005467	0	0.038742	0	0	0
0	0	0.000001	0.003287	0	0.061591	0	0	0
0	0	0.008049	0.003978	0	0.060645	0	0	0
0	0	0.021369	0.005331	0	0.070532	0	0	0

0	0	0.036814	0.004625	0	0.080724	0	0	0
0	0	0.027661	0.005324	0	0.090796	0	0	0
0	0	0.015346	0.006265	0	0.097316	0	0	0
0	0	0.028615	0.008229	0	0.038742	0	0	0
0	0	0.022824	0.003345	0	0.061591	0	0	0
0	0	0.020579	0.004262	0	0	0	0	0
0	0	0.029689	0.00405	0	0.070532	0	0	0
0	0	0.029652	0.006515	0	0.080724	0	0	0
0	0.08498	0.043478	0.003733	0	0	0	0	0
0	0.0428	0.013116	0.00502	0	0	0	0	0
0	0.04809	0.073794	0.00556	0	0	0	0	0
0	0.037322	0.074859	0.003801	0	0	0	0	0
0	0.024995	0.067509	0.004112	0	0	0	0	0
0	0.022824	0.062062	0.004617	0	0	0	0	0
0	0.093546	0.052091	0.00665	0	0	0	0	0
0	0.0137	0.073794	0.00556	0	0	0	0	0
0	0	0.007051	0.001882	0.008046	0	0.012334	0	0
0	0	0.008135	0.002076	0.010337	0	0.004698	0	0
0	0	0.012334	0.001625	0.018819	0	0.041311	0	0
0	0	0.004698	0.003989	0.023301	0	0.021935	0	0
0	0	0.041311	0.001962	0.001651	0	0.030085	0	0
0	0	0.021935	0.001532	0.001955	0	0.017324	0	0
0	0	0.030085	0.000715	0.000946	0	0.015128	0	0
0	0	0.017324	0.002374	0.003761	0	0.045028	0	0
0	0	0.031642	0.001754	0.002355	0	0.022098	0	0
0	0	0.010971	0.003921	0.023301	0	0.01266	0	0
0	0	0.009588	0.003902	0.001651	0	0.011964	0	0
0	0	0.014184	0.002706	0.001955	0	0.012474	0	0
0	0	0.029351	0.003112	0.000946	0	0.008135	0	0
0	0.084975	0.074131	0.001962	0	0	0	0	0
0	0.042791	0.062194	0.001532	0	0	0	0	0
0	0.048083	0.073009	0.000715	0	0	0	0	0
0	0.093563	0.067324	0.002374	0	0	0	0	0
0	0.013694	0.053164	0.001754	0	0	0	0	0
0		0.032169	0	0	0	0	0	0
0		0.031176	0	0	0	0	0	0
0		0.030118	0	0	0	0	0	0
0		0.031056	0	0	0	0	0	0
0		0.032118	0	0	0	0	0	0
0		0.034181	0	0	0	0	0	0
0		0.034005	0	0	0	0	0	0
0		0.036917	0	0	0	0	0	0
0		0.03684	0	0	0	0	0	0
0		0.028571	0	0	0	0	0	0

18	19	20	21	22	23	24	25	26
0.029561	0	0	0.181189	0	0	0	0	0
0.029824	0	0	0.171526	0	0	0	0	0
0.021674	0	0	0.000001	0	0	0	0	0
0.021007	0	0	0.221648	0	0	0	0	0
0.024176	0	0	0.191594	0	0	0	0	0
0.029978	0	0	0.146886	0	0	0	0	0
0.020172	0	0	0.139558	0	0	0	0	0
0.024148	0	0	0.000001	0	0	0	0	0
0.026415	0	0	0.157967	0	0	0	0	0
0.028583	0	0	0.155989	0	0	0	0	0
0.031663	0	0	0.150644	0	0	0	0	0
0.029884	0	0	0.159738	0	0	0	0	0
0.024071	0	0	0.14212	0	0	0	0	0
0.024374	0	0	0.014824	0	0	0	0	0
0.02835	0	0	0.15165	0	0	0	0	0
0	0	0	0.158403	0	0	0	0	0
0.02176	0	0	0.141024	0	0	0	0	0
0	0	0	0.034839	0	0	0.095446	0	0.106254
0	0	0	0.017299	0	0	0.066563	0	0.053201
0	0	0	0.027166	0	0	0.079619	0	0.082592
0	0	0	0.013259	0	0	0.060409	0	0.084901
0	0	0	0.028446	0	0	0.173506	0	0.069592
0	0	0	0.027741	0	0	0.040423	0	0.080298
0	0	0	0.026741	0	0	0.060423	0	0.070331
0	0	0	0.025	0	0.004394	0	0	0.062512
0	0	0	0.024777	0	0.003945	0	0	0.0731
0	0	0	0.024488	0	0.006025	0	0	0.059341
0	0	0	0.000001	0	0.003177	0	0	0.086413
0	0	0	0.033526	0	0.002643	0	0	0.08901
0	0	0	0.030595	0	0.010267	0	0	0.072102
0	0	0	0.021939	0	0.00456	0	0	0.042709
0	0	0	0.036983	0	0.007581	0	0	0.06076
0	0	0	0.034822	0	0.008611	0	0	0.064936
0	0	0	0.000001	0	0.008314	0	0	0.07285
0	0	0	0.03279	0	0.004785	0	0	0.052361
0	0	0	0.033669	0	0.006535	0	0	0.071068
0	0	0	0.034022	0	0.005121	0	0	0.058513
0	0	0	0.000001	0	0.004923	0	0	0.071131
0	0.018875	0	0.018648	0	0	0	0	0.057014
0	0.01569	0	0.019465	0	0.004369	0	0	0.056287
0	0	0	0.018857	0	0.002615	0	0	0.057018
0	0.013666	0	0.01677	0	0.002639	0	0	0.05616
0	0.018281	0	0.018323	0	0.002587	0	0	0.047912
0	0.01229	0	0.000001	0	0.004138	0	0	0.078621
0	0.01808	0	0.02376	0	0.006739	0	0	0.10213
0	0.014619	0	0.030058	0	0.002493	0	0	0.12734
0	0	0	0.027717	0	0.002832	0	0	0.07028
0	0.014209	0	0.023563	0	0.003099	0	0	0.058936
0	0.021229	0	0.029814	0	0	0	0	0.057162

0	0	0	0.025242	0	0.004874	0	0	0.051476
0	0.017003	0	0.023861	0	0.004151	0	0	0.0441
0	0.01974	0	0.021995	0	0.004265	0	0	0.037931
0	0.005166	0	0.01068	0	0.002251	0	0	0.031069
0	0.006508	0	0.007495	0	0.002162	0	0	0.034163
0	0.004116	0	0.008526	0	0.003327	0	0	0.028412
0	0.006137	0	0.011505	0	0.002158	0	0	0
0	0.00757	0	0.008301	0	0.003681	0	0	0.029426
0	0	0	0.054081	0.068287	0	0	0.021935	0.053997
0	0	0	0.051177	0.068287	0	0	0.030085	0.05319
0	0	0	0.061854	0.051821	0	0	0.017324	0.053471
0	0	0	0.030064	0.067238	0	0	0.02516	0.066028
0	0	0	0.030784	0.070338	0	0	0.023301	0.08927
0	0	0	0.059659	0.064196	0	0	0.019936	0.052713
0	0	0	0.040758	0.054422	0	0	0.02948	0.051506
0	0	0	0.061854	0.062182	0	0	0.0365	0.053529
0	0	0.021052	0.078111	0	0	0.013884	0	0.135923
0	0	0.016623	0.075478	0	0	0.012323	0	0.083126
0	0	0.01643	0.051317	0	0	0.012871	0	0.069104
0	0	0.013879	0.102211	0	0	0.014375	0	0.098747
0	0	0.012206	0.039771	0	0	0.025435	0	0.058269
0	0	0.01087	0.02516	0	0	0.017587	0	0.043014
0	0	0.023078	0.031697	0	0	0.015724	0	0.049317
0	0	0.013041	0.025099	0	0	0.014212	0	0.043527
0	0	0.013041	0.038402	0	0	0.020981	0	0.054926
0	0	0.013879	0.053068	0	0	0.053013	0	0.065349
0	0	0.012206	0.038474	0	0	0.029091	0	0.061841
0	0	0.01087	0.032906	0	0	0.041327	0	0.020371
0	0	0.023078	0.057612	0	0	0.10664	0	0.072796
0	0	0	0.053977	0.05182	0	0	0.0135	0.058273
0	0	0	0.062516	0.063435	0	0	0.03409	0.043183
0	0	0	0.04317	0.07395	0	0	0.023701	0.050254
0	0	0	0.060129	0.05599	0	0	0.02198	0.044317
0	0	0	0.048472	0.06112	0	0	0.031085	0.054982
0	0	0	0.0233	0.01537	0	0	0.06925	0.01611
0	0	0	0.02425	0.01717	0	0	0.098053	0
0	0	0	0.031373	0.01105	0	0	0.053702	0.017308
0	0	0	0.034015	0.02094	0	0	0.07603	0.013028
0	0	0	0.03201	0.01834	0	0	0.05137	0.013151
0	0	0	0.031016	0.0148	0	0	0.07028	0.01982
0	0	0	0.030983	0.007958	0	0	0.05844	0.01084
0	0	0	0.029302	0.023019	0	0	0.063584	0
0	0	0	0.031583	0	0	0	0.0948	0
0	0	0	0.032143	0.015	0	0	0.065	0

27	28	29	30	31	32	33	34	35
0.001305	0	0.047204	0	0	0	0	0	0
0.004167	0	0.052156	0	0	0	0	0	0
0.003479	0	0.044419	0	0	0	0	0	0
0.001352	0	0.041245	0	0	0	0	0	0
0.001522	0	0	0	0	0	0	0	0
0.010024	0	0.041979	0	0	0	0	0	0
0.003396	0	0.035017	0	0	0	0	0	0
0.008818	0	0.056523	0	0	0	0	0	0
0.006289	0	0.044121	0	0	0	0	0	0
0.009653	0	0.06956	0	0	0	0	0	0
0.007462	0	0.063598	0	0	0	0	0	0
0.003429	0	0	0	0	0	0	0	0
0.008808	0	0.083013	0	0	0	0	0	0
0.003296	0	0.068273	0	0	0	0	0	0
0.002251	0	0.044929	0	0	0	0	0	0
0.00469	0	0.050053	0	0	0	0	0	0
0.000001	0	0.075506	0	0	0	0	0	0
0.015741	0	0.350891	0.021628	0	0.106263	0	0.019341	0.106208
0.014729	0	0.348591	0.014176	0	0.053155	0	0.007985	0.052987
0.017742	0	0.411076	0.018132	0	0.082637	0	0.029546	0.082492
0.018728	0	0.213627	0.018545	0	0.084896	0	0.024207	0.085024
0.018743	0	0.231024	0.018541	0	0.069654	0	0.051005	0.070154
0.015569	0	0.400626	0.016732	0	0.080329	0	0.050312	0.079862
0.016569	0	0.470626	0.016732	0	0.070329	0	0.040312	0.070385
0.041493	0.034858	0.457987	0.03019	0.089206	0.06142	0	0.021955	0.062172
0.000001	0.031242	0.423714	0.040459	0.088763	0.072503	0	0.033098	0.071974
0.026621	0.02948	0.399162	0.035818	0.079336	0.058529	0	0.031064	0.05863
0.03113	0.032781	0.433174	0.040529	0.087813	0.086594	0	0.050478	0.085793
0.027505	0.034741	0.468874	0.045294	0	0.079112	0	0.048243	0.080124
0.031386	0.033853	0.479665	0.083503	0.101833	0.071741	0	0.026251	0.071468
0.037714	0.022906	0.372834	0	0.105995	0.041653	0	0.032243	0.040979
0.042642	0.03567	0.425548	0.077324	0.100326	0	0	0.026209	0.06076
0.048625	0.03952	0.533515	0.070245	0.103618	0.065802	0	0.057416	0.066031
0.043421	0.038116	0.519765	0.054497	0.106416	0.075494	0	0.021237	0.059725
0.039568	0.028083	0.456444	0.071159	0.079547	0.0517	0	0.0487	0.060395
0.000001	0.052337	0	0.076966	0.080304	0.070659	0	0.032504	0
0.039584	0.040463	0.462441	0.064747	0.091636	0.059457	0	0.032689	0.061273
0.04005	0.00474	0.446413	0.055821	0.069546	0.071016	0	0.0489	0.069042
0.017153	0.022919	0.343247	0.012781	0.089206	0.056878	0.019565	0.032133	0.057033
0.012647	0.018185	0.302401	0.009182	0.088763	0.056292	0.019598	0.027795	0.055567
0.01225	0.017498	0.295631	0.00867	0.079336	0.056997	0.019731	0.028718	0.056397
0.011199	0.016154	0.282091	0.010787	0	0.048448	0.016969	0	0.048649
0.015333	0.02344	0.3543	0.013362	0.092885	0.048882	0.018278	0.026966	0.048087
0.012649	0.008458	0.362429	0.008259	0.101833	0	0.016308	0.026436	0.076128
0.021003	0	0.427862	0.013758	0.153618	0.10205	0.015986	0.045966	0.110413
0.02809	0.020557	0.379197	0.014775	0.136416	0.128628	0.012012	0.045817	0.12793
0.01412	0.01523	0.3143	0.007204	0.079547	0.06979	0.013507	0.018634	0.078396
0.013483	0.015426	0.272958	0.011018	0.100304	0.059021	0	0.025066	0.05894
0.012908	0.018832	0.327475	0.018287	0.151636	0.057049	0.012593	0.026793	0.056946

0.012043	0.019292	0.347537	0.028452	0.054546	0.052015	0.010481	0.015696	0.051923
0.010824	0.020285	0	0.028631	0.087813	0.043995	0.006465	0.015119	0.044101
0.016678	0.025369	0.395706	0.017871	0	0.038769	0.004841	0.012779	0
0.000001	0.00462	0.134533	0.029712	0.131833	0.032891	0.009473	0.175844	0.033153
0.016723	0.004783	0.108315	0.038491	0.135995	0.035474	0.007772	0	0.032874
0.013772	0.018559	0.115252	0.018557	0.140326	0.02835	0.006206	0.016093	0.028471
0.016171	0.00282	0.104072	0.032105	0.153618	0.034472	0.009556	0.020972	0.035109
0.017095	0.034786	0.109313	0.043087	0.136416	0.029538	0.00741	0.016708	0.028926
0.046573	0	0.440684	0.049295	0	0.054013	0	0.056053	0.05412
0.047141	0	0.531045	0.051513	0	0.053243	0	0.05306	0.052992
0.061243	0	0.556447	0.063867	0	0.053555	0	0.078344	0.053498
0.039848	0	0.404748	0.040343	0	0.065769	0	0.067891	0.066031
0.074974	0	0.528534	0.052984	0	0.089393	0	0.062882	0.089026
0.06312	0	0.471561	0.050862	0	0.052695	0	0.073349	0.052479
0.048458	0	0.430004	0.055031	0	0.051451	0	0.064084	0.051413
0.061243	0	0.656447	0.063867	0	0.053555	0	0.078344	0.053496
0.026526	0	0.333599	0.017369	0	0.136799	0	0.048794	0.13781
0.024336	0	0.208087	0.031173	0	0.083145	0	0.021652	0.082974
0.01875	0	0.170626	0.015128	0	0.069017	0	0.011955	0.068945
0.02951	0	0.235285	0.045028	0	0.099336	0	0.036996	0.098732
0.016291	0	0.212339	0.022098	0	0.058312	0	0.015953	0.057926
0.011662	0	0.188232	0.01266	0	0.042984	0	0.013726	0.043068
0.008006	0	0.148028	0.011964	0	0.049277	0	0.009223	0.048952
0.013041	0	0.201338	0.012474	0	0.043631	0	0.015574	0.042859
0.013657	0	0.189305	0.020994	0	0.055222	0	0.015772	0.054908
0.020404	0	0.363123	0.018503	0	0.065534	0	0.015107	0.065426
0.018194	0	0.365102	0.016735	0	0.061758	0	0.016432	0.061582
0.009043	0	0.088703	0.016982	0	0.019905	0	0.007847	0.020173
0.034265	0	0.328389	0.03246	0	0.072801	0	0.025855	0.073104
0.046291	0	0.212339	0.022098	0	0.058312	0	0.015953	0.058192
0.051166	0	0.188232	0.01266	0	0.042984	0	0.013726	0.043078
0.048006	0	0.148028	0.011964	0	0.049277	0	0.009223	0.049252
0.061304	0	0.201338	0.012474	0	0.043631	0	0.015574	0.044069
0.057398	0	0.189305	0.020994	0	0.055222	0	0.015772	0.054973
0.013551	0	0	0	0	0.017942	0	0	0.015308
0.01347	0	0.411242	0	0	0.0143	0	0	0.01945
0.013604	0	0	0	0	0	0	0	0.01753
0.015444	0	0.421904	0	0	0.021239	0	0	0.01283
0.01548	0	0.401904	0	0	0.018251	0	0	0.013281
0.015081	0	0.621904	0	0	0	0	0	0.021051
0.019608	0	0.631806	0	0	0.01905	0	0	0.01051
0.018995	0	0.505117	0	0	0.014571	0	0	0
0.01802	0	0.505117	0	0	0	0	0	0.01619
0.018286	0	0.542857	0	0	0.01284	0	0	0

36	37	38
0.004159	0.007876	0.024113
0.00261	0.008202	0.023947
0	0.004177	0.025673
0.003304	0.004543	0.026735
0.002752	0.007642	0.021674
0.004244	0.001604	0
0.008042	0.009796	0.024176
0.007719	0.005426	0.029978
0.004996	0.00977	0.020172
0.008341	0.004763	0.029884
0.003542	0	0.024071
0.00141	0.003274	0.024374
0.002061	0.004322	0
0.00106	0.003432	0.024776
0	0.007045	0.02176
0.005467	0.005467	0.016088
0.002788	0.002788	0.019082
0	0	0
0	0	0
0	0	0
0	0	0
0	0	0
0	0	0
0	0	0
0	0	0
0	0	0
0	0	0
0	0	0
0	0	0
0	0	0
0	0	0
0	0	0
0	0	0
0	0	0
0	0	0
0	0	0
0	0	0
0	0	0
0	0	0
0	0	0
0	0	0
0	0	0
0	0	0
0	0	0
0	0	0
0	0	0
0	0	0
0	0	0
0	0	0

0	0	0
0	0	0
0	0	0
0	0	0
0	0	0
0	0	0
0	0	0
0	0	0
0	0	0
0	0	0
0	0	0
0	0	0
0	0	0
0	0	0
0	0	0
0	0	0
0	0	0
0	0	0
0	0	0
0	0	0
0	0	0
0	0	0
0	0	0
0	0	0
0	0	0
0	0	0
0	0	0
0	0	0
0	0	0
0	0	0
0	0	0
0	0	0
0	0	0
0	0	0
0	0	0
0	0	0
0	0	0
0	0	0
0	0	0
0	0	0
0	0	0
0	0	0
0	0	0
0	0	0
